# Pan-cancer analysis of transcripts encoding novel open-reading frames (nORFs) and their potential biological functions

**DOI:** 10.1038/s41525-020-00167-4

**Published:** 2021-01-25

**Authors:** Chaitanya Erady, Adam Boxall, Shraddha Puntambekar, N. Suhas Jagannathan, Ruchi Chauhan, David Chong, Narendra Meena, Apurv Kulkarni, Bhagyashri Kasabe, Kethaki Prathivadi Bhayankaram, Yagnesh Umrania, Adam Andreani, Jean Nel, Matthew T. Wayland, Cristina Pina, Kathryn S. Lilley, Sudhakaran Prabakaran

**Affiliations:** 1 Department of Genetics, University of Cambridge, Downing Site, Cambridge, CB2 3EH UK; 2 Department of Biology, Indian Institute of Science Education and Research, Pune, Maharashtra 411008 India; 3 Cancer and Stem Cell Biology Programme, and Centre for Computational Biology, Duke-NUS Medical School, Singapore, 169857 Singapore; 4 Cambridge Centre for Proteomics, Department of Biochemistry, University of Cambridge, Tennis Court Road, Cambridge, CB2 1QR UK; 5 Department of Zoology, University of Cambridge, Downing Street, Cambridge, CB2 3EJ UK; 6 Department of Haematology, Cambridge Biomedical Campus, Cambridge, CB2 0PT UK

**Keywords:** Systems biology, Genetics

## Abstract

Uncharacterized and unannotated open-reading frames, which we refer to as novel open reading frames (nORFs), may sometimes encode peptides that remain unexplored for novel therapeutic opportunities. To our knowledge, no systematic identification and characterization of transcripts encoding nORFs or their translation products in cancer, or in any other physiological process has been performed. We use our curated nORFs database (nORFs.org), together with RNA-Seq data from The Cancer Genome Atlas (TCGA) and Genotype-Expression (GTEx) consortiums, to identify transcripts containing nORFs that are expressed frequently in cancer or matched normal tissue across 22 cancer types. We show nORFs are subject to extensive dysregulation at the transcript level in cancer tissue and that a small subset of nORFs are associated with overall patient survival, suggesting that nORFs may have prognostic value. We also show that nORF products can form protein-like structures with post-translational modifications. Finally, we perform in silico screening for inhibitors against nORF-encoded proteins that are disrupted in stomach and esophageal cancer, showing that they can potentially be targeted by inhibitors. We hope this work will guide and motivate future studies that perform in-depth characterization of nORF functions in cancer and other diseases.

## Introduction

Profiling molecular changes between normal and tumor tissues, at the genomic, transcript and protein level, underpins much of our understanding of tumorigenesis and tumor progression. Substantial progress has been made thus far considering known or canonical genes and protein coding regions and, in recent years, much of this research has been driven by large publicly available genomic datasets. Recently, consideration of transcript-level changes within protein coding genes has enabled comprehensive characterization of isoform switching across multiple cancers^1^, and extensive evidence now suggests noncoding transcripts^2^ and driver mutations within noncoding regions^3^ have important and functional roles in cancer by diverse mechanisms^4^. Indeed, the apparent complexity of genomic organization and the diversity of genomic elements with functional relevance in cancer motivates the study of further poorly characterized genomic elements, in the hope of identifying novel therapeutic targets or diagnostic markers.

**Fig. 1:**
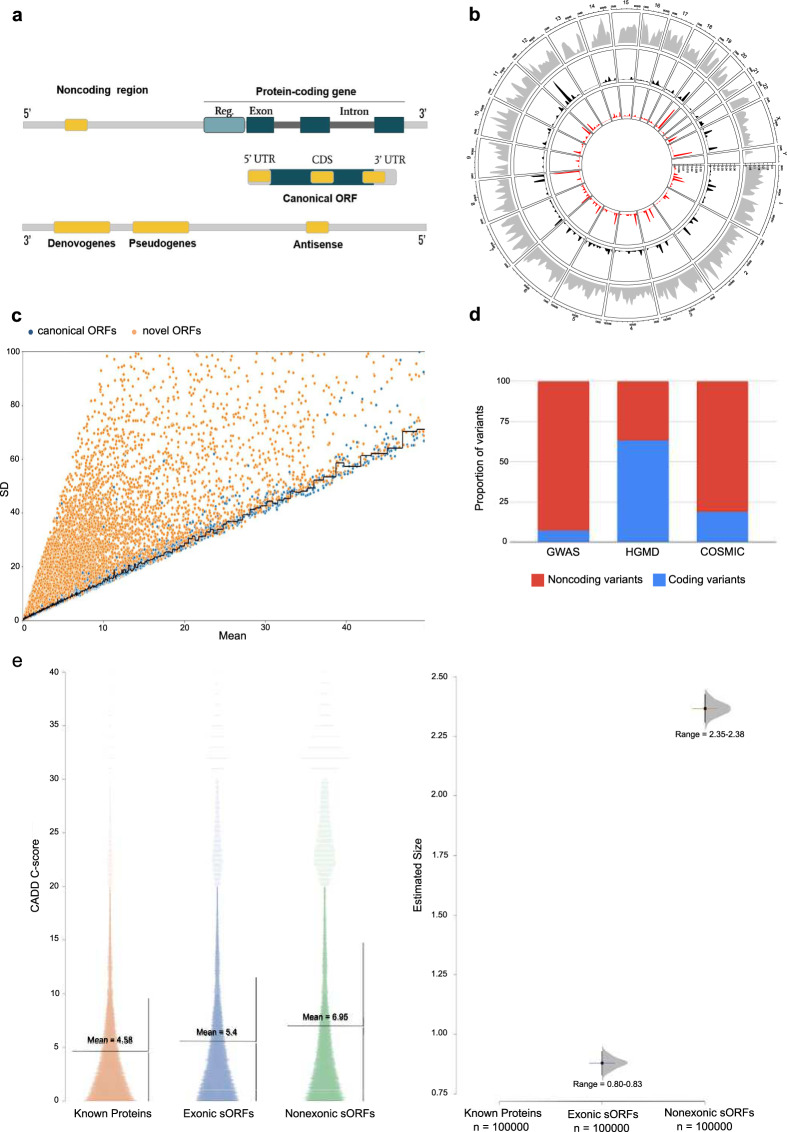
nORFs are important to investigate. **a** Schematic representation of nORFs and their genomic locations. nORFs (yellow boxes) include short ORFs (sORFs) which are ORFs <100 aa, alternative ORFs (altORFs) present in alternative frames of canonical ORFs within protein-coding genes and undefined ORFs which have as of yet not been identified by other studies. These nORFs can be found both within protein-coding (including 5’UTR, 3’UTR, CDS or overlapping CDS and the UTRs) and noncoding regions. They can also be present antisense to genes. ORFs identified within Pseudogenes and Denovogenes are also included under the categorization of nORFs. Reg. regulatory regions. **b** nORFs (from sORFs.org and OpenProt) have been identified throughout the genome on all chromosomes. The gray peaks represent location and density of nORFs on different chromosomes plotted using the R package circlize. Frequently expressed nORFs in the TCGA or GTEx are shown as black peaks, and those identified as differentially expressed are shown in red. **c** Mean Ribo-Seq expression and Ribo-Seq expression standard deviation (SD) have been plotted for human lymphoblastoid cells from RPFdbV2. Canonical ORFs are depicted as blue dots and novel ORFs are depicted by orange dots. The black line shows the median expression SD of canonical ORFs. Not all nORFs have noisy expression values, many have similar SD vs. mean expression values as that of canonical ORFs (cORFs). **d** Proportion of coding (blue) vs. noncoding (red) disease-associated variants within GWAS, HGMD, and COSMIC datasets are shown. Around 90% of disease-associated variants from GWAS, 80% from COSMIC and 40% from HGMD map to noncoding regions. To gain a better understanding of these uncharacterized variants we evaluate those within nORFs. **e** Left panel shows the CADD score distribution and their mean values mapped to known proteins, sORFs in the exonic regions, and sORFs in the non-exonic regions. Right panel is the estimation size plot of the CADD scores showing the mean difference with 95% confidence interval of all variants mapped to exonic sORFs (range 0.80–0.83) and non-exonic sORFs (range 2.35–2.38) with respect to known proteins.

Distinguishing protein-coding and noncoding regions of the genome is challenging, and other uncharacterized or unannotated open-reading frames, which we call novel open-reading frames (nORFs) that includes small open-reading frames (sORFs), are not widely recognized in genomic analysis, largely because conventional algorithms used to identify ORFs impose an arbitrary threshold on ORF length^5–7^. With the advent of deep sequencing strategies in both genomics and proteomics, we are now discovering nORFs that have remained undiscovered or ‘hidden’^8,9^. These nORFs are pervasive throughout the genome, and are observed in both protein-coding and noncoding regions^8,10^ (Fig. 1a). They are variously classified as sORFs^6,11^, which are 1–100 amino acids in length, altORFs^12^, which are proteins in alternate frames to known proteins, Denovogenes^13^ or Orphan genes^14^, and Pseudogenes^15^. nORFs and many ncRNAs have previously been shown to have coding potential^16–20^. These new discoveries challenge traditionally held conservative definitions of an ORF as used until the recent past^21^. Now, better detection methods and broader criteria are helping uncover these increasing repertoire of nORFs by the thousands in every species^22^.

More importantly, a limited number of nORFs have, thus far, been functionally associated with the hallmarks of cancer proposed by Hanahan and Weinberg^23,24^. Notably, the lncRNA HOXB-AS3 has been shown to encode an endogenously translated, small 55 aa peptide, which suppresses tumorigenesis in colon cancer cells^25^. HOXB-AS3 is down-regulated at both the transcript and protein level in colorectal cancer tissue and cell lines, and low protein levels are associated with poor prognosis in colon cancer patients. Likewise, PINT87aa is a circRNA-encoded small peptide, which partially controls cell proliferation and tumorigenesis in cancer cells, is expressed at a reduced level in glioblastoma tissue, and is correlated with tumor grade. Cells over-expressing PINT87aa exhibit decreased tumorigenic potential in animal models^26^. Recent discoveries therefore suggest nORFs may present novel prognostic and diagnostic markers, and those resembling tumor suppressors present particularly exciting therapeutic potential.

To our knowledge, no comprehensive pan-cancer identification and analysis of nORF transcript expression has been performed. This motivated us to identify and determine the expression of transcripts containing nORFs, referred to in this manuscript as nORF transcripts, across multiple cancer tissues from the TCGA and to compare this to expression in normal adjacent tissue (NAT) and normal tissue from the Genotype-Expression (GTEx) project (Supplementary Fig. 1). The UCSC Toil Recompute Compendium^27^ provides processed transcript-level RNA-Seq data from TCGA and GTEx quantified using a unified computational pipeline to remove computational batch effects, and we use this data to perform comparative analysis across samples from both projects. We identify widespread expression of nORF transcripts across multiple cancer types, and show many nORF transcripts are frequently expressed in cancer or corresponding normal tissues. Moreover, we identify nORF transcripts as differentially expressed in cancer tissues, and some nORF transcripts with potential prognostic value.

Having demonstrated that nORFs may be transcriptionally disrupted in cancer, we investigated whether nORF-encoded peptides, despite an increased propensity for structural disorder, can form known protein-like structures with PTMs. We then performed experimental proteogenomic analysis to identify nORFs in B and T cells, and investigated whether these nORFs could be disrupted in cancer. We then in silico screened immune-oncology, targeted oncology, and signal pathway inhibitors against the nORFs that were identified to be expressed only in tumor tissues of stomach adenocarcinoma and esophageal carcinoma and show that these nORF encoded peptides can be targeted for disruption. Our results suggest that nORFs transcripts could be dysregulated in complex diseases, such as cancer and also suggest that their encoded peptides, although they may contain just one domain, could undergo sequence, structural, or regulatory changes.

## Results

### nORFs are pervasively translated

nORFs are typically smaller than canonical ORFs, the peptides or micro-proteins they encode are particularly attractive as putative allosteric cellular regulators, due to their size and the potential specificity of peptide interactions. Therefore, because the accepted nomenclature itself is inconsistent, we previously classified and cataloged all human nORFs from various sources, prioritizing those with strong evidence for translation and distinguishing between nORFs that are in frame and out of frame with overlapping canonical ORFs and released it as an open source database—https://norfs.org/home.

Our curated list of nORFs and other nORF predictions from RPFdb v2.0^28^ illustrate that they are translated from all chromosomes (Fig. 1b). While this indicates that the cellular proteome is much more complex than our current understanding, there is a huge knowledge gap on the putative functions of these nORFs. There have been two lines of speculations about them: on one side, some have dismissed the novel proteins as mere biological noise, while on the other side, some have proposed that such novel proteins confer evolutionary advantage to an organism^29–31^. There is some credence to the hypothesis that they could be biological noise and irrelevant, because functional importance of genes has been shown to anti-correlate with their expression noise in isogenic cells^32^, and our analysis of ribo-seq data from 11 cell lines (Fig. 1c) reveal that many novel proteins are translated with high expression noise (increased standard deviation versus mean) compared to canonical ORFs. However, a small minority of nORFs do have expression noise less than the median expression noise of canonical ORFs, suggesting that at least a small minority of detected nORFs might have important functions (Fig. 1c). In addition, analysis of all the GWAS-associated variants and mutations in the Catalog of Somatic Mutations in Cancer (COSMIC) and Human Gene Mutation Database (HGMD) databases revealed that a significant proportion of variants and mutations map to apparent noncoding regions of the human genome (Fig. 1d). To investigate whether nORF regions could harbor these disease-associated mutations, we mapped COSMIC and HGMD mutations to them. Supplementary Fig. 2a–d shows the top ~20 examples of COSMIC or HGMD mutations mapped to sORFs, Denovogenes, and Pseudogenes, demonstrating that these regions do indeed harbor mutations. To verify whether these mutations are indeed pathogenic we plotted the CADD scores^33^ of all variants that map to sORFs alone^34,35^. Figure 1e left panel, shows the distribution of CADD scores for variants that map to (a) known proteins encoded by known canonical ORFs, (b) sORFs that overlap known ORFs, known as exonic sORFs, and (c) sORFs that are present in noncoding regions. Figure 1e right panel demonstrates that the distribution of mean CADD scores of sORF variants in the noncoding regions are significantly higher than the mean CADD scores of variants that map to exonic sORFs and known proteins. This indicates that the deleterious effects of variants that map to non-exonic sORFs in the noncoding regions are greater than the deleterious effects of variants on known proteins and therefore, nORFs warrant further study.

### Identifying and characterizing transcripts encoding nORFs

To identify transcripts encoding nORFs (nORF transcripts), we extracted genomic coordinates of transcripts quantified in the UCSC Toil pipeline from the GENCODE v23 reference genome annotation, and compared these genomic coordinates with those of nORFs present in the nORFs.org database, using a custom pipeline as described in the “Methods” section (Supplementary Fig. 3). All nORFs present in the database had strong experimental evidence for translation from mass spectrometry or ribosome sequencing. We used GffCompare^36^ to identify transcripts and nORFs with compatible intron chains, and compared genomic coordinates to retain only transcript-nORF mappings where a nORF is completely contained within the transcript genomic start and end position. Transcript expression for nORFs mapping to multiple transcripts would be difficult to interpret, so these nORFs were excluded from this study. We considered only nORFs encoded by noncoding transcripts, as polycistronic transcripts encoding both novel and canonical ORFs would have introduced further complexity in the interpretation of transcript expression. This resulted in the identification of 1488 nORF transcripts.

To determine if nORF transcripts are expressed in any tissue included in the study, we defined an expression threshold of 0.5 counts per million (CPM) across at least 10% of a single tissue. This allowed us to prioritize transcripts that are more likely to be accurately quantified and expressed at a biologically meaningful level. Using this threshold, we identified 926 expressed nORF transcripts for inclusion in this study.

We characterized the genomic properties of all nORF transcripts (Supplementary Fig. 4a) and the 926 nORF transcripts included in this study (Supplementary Fig. 4b), by genomic location and biotype annotation^37^. nORF transcripts are mostly annotated as processed pseudogenes (118, 13%), long intergenic noncoding RNAs (263, 28%), or antisense transcripts (329, 36%), with 216 nORF transcripts falling into other biotype classifications.

We considered genomic distribution and strand bias (Supplementary Fig. 4c and d) to ensure there was no substantial bias in genomic location for the nORF transcripts considered in this study. Across autosomal chromosomes nORF transcripts were consistently distributed, with a small number of nORFs sharing the same start site. However, no transcripts encoding nORFs were identified on the Y chromosome—this is consistent with the lower abundance of genes present on this chromosome. Whilst some chromosomes did exhibit strong strand bias in the number of nORF transcripts identified, namely chromosome 19, overall transcripts were identified consistently in both genomic strands. Comparing the length of novel and canonical ORFs (Supplementary Fig. 4e) revealed a degree of overlap in length, but as expected median nORF length was substantially below that of canonical ORFs, with the majority of nORFs encoding proteins <100 amino acids in length.

Following identification of nORF transcripts, we evaluated transcript mean expression across all GTEx normal tissues included in this study. We showed mean nORF transcript expression compared with canonical protein-coding transcripts and also compared against canonical antisense and lincRNA expression—as these are the two main transcript classifications within which nORF transcripts are identified (Supplementary Figs. 5 and 6). As expected, the median expression of nORF transcripts was below that of canonical protein-coding transcripts, but above that of both noncoding RNA classes. We considered that many nORF transcripts have mean expression comparable with that observed in protein-coding transcripts, which provides confidence that transcripts encoding nORFs may be expressed at an adequate level for translation to occur.

Many nORF transcripts were poorly expressed, with mean CPM values below 0.5. We identified and prioritized nORF transcripts frequently expressed in cancer tissues or the corresponding NAT or GTEx normal tissue. Both cancer and reference normal tissues were considered when identifying frequently expressed nORF transcripts, as we aimed to capture nORF transcripts both up-regulated and down-regulated between cancer and normal tissues. Frequently expressed nORF transcripts were defined as having CPM >0.5 across at least 70% of samples in either cancer or corresponding reference tissue. A representative distribution of expression across samples in cancer tissue and corresponding NAT (Supplementary Fig. 7a) and GTEx normal tissue (Supplementary Fig. 7b) is shown to illustrate this threshold for frequent expression. Two observations provided confidence that a suitable expression threshold had been selected: (i) expression was largely binary, with most nORF transcripts expressed in either every sample or no samples in a tissue and (ii) the number of samples in cancer and normal tissue expressing a given nORF transcript were highly correlated.

When comparing cancer with NAT, we determined 359 out of 926 nORF transcripts were frequently expressed in at least one cancer type; when comparing with GTEx normal tissue, 464 out of 926 nORF transcripts were frequently expressed in at least one cancer type. The number of frequently expressed nORF transcripts identified was fairly consistent across cancer types (Supplementary Fig. 7c, d).

A large proportion of nORF transcripts were frequently expressed across all cancer types—109 nORF transcripts for cancer and NAT; 115 nORF transcripts for cancer and GTEx normal tissue. On the other hand, comparatively few nORF transcripts were frequently expressed in any particular subset of cancer types—for example, just 14 nORF transcripts were only frequently expressed in thyroid carcinoma or thyroid NAT. This likely reflects consistent expression of nORF transcripts across tissues. A disproportionate number of nORF transcripts (79) are frequently expressed only in testicular germ cell tumor tissue or GTEx testis tissue, which is consistent with mean transcript expression patterns in testis tissue (Supplementary Fig. 5)—noncoding transcript expression in the testis appears unusually distinct compared with other tissues.

### Identifying differentially expressed nORF transcripts

To identify nORF transcripts dysregulated in cancer, we performed differential expression (DE) analysis for cancer compared with either NAT or GTEx normal tissue. We normalized RNA-Seq expected counts from the UCSC Toil dataset using the trimmed mean of *M*-values (TMM) method^38^ and performed DE analysis using the general linear model (GLM) framework provided by edgeR^39^, as described in the “Methods” section. A fold change threshold of 2 and an adjusted *p*-value threshold of 0.01 were used to identify differentially expressed nORF transcripts. Only frequently expressed nORF transcripts were considered. Corresponding analysis using a fold change threshold of 1.5 is provided in Supplementary Fig. 8.

**Fig. 2:**
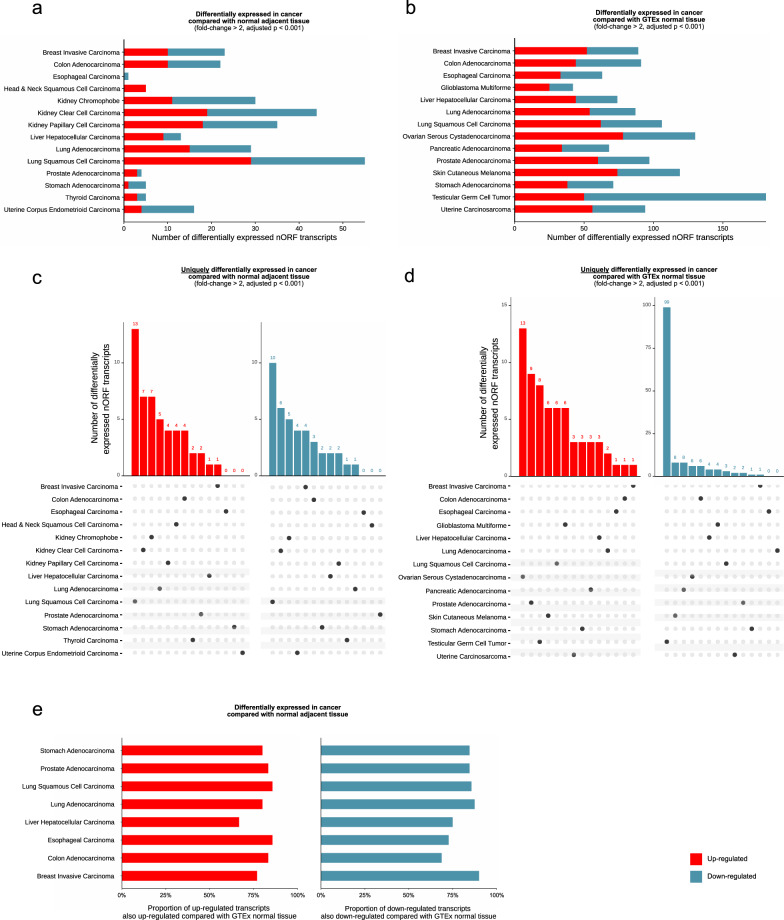
Differentially expressed nORF transcripts in cancer. **a** Total number of differentially expressed nORF transcripts by cancer type compared with NAT. **b** Total number of differentially expressed nORF transcripts by cancer type compared with GTEx. **c** nORF transcripts uniquely up-regulated or down-regulated in a single cancer type compared with NAT. **d** nORF transcripts uniquely up-regulated or down-regulated in a single cancer type compared with GTEx normal tissue. **e** Reproducibility of differential expression results using normal adjacent tissue and GTEx normal tissue. nORF transcripts identified as differentially expressed when comparing cancer tissue with normal adjacent tissue, showing the proportion of nORF transcripts also differentially expressed when comparing cancer tissue with GTEx tissue (left: up-regulated nORF transcripts, right: down-regulated nORF transcripts).

This analysis revealed 152 nORF transcripts as dysregulated in at least a single cancer type when comparing cancer with NAT (Fig. 2a), and 386 as dysregulated when compared with GTEx normal tissue (Fig. 2b). This represented a large proportion of the total number of frequently expressed nORF transcripts. Whilst the number of frequently expressed nORF transcripts was consistent across cancer types, the number of nORF transcripts differentially expressed in each cancer type was diverse. Some cancer types exhibited far more extensive dysregulation of nORF transcription, namely kidney clear cell carcinoma and lung squamous cell carcinoma.

We observed a limited number of nORF transcripts with cancer-type-specific dysregulation. In lung squamous cell carcinoma 13 nORF transcripts were uniquely upregulated, and 10 uniquely down-regulated, when compared against NAT. Kidney clear cell carcinoma, kidney chromophobe, and testicular germ cell tumors also exhibited a large degree of cancer-type-specific dysregulation (Fig. 2c and d). This is consistent with results suggesting that a large number of nORF transcripts were frequently expressed in testis tissue—in testicular germ cell cancer it appears many of these nORF transcripts were down-regulated. Overall, these results demonstrated widespread dysregulation of nORF transcripts across cancers.

To assess the reproducibility of DE results when comparing against NAT or GTEx normal tissue, we investigated differentially expressed nORF transcripts identified in eight cancer types with both types of reference normal tissue. DE relative to GTEx normal tissue consistently revealed a larger number of dysregulated nORF transcripts. Most cancer types showed highly reproducible DE results between the two reference normal tissues (Fig. 2e). Controlling for confounding factors such as age, sex, and ethnicity may help improve the reliability and reproducibility of this DE analysis. A degree of discrepancy was expected, as (i) NAT is affected by the tumor microenvironment and (ii) GTEx normal tissues are more highly represented with larger sample sizes. However, in all but one disease at least 75% of nORF transcripts identified as differentially expressed when using NAT as reference tissue are also identified when using GTEx normal tissue.

### Differentially expressed transcripts and patient overall survival (OS)

**Fig. 3:**
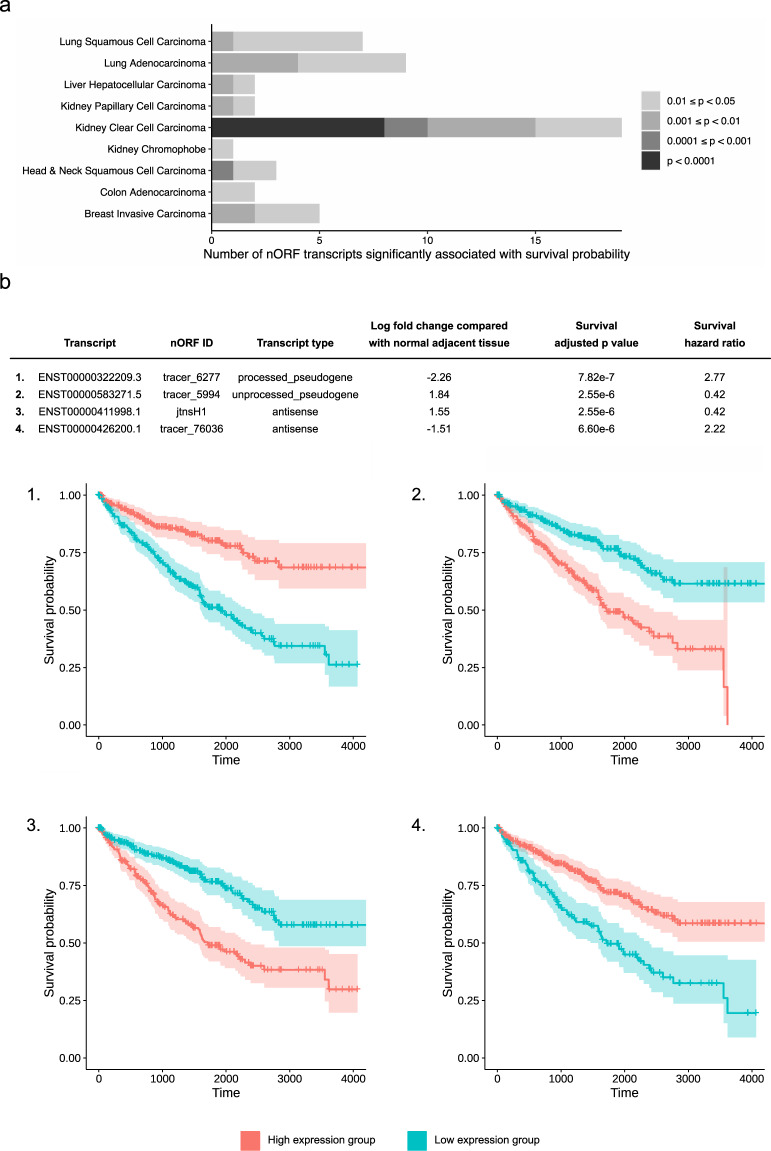
Survival analysis of nORF transcripts. **a** Association of nORF transcript expression with overall patient survival. Number of differentially expressed nORF transcripts significantly associated with survival at different adjusted *p*-value thresholds, by cancer type. **b** Kaplan–Meier curves showing overall patient survival in high and low expression groups for reproducibly differentially expressed nORF transcripts. Showing Kaplan–Meier curves, nORF transcript ID and further transcript details for the four nORF transcripts most significantly associated with prognosis, in Kidney Clear Cell Carcinoma. The cohort was divided into high and low nORF transcript expression groups using the maximally selected rank statistic, and Kaplan–Meier survival curves were generated with a 95% confidence interval. Survival probabilities were compared using the log-rank test and *p*-values adjusted for multiple testing. Overall survival times were fitted to a Cox proportional hazards regression model and hazard ratio calculated from the fitted coefficients.

We have shown that nORF transcripts are frequently expressed across multiple cancer types and reference normal tissues, and that many of these nORF transcripts are transcriptionally dysregulated in cancers. To determine whether any differentially expressed nORF transcripts can be used as prognostic marker, we investigated the relationship between nORF transcript expression and overall patient survival, for nORF transcripts differentially expressed between cancers and NAT. We used survival data for TCGA cohorts provided by the UCSC Toil Recompute Compendium, and divided each cohort into high and low expression groups for each nORF transcript, as detailed in the “Methods” section. We identified 43 nORF transcripts where expression was significantly associated with patient OS in at least one of the 12 cancer types included in this survival analysis, with an adjusted *p*-value threshold of 0.05 (Fig. 3a). This suggested many nORF transcripts may have prognostic value, particularly in kidney clear cell carcinoma.

We investigated further nORF transcripts reproducibly differentially expressed both compared with NAT and GTEx normal tissue (where the GTEx tissue was available for comparison). For a subset of 33 nORF transcripts: (i) the transcript is reproducibly differentially expressed in cancer compared with NAT and GTEx normal tissue, (ii) transcript expression is associated with prognosis (adjusted *p*-value < 0.05) and (iii) transcripts up-regulated in cancer are associated with poor prognosis, and vice versa. Kaplan–Meier survival curves are shown for the nORF transcripts most significantly associated with prognosis, in Kidney Clear Cell Carcinoma (Fig. 3b). We then embarked on a systematic investigation to predict the structure and biological regulation of nORFs and infer their functions.

### nORF-encoded peptides are smaller and have increased disorder

**Fig. 4:**
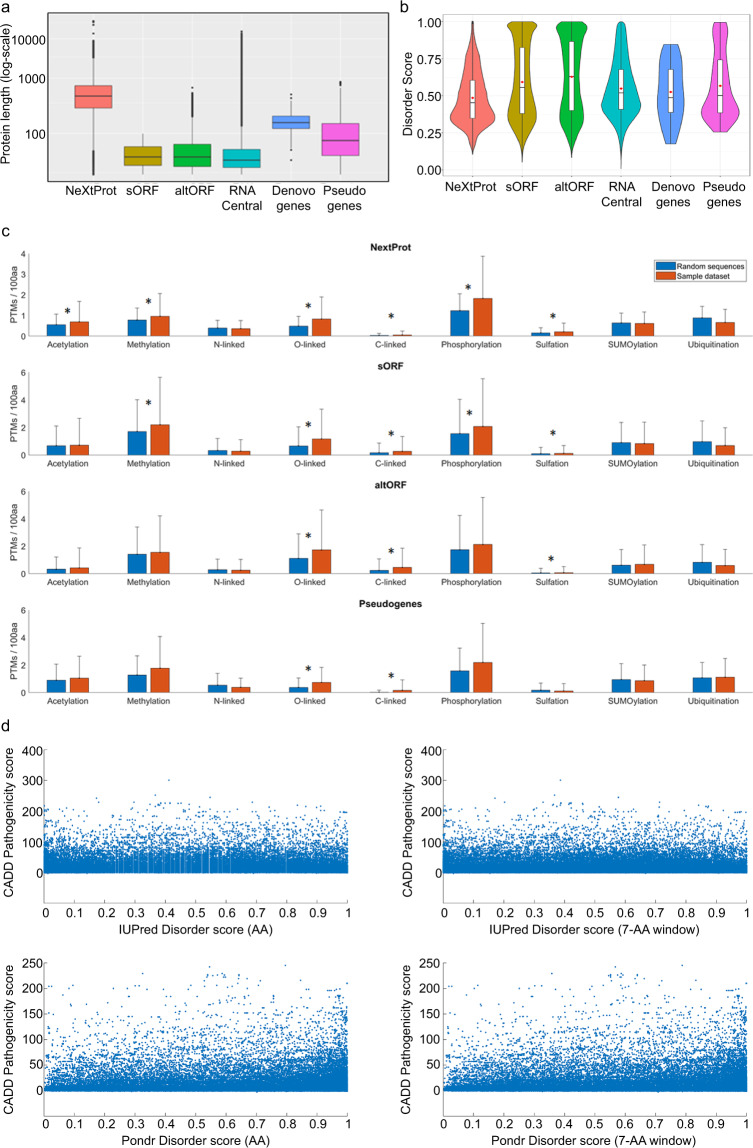
Novel proteins are smaller in length and show increased structural disorder but still have regulatory regions. **a** Amino acid length distribution of known human proteins from NeXtProt, and potential novel proteins encoded by nORFs: sORFs, altORFs, Pseudogenes, Denovogenes, and all possible translated amino acid sequences from RNAcentral. **b** Average disorder scores of proteins in NeXtProt compared to average disorder scores of proteins encoded by nORFs, predicted by PONDR. **c** PTM sites in protein sequences were predicted using the ModPred tool. The predicted densities of nine PTM modifications for each dataset (NeXtProt, sORFs, altORFs, or pseudogenes) were compared against the predicted PTM densities in individual control datasets (random AA sequences generated to have the same average amino acid composition and length distribution as the original dataset). **d** Disorder scores were computed at either amino-acid resolution, or for a 7-AA window around the mutated residue. The analysis did not reveal any correlations between CADD scores and predicted disorder scores.

To systematically determine potential functional consequences of mutations in nORF-encoded peptides, we first curated a list of all nORFs that have been identified with evidence of translation from sORF database (http://sorfs.org/database)^11^, altORFs from Openprot^5^, and Pseudogenes with evidence of translation from Xu et al. ^15^. For Denovogenes, we manually curated a list of 42 protein sequences through literature search. Noncoding RNA sequences were downloaded from RNACentral database (http://rnacentral.org)^40^ and putative translated ORFs were obtained. We compared the lengths of translational products from canonical ORFs (NeXtProt) with nORF peptides from sORFs, altORFs, RNACentral, Denovogenes and pseudogenes (Fig. 4a) and found that the nORF peptides are shorter in length than known proteins. As smaller proteins have been known to form elementary structures, we investigated the propensity of these nORF peptides to form structures^41^. We employed two disorder prediction algorithms, PONDR (Fig. 4b) and IUPred (Supplementary Fig. 9a, b), to assess whether these novel proteins are predominantly ordered or disordered, which would directly correlate with their ability to form structures. For both PONDR and IUPred the results consisted of an average disorder score (in the range 0–1) for a protein sequence, and the percentage of disorder for each sequence (Supplementary Fig. 9c). Sequences that had an average disorder score >0.5 were considered “disordered sequences”. The computed bootstrap confidence intervals of mean (and median) average disorder scores showed that the nORF peptides (sORFs, altORFs, RNACentral, Pseudogenes, and Denovogenes) had higher mean (and median) values of disorder than known proteins in NeXtProt (Fig. 4b). Statistical tests (Fisher’s exact test and Chi square test) showed that each of the nORF datasets (except Denovogenes) was enriched for disordered sequences in comparison to proteins in NeXtProt (Supplementary Fig. 10a, b). Supplementary Fig. 11 shows the final number of amino sequences in each novel protein category used in the above analysis.

Some disordered regions have been known to undergo disorder-to-order transitions upon binding to substrates. We used the Anchor program^42^ to investigate whether the novel proteins show increased propensity to form structures. The results of this analysis indicate that novel proteins, except for Denovogenes, show increased anchor scores compared to NeXtProt proteins (Supplementary Fig. 12a). However, we also found a strong positive correlation between average anchor score and average disorder score for most datasets, which is not surprising, since the prediction of binding sites uses biophysical parameters similar to those involved in disorder prediction (Supplementary Fig. 12b).

### nORF peptides could be biologically regulated

Previous evidence suggested that nORF peptides expressed in mouse neurons are indeed biologically regulated^8^ and that they may be enriched for regulatory sites for post-translational modifications (PTMs), such as phosphorylation. Hence we predicted PTM sites in the amino acid sequences from all our curated novel proteins using the ModPred software^43^. For each sequence, we predicted amino acid sites for nine PTMs—phosphorylation, acetylation, methylation, sulfation, SUMOylation, ubiquitination, C-linked, O-linked, and N-linked glycosylation (Fig. 4c). For each dataset (NeXTProt, sORF, altORF, and pseudogenes) we created individual control datasets composed of randomly generated AA sequences with the same average amino acid composition and length distribution as the original dataset. Methylation, glycosylation, and phosphorylation were found to be significantly enriched in some novel protein datasets and NeXtProt proteins, compared to their individual random controls (Fig. 4c). We used the single-tailed Wilcoxon rank sum test to check if the datasets were more enriched for PTMs than their respective random controls. *p*-Values were corrected for multiple hypothesis testing using the Benjamini–Hochberg method. Asterisks in the figure refer to corrected *p*-values < 0.005 Wilcoxon rank sum test. For most PTM types the densities of predicted PTMs was comparable or higher than in the novel proteins versus the NeXtProt database (Fig. 4c). This indicates that the nORF peptides could be subjected to any biochemical regulation just as much as all known proteins. We did not find any correlation between low pathogenicity and higher disorder scores, which indicates that mutation in novel proteins can affect their potential functions (Fig. 4d, Supplementary Fig. 13).

### Experimental identification of nORFs in mouse B and T cells

Because our nORF transcript identification from the TCGA and GTEx datasets relied on computational mapping of nORF regions using a precompiled nORF dataset, we wanted to investigate whether nORFs that we identify in biological cells could be associated with cancer as a proof of principle. To do this we employed a proteogenomics approach combining total RNA sequencing data of naive mouse B and T cells (GSE94671) from the Blueprint consortium with in-house generated proteomics data from a similar experimental design. We generated and analyzed this particular dataset because to identify nORF-encoded peptides we needed to obtain high-quality transcriptomic and proteomic data from the samples.

Briefly, total RNA was extracted from naive B and T cells isolated from the spleen of six male and six female C57BL/6J mice and sequenced (Supplementary Table 1). Similarly, proteins were extracted from naive B and T cells isolated from spleen of a different set of six male and six female BL6 mice and analyzed using mass spectrometry (Supplementary Fig. 14). Using a proteogenomics workflow, illustrated in Supplementary Fig. 15, the following nORFs regions were systematically investigated: (a) sORFs, (b) altORFs, and (c) all other as-yet undefined nORFs. Briefly, all mass spectra obtained from the naive B and T cell proteome were mapped to the following three databases independently and in a sequential order. MS of proteins isolated from the samples were first searched against the mouse UniProt database. To verify the presence of already known nORFs, the spectra unmapped to known proteins, ~60%, were then mapped to a nORF amino acid database generated using the amino acid sequence of these nORFs obtained from sorfs.org and OpenProt. Finally, the remaining unmatched spectra were matched to the custom proteogenomic database created using the sample-specific assembled transcriptome (Supplementary Fig. 15). We used Mascot search engine for searching the spectra against the Uniprot proteins, the nORF amino acid database and the custom transcriptomic database in six frames, performed “on the fly”.

Using this analysis pipeline, we identified 2030 known proteins, and 1658 novel proteins—from 1649 sORFs and 9 altORFs to be translated in B and T cells (Supplementary Fig. 16). Mass spectra that did not match to any of the three databases were further queried by mapping them to the B and T cell-specific proteogenomic nucleotide databases in six frames. Transcriptomic and proteomic database construction and novel protein abundance distribution are illustrated in Supplementary Figs. 17–20. From this analysis 259 noncanonical transcript regions (176 in B cells and 86 in T cells) were identified to be translated with at least two peptides matching per noncanonical transcript (construction of this database is explained in the “Methods” section). A total of 766 peptides were used to identify 259 noncanonical transcript regions as translated. Genomic annotations for 990 out of 1649 sORFs (Supplementary Fig. 21a), 7 out of 9 altORFs (Supplementary Fig. 21b) and 259 undefined nORF regions from 1373 out of 1405 genomic regions (Supplementary Fig. 21c) reveal that most nORFs map to intronic or lncRNA regions.

### Regulation by phosphorylation and potential biological functions of novel proteins from B and T cells

**Fig. 5:**
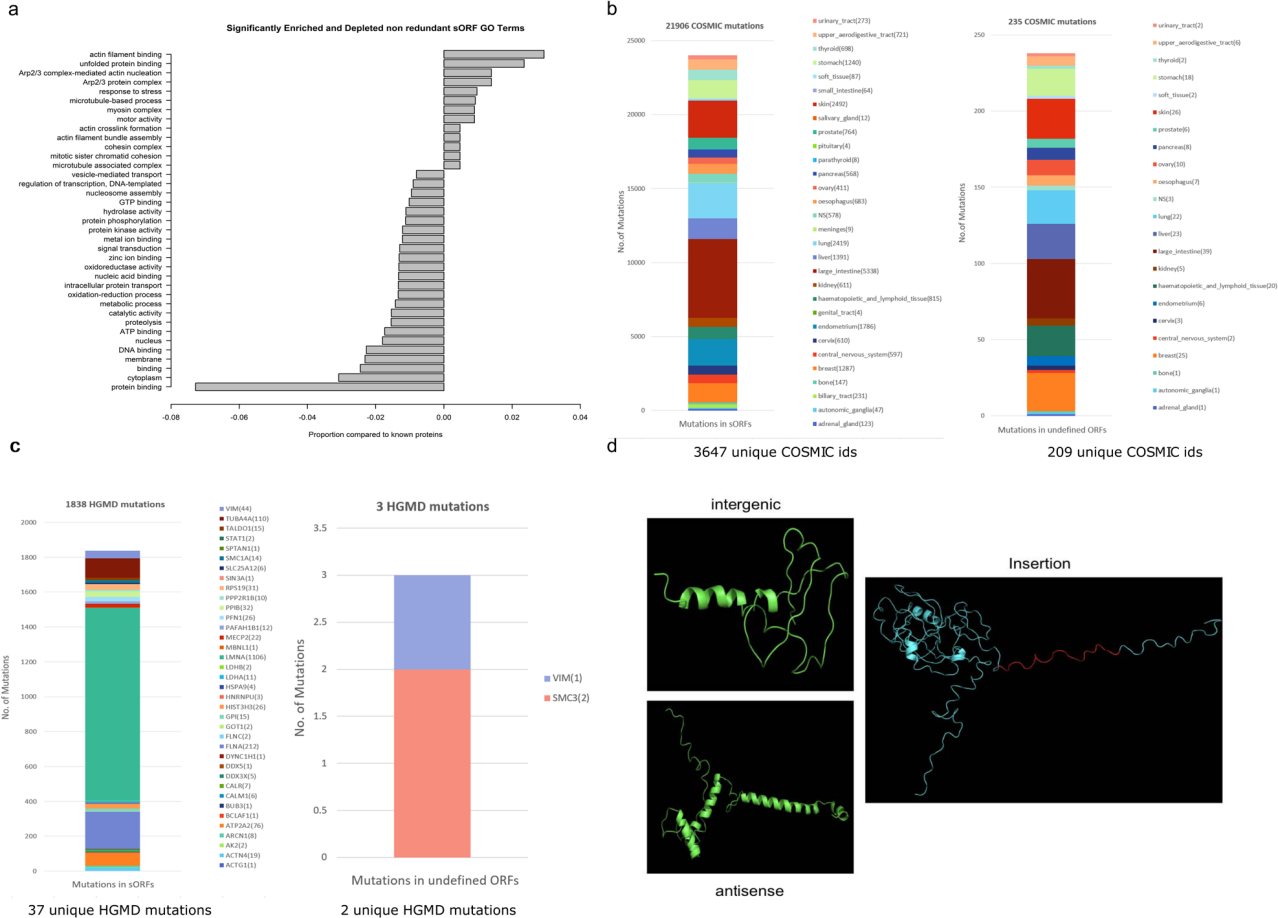
Potential structures, biological functions and mutations mapped to nORFs translated in naive B and T cells. **a** List of significantly enriched or depleted GO terms in sORFs after removal of redundant sORFs as compared to GO terms from known proteins. **b** Results of mapping COSMIC mutations to sORFs (left) and undefined ORFs (right) that are conserved in the human genome identified using tblastn and LiftOver. The total number of mutations identified are represented above the bars and the number of unique mutations is mentioned below the graph. **c** Results of mapping HGMD mutations to sORFs and undefined ORFs that are conserved in the human genome identified using tblastn and LiftOver. The total number of mutations identified are represented above the bars and the number of unique mutations is mentioned below the graph. Disease phenotypes and the number of mapped mutations associated with the genes in the legend are expanded in Supplementary Table 2. **d** (left) Predicted structure of *intergenic* mouse nORF in chr 14, (right) the predicted structure of the original Rps3a1 protein (cyan) with *intron insertion* fragment (red), (bottom) predicted structure of *antisense mouse* nORF, antisense to *Raet1*.

Mass spectrometry analysis of the B and T cell dataset revealed six phosphorylations on sORFs and 297 phosphorylations on the 259 undefined nORF regions (Supplementary Fig. 22). This provides experimental verification that sORFs and nORF-encoded peptides in general can undergo PTMs. To predict putative biological functions of these nORFs from their sequences we used Interproscan. The resulting GO terms gave us a clue to their potential functions (Fig. 5a). GO terms of the 2030 identified known proteins were also analyzed to validate the Interpro-predicted GO terms for nORFs. Expected values based on the GO terms from known proteins with cutoffs of *q* < 0.01 and *p* < 0.01 were used to determine significantly enriched or depleted GO terms for sORFs (Supplementary Fig. 23). We then used GOSim to cluster GO terms based on functional similarities between gene products and the associated GO terms (Supplementary Figs 24 and 25). The results indicate that sORFs are more involved in cytoskeletal or structural cellular functions of the cells than in signaling or protein-binding functions. Although these results indicate functional enrichment, we were not able to identify specific functional roles for the novel proteins. Therefore, we looked for indirect evidence for functions of the novel ORFs. To do this, we identified their corresponding conserved novel ORF regions in the human genome and then mapped mutations from COSMIC and HGMD datasets to identify whether the novel ORFs (human orthologs of mouse ORFs) are disrupted in diseases. Figure 5b and c (left panel and right panel) show the number of unique COSMIC and HGMD mutations along with the disease origin of these mutations for sORFs (left panel) and undefined ORFs (right panel) that are conserved in the human genome. List of genes, associated disease phenotype and number of HGMD mutations corresponding to the phenotype are given in Supplementary Table 2.

We predicted structures for 24 sORFs (Supplementary Table 3) and 9 altORFs translated in mouse B and T cells (Supplementary Table 4) using the EVFold pipeline^44^. Figure 5d shows (a) predicted structure of a translated product from the undefined novel ORF in an intergenic region in chr 14, (b) predicted structure of an undefined novel ORF insertion in Rps3a1 ribosomal protein (cyan) with the inserted fragment (red), and (c) predicted structure of an undefined novel ORF product antisense to *Raet1*. All of the above novel ORFs are marked and represented in Integrative Genome Viewer (IGV) in Supplementary Figs. 26–29.

### Dysregulation of nORFs in cancer and screen for inhibitors

**Fig. 6:**
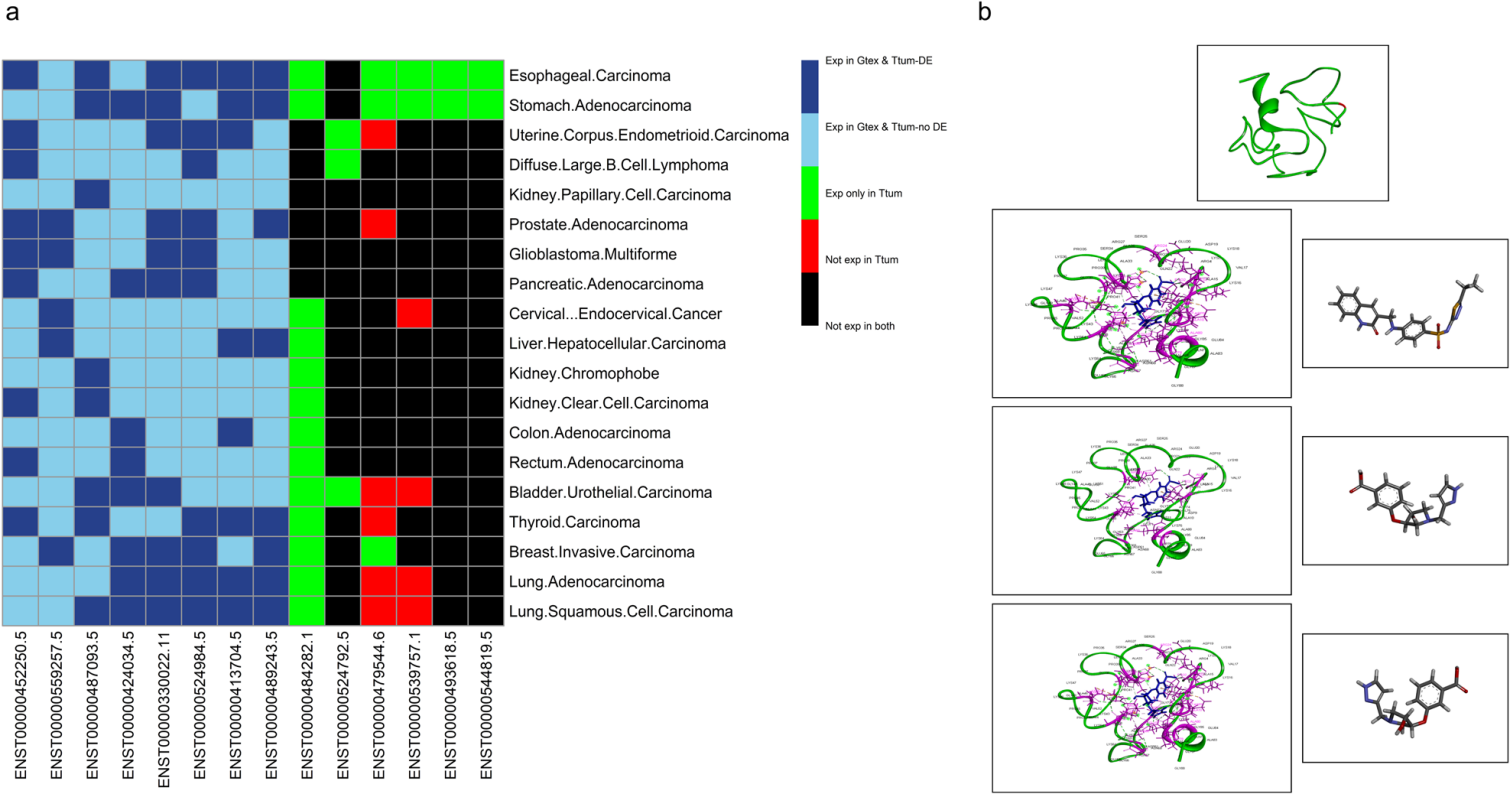
nORFs dysregulated in cancer. **a** Analysis of Xena’s TCGA-TARGET-GTEx dataset to study the expression of the 14 probable ‘cancer markers’ which are expressed differentially in 19 cancers. These 14 markers are non-protein-coding transcripts that translate low-noise nORFs in 11 cell lines as observed from analyzing the ribo-ORF datasets from RPFdb (black—transcripts that are not expressed in both the tumor and matched normal samples; red—transcripts that are not expressed only in tumor samples; green—transcripts that are expressed only in tumor samples; light blue—no differential expression of transcript between the tumor and normal samples; dark blue—differential expression of transcript between the tumor and normal samples) (a transcript is defined to be expressed if it has non-zero expression in at least 25% of the samples). **b** Predicted structure of mPLsORF0000447155, which is a peptide translated from ENST00000427352.1, using EV-Fold, of human ortholog displayed with pymol. Red regions on the structure indicate amino acids which are affected by COSMIC mutations. Supplementary Table 6 shows the mutations associated with this sORF. Below are the structures of the highest scoring ligands of compound 8462, compound 1491, and compound 1355 (right), and that of the complex it makes with the sORF (left) predicted, respectively, from the libraries: Immune-oncology, Targeted Oncology, and Signal pathway inhibitor.

To show that novel proteins are dysregulated in cancer we identified 14 novel ORFs that are identified to be translated with ‘low-noise’ in 11 human cell lines from the ribo-ORF datasets^28^. The expression of these 14 transcripts in cancer was then analyzed using the UCSC Toil RNA-seq Recompute and found to be differentially expressed in 19 of the 33 cancer types, in spite of using a very stringent criteria for this analysis (Fig. 6a). This indicates that they might be dysregulated and have some role in cancer.

Interestingly, ENST00000484282.1 is expressed only in tumor samples and not in their matched healthy tissues across almost 70% of the TCGA cancer types (Fig. 6a). Encoded by the DOP1A gene (DOP1 leucine zipper like protein A; ENSG00000083097), ENST00000484282.1 is annotated as a processed transcript, and therefore, by definition does not contain an ORF. Analysis with the RPFdbv2.0 datasets showed that this transcript translates a ‘low noise’ ORF with an ATG start codon in all the 11 human cell lines analyzed (many of which are cancer cell lines). Thus, this transcript, which is expressed only in tumor samples may potentially express a sORF with some specific function in tumors.

Additionally, to investigate whether novel proteins dysregulated in cancer can be used as therapeutic targets, we predicted structure of the human ortholog of a sORF, mPLsORF0000447155, identified in our B and T cell study, translated from ENST00000427352.1, and identified to be expressed only in the tumor samples of stomach adenocarcinoma and esophageal carcinoma and with two noncoding mutations mapped to them (COSN19210254; COSN8491742) of which; COSN8491742 is identified in only lung samples (Fig. 6b, Supplementary Table 5). We then screened for highest scoring ligands from the asinex library against Immune-oncology (8462 compounds), targeted-oncology (1491 compounds), and signaling pathway inhibitors (1355 compounds). Figure 6b lower panels indicate the top scoring ligands for the above categories. These results reveal that novel proteins are not only dysregulated in cancer but can also be used for diagnostic and for therapeutic purposes.

## Discussion

Through comprehensive analysis of RNA-Seq data from 22 cancer types, we have identified transcripts containing nORFs and demonstrated that many nORF transcripts are frequently expressed in multiple cancers. Additionally, we have shown that many of these nORF transcripts are differentially expressed between cancer and normal tissues, and some of these nORF transcripts are uniquely differentially expressed in specific cancer types. Furthermore, we have shown that expression of some differentially expressed nORF transcripts may have prognostic value. This subset of nORF transcripts indicated that nORFs should be further characterized.

Therefore, we systematically characterized all those nORF peptides that were previously identified and those that we identified in B and T cells, and demonstrate that although they are small and have increased disorder, the proportion of increase is not substantial to affect their structure-forming capabilities. More importantly, we show that nORF peptide expressions are not necessarily noisy, they can be biochemically regulated by PTMs, and more importantly can harbor deleterious mutations that can potentially be targeted with inhibitors. Although based on GO analysis, enrichment of sORFs in cytoskeletal or structural functions were found, it must be noted that this analysis is limited to sORFs and known proteins currently annotated with GO terms. With improved annotation, especially for the unannotated sORFs, the results may indicate different functional category enrichment.

We present convincing evidence that transcripts with the potential to encode nORFs are both frequently and differentially expressed across cancer and normal tissues. However, without evidence for nORF translation in cancer tissues from mass spectrometry or ribosome sequencing, it is not possible to attribute any difference in transcript expression to nORF translation. This is especially limiting given that, for known protein-coding transcripts, the relationship between transcript and protein abundance is complex and influenced by many factors^45^. Indeed, the diverse function of noncoding RNAs at the transcript level is well established in both normal and cancer tissues, and it is currently unclear whether noncoding transcripts containing nORFs may be bi-functional^4^. Limited availability of genome-wide proteomic or ribosome-sequencing data is a key challenge in the identification of nORFs translated in specific cell types or tissues, especially given such work is both expensive and technically challenging. Many large-scale proteomic studies utilize cost effective reverse phase protein arrays, but this approach is generally limited to quantifying expression for a small panel of canonical proteins. Moving forward, translation of nORFs must be systematically confirmed in multiple cancer types—the recently completed proteomic profiling of 375 cancer cell lines^46^ combined with genomic data from the Cancer Cell Line Encyclopedia^47^ present an excellent resource for validation of nORF translation. Peptides translated from nORFs must be validated experimentally and distinct function must be attributed to the peptide and the transcript, as is the case for a limited number of previously characterized nORFs^4,25,26^.

We do recognize that the weakness of our work is that it is built on weak correlations and observing these nORF peptides does not show that they are biologically important. The disruption of nORF expression in disease states such as cancer does not demonstrate that the nORF has anything to do with the cause or mechanism of that disease and the “disease associated” mutations occurring in nORFs is likely incidental. But nonetheless there is already evidence of biochemical functions for some novel proteins, for example, the smallest ORF for which any known function is attributed is just six amino acids long and is in a 5′UTR. It regulates the expression of S-adenosylmethionine decarboxylase in response to polyamine levels^48^. Muscle regeneration^49,50^, phagocytosis^51^, DNA replication^52,53^, cancer^25^, and metabolism^19,54^ are other examples. Despite these examples, the vast majority of nORF-encoded peptides have not been investigated rigorously. Our systematic investigation suggests that nORF peptides are very important to understand, diagnose, and cure complex diseases. Hence, we believe that the unexplored world of nORF peptides represents an untapped opportunity for discovery of new fundamental and translational areas of research. We hope this work will guide and motivate future detailed characterization of novel peptides in cancer and other diseases.

## Methods

### Noise expression analysis of nORFs

To investigate whether the expression of nORFs is noisy, the expression of canonical ORFs was compared to the novel ORFs using 53 studies, 353 samples downloaded from RPFdbv2.0^28^ across 11 human cell lines. Actively translated ORFs, having a footprints with clear sub-codon phasing or triplet periodicity, in each study included in this database, is detected systematically using the RibORF tool^53,55^. Further each ORF entry is annotated with its genomic position, strand, annotated ORF category (canonical, truncated, extended, uORF, overlapping uORF, internal, external, polycistronic, readthrough, non-coding transcripts), length of encoded amino acid, ribosome profiling abundance (RPKMs, raw read counts) and the transcript to which the ORF maps (probable transcript from which ORF is translated). Abundance of each ORF estimated in raw read counts was converted to TPMs for further analysis. The 353 samples were divided into 11 groups based on cell types. Mean and SD of ribo-seq expression TPMs for all samples in each group were calculated; and compared between the canonical and the rest (‘non’-canonical) ORFs. In order to compare SDs of nORFs to cORFs with similar means, the entire range of means was divided into exactly 4000 quantiles based on the means. Every quantile had the same number of ORFs. Within each quantile, the SDs were compared between nORFs and cORFs. ORFs with SDs less than the median SD of cORFs were termed low noise ORFs. A total of 272,229 unique, low-noise ORFs were identified from 11 cell types of which 225,273 were nORFs and rest 46,956 were cORFs.

### TCGA and GTEx transcriptome processing

TCGA and GTEx RNA-Seq and survival data was downloaded from the ‘TCGA TARGET GTEx’ cohort of the UCSC Toil Recompute Compendium^27^. Transcriptome alignment had been performed using STAR (GRCh38) and transcript expression quantified using RSEM, using transcripts present in the GENCODE v23 genome annotation. Transcript-level RSEM expected counts, TCGA survival data and phenotype data were obtained. The GENCODE v23 and corresponding Ensembl v81 genome annotations were downloaded, and transcript and coding sequence properties were extracted from the annotation files using a custom script. RSEM expected counts provided by the UCSC Toil Recompute Compendium were log2(expected_count + 1) transformed, and this transformation was removed to produce raw expected counts for use in this analysis. All data processing was performed using R, R Studio, the R package Tidyverse, and unix command line tools. The Ensembl genome annotation was processed in R using ensembl db^56^, and genomic coordinates were processed using GenomicRanges. Set diagrams were produced using UpSetR.

### TCGA and GTEx normal sample selection

Mappings of TCGA cancer tissue samples to NAT and GTEx normal tissue were extracted from the phenotype data provided by the UCSC Toil Recompute Compendium. We included solid tumor TCGA cancer tissues with at least 50 samples, with matched NAT or GTEx normal tissue with at least 10 or 50 samples, respectively—a less stringent threshold for inclusion was used for NAT because these samples were less abundant. RSEM expected count data was filtered to retain only selected samples and expressed transcripts prior to normalization and DE analysis. A single sample containing missing expected count values was excluded from this analysis.

### Identifying TCGA and GTEx expressed transcripts

Prior to library size normalization and DE analysis, transcripts with poor expression were excluded from analysis. Applying a CPM threshold to identify expressed transcripts prior to TMM normalization and DE analysis has been shown to improve false discovery rate^57^ and is recommended practice for edgeR. Expected counts were transformed to CPM and transcripts are classified as expressed if they had expected count >0.5 CPM in at least 10% of the samples of a single cancer or normal tissue. Expressed transcripts are retained. Best practices for setting thresholds for transcript-level expression are poorly established, and the thresholds used in this study were, whilst informed by the literature, largely arbitrary.

### Selecting matched cancer and normal tissue samples

To characterize the expression of transcripts encoding nORFs across multiple cancer types and corresponding normal tissues, we obtained transcript-level RNA-Seq expression data from the UCSC Toil Recompute Compendium^27^. This dataset includes 11,194 cancer and NAT samples from TCGA and 8003 normal tissue samples from GTEx. We used metadata provided by the UCSC Toil Recompute Compendium to match cancer, NAT, and GTEx normal tissues and determine the number of samples available for each tissue. To ensure consistent and reliable results, we included solid tumor TCGA cancer tissues with at least 50 samples, with matched NAT or GTEx normal tissue containing at least 10 or 50 samples, respectively—a less stringent threshold for inclusion was used for NAT because these samples are less abundant. This resulted in a total of 7885 samples across 22 cancer types from TCGA, together with 677 NAT samples and 4010 GTEx normal samples. The cancer and matching NAT or GTEx normal tissues included in this study are summarized in Supplementary Fig. 30.

NAT and GTEx normal tissues provide non-redundant reference tissues. NAT samples closely resemble cancer samples both as a result of reduced variation in patient differences and sample processing. However, NAT is affected by changes in the tumor microenvironment and samples are less abundant than GTEx normal tissue samples. Seven cancer tissues included in this study are matched to both NAT and GTEx normal tissue which allowed us to determine whether DE results are reproducible across different reference tissues.

### Identifying transcripts containing nORFs

Genomic coordinates of nORFs with experimental evidence for translation were obtained from the nORFs.org database (https://norfs.org/home). Transcript genomic coordinates were obtained from the GENCODE v23 reference annotation. GffCompare was used to identify open-reading frames and transcripts with completely matching intron chains. GffCompare performs stringent filtering to detect and remove redundant input transcripts, and this deduplication is described in detail in the documentation. Specifically, to achieve stringent deduplication of nORFs, GffCompare was run with nORF coordinates as the ‘reference set’ and transcript coordinates as the ‘query set’, with default parameters. The resultant ‘.refmap’ file containing information on overlaps between nORF and transcript coordinates was processed in R and annotated. nORF-transcript mappings identified by GffCompare were filtered to retain only those with a complete intron chain match, and for which the genomic coordinates of the nORF were completely contained within the transcript. nORFs present in multiple transcripts were excluded. Transcript biotypes were extracted from the GENCODE annotation file and open-reading frames contained in protein-coding transcripts (transcripts with biotype: “protein_coding”, “IG_C_gene”, “IG_D_gene”, “IG_J_gene”, “IG_V_gene”, “TR_C_gene”, “TR_D_gene”, “TR_J_gene”, “TR_V_gene”) and rRNA transcripts were excluded. Novel and canonical ORF lengths were determined using ensembldb.

### RNA sequencing normalization

Normalization and DE were performed separately for comparison of cancer tissue with NAT and with GTEx normal tissue. RNA-Seq expected counts were normalized across samples using the TMM^38^ method to normalize for read depth and composition. As comparisons in DE were not made across transcripts, no normalization was introduced for effective transcript length.

### Identifying frequently expressed transcripts

To identify frequently expressed transcripts, CPM values were calculated across all expressed transcripts following TMM normalization using edgeR. Transcripts were classed as frequently expressed if they had CPM >0.5 in at least 70% of the samples in the normal or cancer tissue of interest.

### Transcript DE

Transcript DE was performed using all expressed transcripts to provide correct significance testing and improve reliability of dispersion estimation. The R package edgeR^39^ was used to perform DE analysis using a GLM framework—this package was chosen as it is (i) highly cited, (ii) suitable for transcript-level analysis, (iii) compatible with non-integer expected counts from RSEM, and (iv) exhibits fast performance on large datasets. A simple additive model with no intercept was constructed, with normal reference tissues and cancer tissues each represented by a single coefficient. No covariates, such as ethnicity, sex, age, or tumor grade, were controlled for in this DE analysis, but the GLM framework in edgeR was chosen because it would allow for control of covariates in follow-up analysis. The process used for DE analysis is detailed in the edgeR manual. Briefly, transcript-wise dispersions were estimated under the GLM framework using the Cox–Reid profile-adjusted likelihood method, which takes into account multiple factors by fitting the described model. A negative binomial model was fitted for each transcript, and thresholded hypotheses were tested to provide meaningful *p*-values and reliable control of false discovery rate. A fold-change threshold of 1.5 or 2 was used to identify differentially expressed transcripts, with an adjusted *p* value threshold of 0.001. Coefficients representing cancer tissues and their corresponding normal reference tissues were compared under this framework. The Benjamini and Hochberg method was used to adjust *p*-values for multiple testing and control false discovery rate.

### Patient OS analysis

OS analysis was performed using the R packages survival^58^ and survminer^59^. nORF transcripts are included in survival analysis if they were differentially expressed in the cancer type of interest compared with NAT, and were expressed at >0.5 CPM in at least 70% of the samples in the cancer tissue cohort. For each cancer type and for the nORF transcript considered, the cohort was split into high and low expression groups. Groups were selected which were best segregated based on OS, using the Maximally Selected Rank Statistic, with at least 30% of patients assigned to each expression group to avoid forming groups with a small number of patients. Kaplan–Meier curves were generated and curves were compared using a log-rank test. The Benjamini and Hochberg method was used to adjust *p*-values for multiple testing and control false discovery rate. A Cox proportional hazards regression model was fitted to OS data and hazard ratios were derived from the model coefficients. Both the Kaplan–Meier and Cox proportional hazards regression models assume proportional hazards, where the hazard ratio between the high and low expression groups remains constant over time.

### Protein domain prediction

Nucleotide sequence was extracted from nORF genomic coordinates and the reference genome (GRCh38) using BEDTools getfasta^60^ and translated into amino acid sequence using EMBOSS Transeq^61^. Protein domains were predicted from amino acid sequence using InterProScan^62^.

### Data set collection

We first curated a list of all nORFs that have been identified with evidence of translation. We obtained sequences for known and verified human proteins from NeXtProt (https://www.nextprot.org/)^63^, sequences for sORFs from the sORF database (http://sorfs.org/database)^11^, sequences for altORFs from Roucou’s lab^12^ (http://haltorf.roucoulab.com/ but updated as http://haltorf.roucoulab.com to the new URL https://www.openprot.org), and sequences of Pseudogenes with evidence of translation from Xu et al. ^15^. For Denovogenes, we manually curated a list of 42 protein sequences through literature search.

For conservative measurements of disorder scores, we discarded protein sequences <30 amino acids in length from all the above datasets, since these were likely to be enriched for disorder. Noncoding RNA sequences were downloaded from RNACentral database (http://rnacentral.org)^64^. While all the other datasets contained protein sequences whose translation has been experimentally verified in literature, the downloaded RNAcentral dataset contained 9,386,637 nucleotide transcript sequences. We identified potential ORFs from these transcripts, using the following workflow. Each sequence was subjected to three-frame translation using the EMBOSS *transeq* program provided as a standalone utility by EMBL-EBI. From the output protein sequences, putative translated ORFs were obtained by identifying all possible subsequences (>30 residues in length) beginning with a methionine and ending at a STOP codon (EMBOSS *checktrans* program and Matlab scripts to parse the output text files). After removing redundant sequences from the extracted list, we obtained a unique set of 5,185,186 protein sequences, which we used as putative transcripts from the RNAcentral database for disorder prediction. Since the size of the RNACentral dataset far exceeded that of the four other novel datasets, we decided to keep the datasets segregated for future analysis.

### Disorder prediction

To predict protein disorder from sequence, we employed two disorder prediction algorithms, PONDR (http://www.pondr.com) and IUPred (https://iupred2a.elte.hu/). For PONDR, we used the VSL2 algorithm that was originally optimized and trained using both short and long protein sequences. Among the three IUPred-based algorithms, we performed separate predictions with IUPred ‘long disorder’ and IUPred ‘short disorder’. To predict possible structural elements from sequence, we used the Anchor program (http://anchor.enzim.hu)^42^. Matlab scripts were written to automate and batch process protein sequences for disorder and Anchor prediction, parse the output, and to perform statistical tests of enrichment (Fisher’s exact test, Chi-square test). All statistical tests were corrected for multiple hypothesis testing, using FDR values computed by the Benjamini–Hochberg method.

### Analysis for enrichment of post translational modifications on nORF peptides

To predict PTM sites from sequence, we used the ModPred stand-alone software^43^. For each sequence, we predicted amino acid sites for nine PTMs—phosphorylation, acetylation, methylation, sulfation, SUMOylation, ubiquitination, C-linked, O-linked, and N-linked glycosylation. To test if each of the datasets (NextProt, sORF, altORF, pseudogenes) have higher or lower predicted PTM site densities than expected at random, we generated an individual control dataset specific to that dataset as follows. We first obtained the average amino acid composition and length distribution for each dataset. We then fit a lognormal distribution to the sequence lengths. Individual control AA sequences were then generated with lengths drawn from the lognormal distribution, and probability of each amino acid chosen from the average amino acid compositions for the dataset. We generated such control sequences until the control dataset had twice the number of sequences as the original dataset. ModPred was then used to predict PTM sites in these control datasets for the same list of nine modifications. The number of predicted PTM sites in all datasets (test or control) were normalized to account for variable sequence length (per 100 residues).

### Mapping disease-associated mutations to nORFs

To investigate whether the novel protein regions could harbor disease-associated mutations, we mapped mutations from the COSMIC and HGMD databases to nORF peptides. Supplementary Fig. 2 shows examples of COSMIC or HGMD mutations mapped to all human sORFs, Denovogenes, and Pseudogenes demonstrating that these regions do indeed harbor mutations. We investigated whether the pathogenicity scores of these mutations, assessed as combined annotation-dependent depletion (CADD)^33^ and functional analysis through hidden Markov models (FATHMM)^65^ scores, had any correlation with disorder scores at the mutated region of the novel proteins (both amino-acid-specific disorder score, and average disorder score for a 7-aa window around the mutated residue). This analysis (Fig. 4d and Supplementary Fig. 12) did not reveal any correlation between low pathogenicity and higher disorder scores.

### B and T cells total RNA sequencing data acquisition

B and T cells extracted from the spleen of six male and six female C57BL/6J mice were FACS sorted to isolate resting B and naive CD4+ T cells. Total RNA was extracted from each of the 12 samples (three B-male, three B-female, three T-male, and three T-female) and sequenced using Illumina HiSeq 2500. This work was done in Ferguson-Smith lab at the Department of Genetics, University of Cambridge. Data can be accessed at NCBI GEO database, accession GSE94671.

Mice spleen tissues were obtained through a collaboration with Prof. Anne Ferguson-Smith’s lab at the Department of Genetics, University of Cambridge and the work was carried out in accordance with UK government Home Office licensing procedures (HO project license number: PC9886123) and approved by the University of Cambridge.

### Naive B and T cells separation for proteomics analysis

All steps were carried out fast, and the cells were maintained in ice- and ice-cold buffers. Spleen from six male and six female C57BL/6J mice, age 12 weeks (Supplementary Fig. 14), were collected in the cold 1× PBS (-Ca, -Mg) and gently crushed on a 40 µM Nylon cell strainer (Fisherbrand, 22363547) using Iscove’s modified Dulbecco’s medium (IMDM, Sigma I3390), 10% heat-inactivated FBS (Sigma F9665), 1% antibiotic + antimicotic (Sigma A5955) media, 1% l-glutamine (Sigma G7513)) to isolate splenocytes. Samples were centrifuged for 5 min at 400×*g* and the pellet was gently resuspended in 1x MojoSort buffer (Biolegend 480017). Cells were incubated with 2 ml of RBC lysis buffer (Biolegend, 420301) for 3 min, and afterwards with 8 ml IMDM-10. Cells were centrifuged for 5 min at 400 × *g*, resuspended in 1× MojoSort buffer to a final density 400 μl of buffer per 10^8^ total cells. We obtained on average 1.8 × 10e^8^ splenocytes per spleen.

To cells, 360 μl of antibody cocktail of biotin-conjugated monoclonal anti-mouse antibodies against CD8a, CD11b, CD11c, CD19, CD25, CD45R, CD49b, CD105, Ter-119, MHC class II, and TCRγ/δ from T cells isolation kit (Miltenyi,130-106-643) and cocktail of biotin-conjugated anti-mouse antibodies against CD43-Ly48, CD4-L3T4, and Ter-119 from B cells isolation kit (Miltenyi, 130-090-862) was added at a final concentration of 100 μl biotin antibody per 10e^8^ cells and were incubated for 5 min in the refrigerator. Cells bound with biotin-antibody, 1.08 ml of cold 1× MojoSort buffer and 720 μl of α-biotin microbeads were added (final 300 μl buffer and 200 μl microbeads per 10e^8^ cells) and incubated for an additional 10 min in the refrigerator. Cells were then diluted with 15 ml 1× mojo buffer and centrifuged for 5 min at 400×*g*. Pellets were resuspended in 1.44 ml 1× MojoSort buffer. Biotin-bound cells were depleted by passing through LD columns (Milteny, 130-042-901) in the magnetic field and flow-through was collected. Columns were washed twice with extra 1 ml of buffer. Pooled unlabeled cells (total volume ~3.44 ml per tube) represent the enriched T cells and B cells for subsequent FACS sorting.

Cells were counted as T Cells 2 × 10e^6^ cells per spleen, and B cells = 3 × 10e^7^ cells per spleen from 1.8 × 10e^8^ splenocytes. For sorting 100,000 cells per 100 μl were prepared to establish the % population of naive T and B cells obtained from antibody-mediated sorts. To cells, 1 μl antibody each of CD4-FITC, CD25-PE-Vio770 (PE-Cy7), CD44-APC, CD62L-PE was added for individual flow channels. Controls were prepared using 1 μl of Streptavidin-V450, and a negative control with no antibody in cells. A sample was prepared with all above five antibodies. For B cells, Streptavidin-V450 and CD45R (B220)-PE antibodies were used in similar manner. Antibodies and cells were incubated on ice for 20 min, and then 850 μl 1× Mojosort buffer was added. Cells were centrifuged at 2000 rpm, for 5 min and resuspended in 500 μl buffer. Cells were processed through flow cytometer and following outputs were measured. We obtained 95% CD4+ cells that were live, single cells and streptavidin negative. CD25 channel filter removed 0.3% cells further, resulting in about 94% cells. A CD62+ and CD44+ gates resulted in 73–80% (CD4+CD25−CD62+CD44−) cells. For B cells, >99% of population processed through FACS sorter were selected for naive B cells (CD45R (B220)+). Samples were sorted with above settings using 1 μl antibody per 1 × 10e^6^ cells, and the cells were pelleted for proteomics workflow.

### Extraction of B and T cells proteome

To extract total cellular proteome, cells were lysed in buffer (6 M urea, 2 M thiourea, 4% CHAPS, 5 mM magnesium acetate, 30 mM Tris pH 8.0), and 15 μg protein in 5× Laemmli buffer with 5% b-mercaptoethanol was loaded on Mini-PROTEAN^®^ TGX™ Precast Gels (BioRad). Gel lanes were cut into three sections for peptide extraction. Gel sections were cut into 1–2 mm cubes, washed with 50% acetonitrile and 100 mM ammonium bicarbonate solution until blue stain is washed. Gel pieces were treated with 100% acetonitrile, and then reduced with 10 mM DTT in 100 mM ammonium bicarbonate for reduction at 56 °C for 1 h, and alkylated with 55 mM iodoacetamide in 100 mM ammonium bicarbonate in dark for 45 min at room temperature. Gel pieces were washed with 100 mM ammonium bicarbonate, and then treated with 50% acetonitrile followed by 100% acetonitrile. Subsequently, gel pieces were treated with diluted trypsin (5 ng/µl) enzyme for overnight at 37 °C. Peptides were extracted, dried, and dissolved in 3% acetonitrile with 0.1% formic acid.

### Mass spectrometry analysis of the B and T cells proteome

All LC–MS/MS experiments were performed using a Dionex Ultimate 3000 RSLC nanoUPLC (Thermo Fisher Scientific Inc., Waltham, MA, USA) system and a Q Exactive Orbitrap mass spectrometer (Thermo Fisher Scientific Inc., Waltham, MA, USA). Separation of peptides was performed by reverse-phase chromatography at a flow rate of 300 nL/min and a Thermo Scientific reverse-phase nano Easy-spray column (Thermo Scientific PepMap C18, 2 μm particle size, 100 Å pore size, 75 μm i.d. × 50 cm length). Peptides were loaded onto a pre-column (Thermo Scientific PepMap 100 C18, 5 μm particle size, 100 Å pore size, 300 μm i.d. × 5 mm length) from the Ultimate 3000 autosampler with 0.1% formic acid for 3 min at a flow rate of 10 μl/min. After this period, the column valve was switched to allow elution of peptides from the pre-column onto the analytical column. Solvent A was water + 0.1% formic acid and solvent B was 80% acetonitrile, 20% water + 0.1% formic acid. The linear gradient employed was 2–40% B in 30 min.

The LC elutant was sprayed into the mass spectrometer by means of an Easy-Spray source (Thermo Fisher Scientific Inc.). All *m*/*z* values of eluting ions were measured in an Orbitrap mass analyzer, set at a resolution of 70,000 and was scanned between *m*/*z* 380 and 1500. Data-dependent scans (Top 20) were employed to automatically isolate and generate fragment ions by higher energy collisional dissociation (HCD, NCE:25%) in the HCD collision cell and measurement of the resulting fragment ions was performed in the Orbitrap analyzer, set at a resolution of 17,500. Singly charged ions and ions with unassigned charge states were excluded from being selected for MS/MS and a dynamic exclusion window of 20 s was employed.

### Assembly and analysis of B and T cells total RNA transcripts

Quality of sequenced reads was determined using FastQC (Supplementary Fig. 31). Primary assembly sequence and comprehensive gene annotation files for C57BL/6J, GENCODE release version M12, were used as the reference genome in our analysis. A genome index file to assist with read alignment was created using HISAT2-build, which extracts the exon and splice-site coordinates from the reference annotation. The paired-end sequenced reads where then aligned to the genome using HISAT2 run with default settings and the ‘–dta’ option to ensure that strand information is retained after alignment. The output SAM files were converted to BAM format and the aligned reads were sorted based on genomic coordinates using Picard SortSam. Aligned reads in the BAM files and the reference genome were used to assemble sample-specific transcripts using StringTie run with default settings and the ‘–fr’ option which assumes that reads were generated from a stranded library. The sensitivity and specificity of the StringTie output relative to the full reference annotation and to a subset of protein-coding transcripts extracted from the reference annotation (defined by the “transcript_type “protein_coding”” tag) was assessed using GffCompare run using default settings and –T option to suppress output of mapping files.

The StringTie merge function was used to create a list of non-redundant transcripts in B and T cells using the 12 sample-specific GTF files. This merged transcript GTF file along with the 12 BAM files containing aligned reads were used for a second StringTie run with parameters ‘-Be’ to calculate transcript FPKM values for each sample. Furthermore, we merged the information in the 12 CTAB files containing transcript FPKM values with the merged transcript file to create the final master transcriptomic file with ~164,000 transcripts. The master transcriptomic file was further analyzed as below.

We define unannotated transcripts in the master transcriptomic file as those without an ENSEMBL ID, and annotated/known transcripts as those which were assigned an ENSEMBL ID by StringTie. Transcripts identified in unlocalised contigs in chromosome 1 and chromosome 4 with the names ‘GL4XXXX’ and ‘JH5XXXX’, respectively, were removed. Additionally, the master transcript file was filtered to remove transcripts with ‘0’ FPKM values for all the 12 samples. This filtering gave us 109,441 transcripts. The remaining transcripts were categorized into four sub-groups: B-male, B-female, T-male, or T-female, based on whether at least one out of three samples corresponding to a sub-group had a non-zero FPKM value. Finally, the transcripts were categorized into B or T cell-specific transcriptomic datasets based on whether a transcript was present in at least one of the two sub-groups corresponding to a particular cell type. This resulted in 101,767 B cell-specific transcriptomic dataset and 99,552 T cell-specific transcriptomic dataset.

### Creation of B and T cell-specific nucleotide proteogenomic database

Transcript coordinates in the B and T cell-specific transcriptomic datasets were used to extract the corresponding nucleotide sequence from the reference genome using Bedtools Getfasta available in CGC. Bedtools Getfasta was run with default settings and with the name parameter = “True”, which ensures that the name column of the input BED file is used as the header for the output FASTA file. Furthermore, transcripts with length >100,000 nt were split into components of length <100,000 nt to facilitate downstream analysis using Mascot. The output FASTA files generated are our B and T cell-specific nucleotide proteogenomic database.

### Creation of sORF and altORF amino acid databases

Prabakaran Lab mouse sORF (mPLsORF) database was created using information curated from two sources: sORFs.org and SmProt. sORFs.org contains 1,127,154 mouse sORFs, which have been either computationally predicted or experimentally verified. We exported mouse sORFs from sORFs.org with default filters except for FLOSS classification, which was set to ‘GOOD’ and ‘EXTREME’. SmProt contains a list of computationally predicted small peptides identified in several species including mouse. We extracted 15,581 mouse sORFs from SmProt with filter parameters set to ‘ALL’. The downloaded information from SmProt did not provide chromosome information for sORFs. A macros code was, therefore, run on the SmProt website to specifically extract chromosome information for sORFs.

Both databases had several duplicate entries which were removed by filtering them based on their chromosome location and amino acid sequence. We assigned unique sORF ids of the format ‘mPLsORFXXXXXXXXXX’, where X denotes a number, to each sORF entry and created our sORF database with the following columns: Organism_name, Source_database, Chromosome_number, Start_coordinate, End_coordinate, Strand, and Amino_acid_sequence. There are still a few sORFs in our database with the same chromosome coordinates, but these duplicates were not removed because their corresponding amino acid sequences were different. Our final in house curated sORF database contains a total of 454,120 sORFs (Supplementary Fig. 18).

We downloaded mouse altORF coordinates from Roucou’s lab. Few altORFs had multiple chromosome numbers assigned to it. These were removed from our dataset to generate a final list of 2,15,320 altORFs (Supplementary Fig. 19) for downstream analysis.

### Proteogenomic workflow to investigate evidence of translation from sORF, altORF, and undefined novel ORFs in mouse B and T cells

Thermo mass spectrometry raw files were submitted to four databases search as described in Supplementary Fig. 15, utilizing Proteome Discoverer v2.1 and Mascot 2.6. Briefly, an average of 383,216 mass spectra were obtained from each sample. All mass spectra were initially searched independently against three amino acid databases—Uniprot database, sORF database, and altORF database and against the cRAP database of common contaminants. The spectra identification was performed with the following parameters: MS/MS mass tolerance was set to 0.8 Da, and the peptide mass tolerance set to 10 ppm. The enzyme specificity was set to trypsin, and two missed cleavages were tolerated. Carbamidomethylation of cysteine was set as a fixed modification, whilst variable modifications consisted of: oxidation of methionine, phosphorylation of serine, threonine, and tyrosine, and deamidation of asparagine and glutamine. High confidence peptide identifications were determined using Percolator node, where false discovery rate estimation (FDR) < 0.01 was used. A minimum of two high confidence peptides per protein was required for identification.

Out of 383,216 mass spectra, 165,418 mass spectra was mapped to Uniprot database; out of 383,216 mass spectra, 67,091 mass spectra was mapped to sORF database; out of 383,216 mass spectra, 32,269 mass spectra was mapped to altORF database. We then filtered the entries to remove ‘cRAP’, which are contaminants introduced during the experiment. Only those proteins with ‘Medium/High’ FDR values were retained. Finally, entries with no abundance values for all the four sub-groups were removed. After filtering for these parameters a total of 2030 known proteins, 1649 sORFs, and 9 altORFs were identified to be translated (Supplementary Fig. 16).

All unmatched mass spectra from each step were then exported, combined into a single mgf and duplicates were removed. B-cell-specific mgf file contained 111,227 spectra and T-cell-specific mgf contained 100,942 spectra. These files were then re-searched against B or T-cell-specific nucleotide proteogenomic databases in six frames. 18,545 mapped to B-cell-specific nucleotide proteogenomic database, 7384 spectra mapped T-cell-specific nucleotide proteogenomic database. Spectral matches were then filtered and validated by two independent approaches. The first validation was done with Mascot Decoy analysis in Mascot and a second independent validation was done with Percolator analysis in Proteome Discoverer (Thermo Scientific). Transcripts that were only identified by both the validation methodologies and with at least two peptides matching them were considered as translated. A total of 259 transcripts from both B and T cells nucleotide proteogenomic databases were identified to be translated with evidence of at least two peptides out of a total of 766 peptides mapping to them and these 259 regions were further analyzed as discussed below.

### Further processing proteogenomic results

Of the 259 transcripts identified to be translated, 176 transcripts were identified in B cells and 86 transcripts were identified in T cells. These transcript regions varied in length, with the largest being 1.4 million bases, and because two peptides were separated by vast distances in single transcripts it was difficult to identify any undefined ORFs in this region. So, we decided to investigate undefined ORFs based on individual peptides within these transcripts. To do this, we aligned the peptide and searched the genome up and downstream of the peptide until a stop or start codon was encountered. Out of 766 peptides 689 peptides were unique and 632 peptides aligned mouse genome with *e*-value < 0.01. Of these 632 peptides we could annotate 617 peptides into 835 undefined novel ORF regions. The genomic coordinates of these undefined ORFs (±500 bp up and downstream) were subsequently classified using Ensembl API (GET overlap/region) to identify neighboring genomic features (genes, transcript, exon, cds) in the mm10 genome. A small portion of these ORFs could not be classified due to the genomic features from Ensembl disagreeing at different levels.

### DE analysis of B and T cell transcripts

The 12 CTAB outputs from the StringTie run with parameters ‘-Be’, generated a list of sample-specific transcript FPKM values, which were used as inputs for DE analysis. Ballgown’s ‘stattest’ function performs a log_2_ transformation on the library-normalized FPKM values, fits the normalized values to a standard linear model and calculates *p* and *q* values for the transcripts. Here, transcripts with *q* values < 0.01 were called differentially expressed. Finally, the list of DE transcripts was filtered using Benjamin–Hochberg corrected *p*-values at a cutoff of 0.05.

### Structure prediction of sORFs, altORFs, and translated products from undefined novel ORFs

EVFold pipeline was setup according to instructions on the GitHub repository (https://github.com/debbiemarkslab/EVcouplings) on an Ubuntu AWS instance. This included the installation of the following software Hmmer suite 3.0, PLMC, CNS solve 1.2, HH-suite, Psipred, Maxcluster64. The database used was the recommended Uniref90 downloaded from https://www.uniprot.org/downloads.

### GO analysis of sORFs and altORFs against known proteins

Using interproscan-5.29-68 (downloaded from https://www.ebi.ac.uk/interpro/download.html), we annotated sORFs and altORFs for which we have translational evidence in at least one sample. In order to allow a fair comparison, known proteins downloaded from uniprot with translational evidence were also annotated with interproscan serving as a reference point. Non-automated annotation was not used as this information is not available for the majority of sORFs and altORFs. Proteins in the reference genome were referenced using uniprot accession IDs and the genes mapped to these IDs were obtained using the uniprot online mapping service (https://www.uniprot.org/mapping/). Analysis was performed on presence or absence of GO term annotation rather than the number of times the gene or protein might have been annotated with the same GO term.

Chi-squared tests were then performed with expected values based on the known protein proportions. The Bioconductor qvalue package was used to calculate *q*-values to be used for FDR correction. Cutoffs of *q* < 0.01 and *p* < 0.01 were used to select significantly enriched or depleted GO terms in sORFs. This analysis was not carried out for altORFs due to the low number of annotated GO terms. The significant GO terms were then clustered using the Bioconductor GOSim package using default settings of getTermSim.

### Mapping and visualization of disease-associated mutations in sORFs and undefined ORFs from the mouse study

We developed computational strategies using bedtools intersect to map mutations from the HGMD and COSMIC database on to protein and protein-like products from the noncoding regions. For that we had to first identify human homologous sequences. Briefly, LiftOver and NCBI tblastn, with attributes -word_size 2, was used to map mouse sORFs to the human genome, build hg38. tblasn results further filtered using the following tblastn parameters constraints pident>80 & ppos>80 & ((mismatch*100)/qend) <10 & ((qstart*100)/qend) <25 & qcovs >80 & gaps ≤ 2 & gapopen ≤ 1).

LiftOver and tblastn mapped 4325 mouse sORFs to 1339 and 3429 regions for build hg38, respectively. Only 1339 regions for hg38 were mapped commonly from both LiftOver and tblastn. GRCh38-mapped coordinates of the translated nORFs were scanned against Cosmic and HGMD variant databases using bedtools intersect without strand specification. Mapped mutations from each region were then compared to the coding sequence of each sORF to determine potential changes to amino acid sequence using python script. For the sORFs with predicted structures available, the mutations were mapped onto the PDB file and visualized with Pymol as red colored residues.

### DE analysis of nORF transcripts using Xena’s TCGA-TARGET-GTEx dataset

The expression of transcripts translating low-noise nORFs (identified from 353 datasets corresponding to 11 cell types downloaded from RPFdb), was investigated in 19 human cancers with the objective to identify probable cancer markers. The 225,273 low-noise, unique nORFs mapped to 96,828 unique transcripts, of which 43,653 are not of the transcript_type as ‘protein_coding’ and status as ‘known’ according to gencode v23. This list of 43,653 transcripts was further filtered to retain only the 110 non-protein_coding transcripts which translates unique low-noise nORF in all the 11 cell types.

To compare the expression of nORF transcript in a particular cancer to its expression in the corresponding healthy tissue, isoform level (RSEM-TPM) abundances, cataloged at UCSC Xena (cohort: TCGA TARGET GTEx, version 2016-09-02), was used^27^. These datasets are generated by uniform processing of RNAseq raw reads from TCGA’s tumor and matched normal samples and GTEx’s healthy tissue samples using a recently published TOIL pipeline. The TCGA cancers and their corresponding healthy tissues from GTEx, along with the number of samples in each case, analyzed in this study is given in Supplementary Table 6.

Each transcript in the whole transcriptome was annotated as ‘expressed’ in that study (GTEx normal and TCGA tumor) if it had non-zero expression in more than 25% of the samples. The expressed transcripts were further analyzed for DE between GTEx vs. TCGA tumor, using Welch’s *t*-test with BH correction.

### Mouse sORFs structure prediction and mutation mapping

Human ortholog transcript of one mouse sORF that is translated in mouse B and T cells was identified, its structure predicted and inhibitors were screened against it. The details are as follows.

Human ortholog of mPLsORF0000447155 sORF was identified using tblastn+liftover (*e*-value: 4.00E−19, length: 90, pident: 91.11, mismatch: 8), and it maps to a genomic location of a human transcript ENST00000427352.1: chr5:115553723–115553992:- (GRCh37). This transcript ‘ENST00000427352.1’, annotated is ‘processed_pseudogene’, is expressed only in the tumor samples of stomach adenocarcinoma, esophageal carcinoma, acute myeloid leukemia, and is expressed only in the normal samples of testicular germ cell tumor. We call a transcript expressed in particular condition if it has non-zero expression in more than 10% of the samples. We mapped two cosmic noncoding mutations to this transcript. Structure of the human sORF was predicted using Evfold pipeline with the following parameters: Bit score = 0.2, seqlen = 90, N_eff/L = 3.85, number of effective sequences = 342, number of sequences in alignment (num_seqs) = 1063, perc_cov = 0.944. Figure 6b shows the structure along with the mutations mapped.

### Inhibitor screens for the two sORFs identified to be disrupted in cancers

Structure predicted from ENST00000427352.1 (human ortholog of mPLsORF0000447155 sORF) was chosen for drug screening study. Briefly, structure-based virtual screening analysis was performed using Virtual screening workflow of Schrödinger software suite (http://gohom.win/ManualHom/Schrodinger/Schrodinger_2015–2_docs/vsw/vsw_user_manual.pdf). First in the protein preparation step, the structure was minimized using protein preparation wizard in maestro 12.1 (Schrodinger) applying force field OPLS3 with default parameters. Next, the active sites were predicted using SiteMap (Schrodinger) and CastP. The grid was generated at all the active site residues of the topmost scoring pocket identified by the two tools.

mPLsORF0000447155: MPKRKAEGDAKGDKTKVKDEPQRRSARLSAKPAPPKPEPKPKKAPAKKGEKVPKGKKGKADAGKDANNPAENGDAKTDQAQKAEGAGDAK.

Peptide sequence of the product translated from ENST00000427352.1:MPKRKAEGDAKGDKAKVKDEPQRRSARLSAKPASPKPEPRPKKAPAKKGEKVPKGRKGKADAGKEGNNPAENGDVKTDQAQKAEGAGGAK.

The predicted active Site Residues used in docking are given in Supplementary Table 6 and the accompanying associated figure is Supplementary Fig. 32.

The virtual screening involved the following three stages: 1. high throughput virtual screening (HTVS), 2. standard precision (SP), and 3. extra precision (XP) docking. The small molecules of the following three libraries obtained from Asinex library was used for docking: Immuno oncology (11,346) compounds (http://asinex.com/wp-content/uploads/2017/01/2016–11-Asinex-Immuno-Oncology-11346.zip), targeted oncology (6728) compounds (http://asinex.com/wp-content/uploads/2016/11/2016–11-Asinex-Targeted-Oncology-6728.zip), and signal pathway inhibitors (5923) (http://asinex.com/wp-content/uploads/2017/01/2016–11-Asinex-Signal-Pathway-Inhibitors-5923.zip). The 2D SDF format of all the compounds structures in these libraries were converted into 3D format using Schrodinger’s LigPrep module with OPLS3 Force Field. A three-step docking methodology was used—Glide HTVS, SP, and XP. Listed below are the details of the predicted best hit compounds searched from the three asinex libraries.

Docking scores for the top Immuno-oncology library compounds, targeted-oncology library compounds, signaling pathway inhibitors are given in Supplementary Tables 7–9, respectively, and their associated figures are Supplementary Figs. 33–35, respectively.

MM-GBSA-binding energies, which estimates relative binding affinities for the few best hit immuno-oncology compounds, targeted-oncology compounds, and signaling pathway inhibitors are given in Supplementary Tables 10–12, respectively.

### Reporting summary

Further information on research design is available in the Nature Research Reporting βSummary linked to this article.

## Supplementary information

Supplementary Information

Reporting Summary

## Data Availability

Almost all processed data is in the main text or in the supplementary materials. Transcriptomic data can be obtained from GEO accession GSE94671 and GSE94676. The mass spectrometry proteomics data are deposited to the ProteomeXchange Consortium via the PRIDE^66^ partner repository with the dataset identifier PXD022099. The results shown are in part based upon data generated by the TCGA Research Network: https://www.cancer.gov/tcga.

## Code availability

All codes for this work can be obtained from https://github.com/PrabakaranGroup/norfs-cancer-biological-functions.

## References

[1] Vitting-Seerup K, Sandelin A (2017). The landscape of isoform switches in human cancers. Mol. Cancer Res..

[2] Hu X, Sood AK, Dang CV, Zhang L (2017). The role of long noncoding RNAs in cancer: the dark matter matters. Curr. Opin. Genet. Dev..

[3] Rheinbay E (2020). Analyses of non-coding somatic drivers in 2,658 cancer whole genomes. Nature.

[4] Wang J (2019). ncRNA-encoded peptides or proteins and cancer. Mol. Ther..

[5] Brunet, M. A. et al. OpenProt: a more comprehensive guide to explore eukaryotic coding potential and proteomes. *Nucleic Acids Res*. 10.1093/nar/gky936 (2018). 10.1093/nar/gky936 PMC6323990 30299502

[6] Plaza, S., Menschaert, G. & Payre, F. In search of lost small peptides. *Annu. Rev. Cell Dev. Biol*. 10.1146/annurev-cellbio-100616-060516 (2017). 10.1146/annurev-cellbio-100616-060516 28759257

[7] Clamp M (2007). Distinguishing protein-coding and noncoding genes in the human genome. Proc. Natl Acad. Sci. USA.

[8] Prabakaran S (2014). Quantitative profiling of peptides from RNAs classified as noncoding. Nat. Commun..

[9] Ruiz-Orera J, Verdaguer-Grau P, Villanueva-Cañas JL, Messeguer X, Albà MM (2018). Translation of neutrally evolving peptides provides a basis for de novo gene evolution. Nat. Ecol. Evol..

[10] Zhu Y (2018). Discovery of coding regions in the human genome by integrated proteogenomics analysis workflow. Nat. Commun..

[11] Olexiouk, V. & Menschaert, G. Using the sORFs.Org Database. *Current protocols in bioinformatics*, **65**, e68 (2019). 10.1002/cpbi.68 30485709

[12] Vanderperre B (2013). Direct detection of alternative open reading frames translation products in human significantly expands the proteome. PLoS ONE.

[13] Mc Lysaght A, Hurst LD (2016). Open questions in the study of denovo genes: what, how and why. Nat. Rev. Genet..

[14] Tautz D, Domazet-Lošo T (2011). The evolutionary origin of orphan genes. Nat. Rev. Genet..

[15] Xu J, Zhang J (2016). Are human translated pseudogenes functional?. Mol. Biol. Evol..

[16] Yeasmin F, Yada T, Akimitsu N (2018). Micropeptides encoded in transcripts previously identified as long noncoding RNAS: a new chapter in transcriptomics and proteomics. Front. Genet..

[17] Smith JE (2014). Translation of small open reading frames within unannotated RNA transcripts in *Saccharomyces cerevisiae*. Cell Rep..

[18] Ingolia NT (2014). Ribosome profiling reveals pervasive translation outside of annotated protein-coding genes. Cell Rep..

[19] Stein CS (2018). Mitoregulin: a lncRNA-encoded microprotein that supports mitochondrial supercomplexes and respiratory efficiency. Cell Rep..

[20] Cohen SM (2014). Everything old is new again: (linc)RNAs make proteins!. EMBO J..

[21] Steward CA (2017). Genome annotation for clinical genomic diagnostics: strengths and weaknesses. Genome Med..

[22] Leslie, M. New universe of miniproteins is upending cell biology and genetics. *Science.*10.1126/science.aaz8818 (2019).

[23] Merino-Valverde, I., Greco, E. & Abad, M. The microproteome of cancer: from invisibility to relevance. *Exp. Cell Res*. **392**, 111997 (2020). 10.1016/j.yexcr.2020.111997 32302626

[24] Hanahan D, Weinberg RA (2011). Hallmarks of cancer: the next generation. Cell.

[25] Huang J-Z (2017). A peptide encoded by a putative lncRNA HOXB-AS3 suppresses colon cancer growth. Mol. Cell.

[26] Zhang M (2018). A peptide encoded by circular form of LINC-PINT suppresses oncogenic transcriptional elongation in glioblastoma. Nat. Commun..

[27] Vivian J (2017). Toil enables reproducible, open source, big biomedical data analyses. Nat. Biotechnol..

[28] Wang H (2019). RPFdb v2.0: an updated database for genome-wide information of translated mRNA generated from ribosome profiling. Nucleic Acids Res..

[29] Carvunis A-R (2012). Proto-genes and de novo gene birth. Nature.

[30] Neme R, Tautz D (2013). Phylogenetic patterns of emergence of new genes support a model of frequent de novo evolution. BMC Genomics.

[31] Kaessmann H (2010). Origins, evolution, and phenotypic impact of new genes. Genome Res..

[32] Chen X, Zhang J (2016). The genomic landscape of position effects on protein expression level and noise in yeast. Cell Syst..

[33] Rentzsch, P., Witten, D., Cooper, G. M., Shendure, J. & Kircher, M. CADD: predicting the deleteriousness of variants throughout the human genome. *Nucleic Acids Res*. 10.1093/nar/gky1016 (2018). 10.1093/nar/gky1016 PMC6323892 30371827

[34] Hao, Y. et al. SmProt: a database of small proteins encoded by annotated coding and non-coding RNA loci. *Brief Bioinform*. 10.1093/bib/bbx005 (2017). 10.1093/bib/bbx005 28137767

[35] Olexiouk V, Van Criekinge W, Menschaert G (2018). An update on sORFs.org: a repository of small ORFs identified by ribosome profiling. Nucleic Acids Res..

[36] Pertea G, Pertea M (2020). GFF utilities: GffRead and GffCompare. F1000Res..

[37] Frankish A (2019). GENCODE reference annotation for the human and mouse genomes. Nucleic Acids Res..

[38] Robinson MD, Oshlack A (2010). A scaling normalization method for differential expression analysis of RNA-seq data. Genome Biol..

[39] Robinson MD, McCarthy DJ, Smyth G (2010). K. edgeR: a Bioconductor package for differential expression analysis of digital gene expression data. Bioinformatics.

[40] The RNAcentral Consortium. (2019). RNAcentral: a hub of information for non-coding RNA sequences. Nucleic Acids Res..

[41] Rocklin GJ (2017). Global analysis of protein folding using massively parallel design, synthesis, and testing. Science.

[42] Dosztányi Z, Mészáros B, Simon I (2009). ANCHOR: web server for predicting protein binding regions in disordered proteins. Bioinformatics.

[43] Pejaver V (2014). The structural and functional signatures of proteins that undergo multiple events of post-translational modification. Protein Sci..

[44] Marks DS, Hopf TA, Sander C (2012). Protein structure prediction from sequence variation. Nat. Biotechnol..

[45] Liu Y, Beyer A, Aebersold R (2016). On the dependency of cellular protein levels on mRNA abundance. Cell.

[46] Nusinow DP (2020). Quantitative proteomics of the Cancer Cell Line Encyclopedia. Cell.

[47] Ghandi, M. et al. Next-generation characterization of the Cancer Cell Line Encyclopedia. *Nature*. 10.1038/s41586-019-1186-3 (2019). 10.1038/s41586-019-1186-3 PMC6697103 31068700

[48] Law GL, Raney A, Heusner C, Morris DR (2001). Polyamine regulation of ribosome pausing at the upstream open reading frame of S-adenosylmethionine decarboxylase. J. Biol. Chem..

[49] Matsumoto A (2017). mTORC1 and muscle regeneration are regulated by the LINC00961-encoded SPAR polypeptide. Nature.

[50] Nelson BR (2016). A peptide encoded by a transcript annotated as long noncoding RNA enhances SERCA activity in muscle. Science.

[51] Pueyo JI (2016). Hemotin, a regulator of phagocytosis encoded by a small ORF and conserved across Metazoans. PLoS Biol..

[52] Slavoff, S. A., Heo, J., Budnik, B. A., Hanakahi, L. A. & Saghatelian, A. A human short ORF-encoded peptide that stimulates DNA end joining. *J. Biol. Chem*. 10.1074/jbc.C113.533968 (2014). 10.1074/jbc.C113.533968 PMC4036235 24610814

[53] Ji Z (2018). Rfoot: transcriptome-scale identification of RNA–protein complexes from ribosome profiling data. Curr. Protoc. Mol. Biol..

[54] Kim KH, Son JM, Benayoun BA, Lee C (2018). The Mitochondrial-Encoded Peptide MOTS-c Translocates to the nucleus to regulate nuclear gene expression in response to metabolic stress. Cell Metab..

[55] Ji, Z., Song, R., Regev, A. & Struhl, K. Many lncRNAs, 5’UTRs, and pseudogenes are translated and some are likely to express functional proteins. *Elife* **4**, e08890 (2015). 10.7554/eLife.08890 PMC4739776 26687005

[56] Rainer J, Gatto L, Weichenberger C (2019). X. ensembldb: an R package to create and use Ensembl-based annotation resources. Bioinformatics.

[57] Rau A, Gallopin M, Celeux G, Jaffrézic F (2013). Data-based filtering for replicated high-throughput transcriptome sequencing experiments. Bioinformatics.

[58] Grambsch, P. M. & Therneau, T. M. Modeling survival data: extending the Cox model. *Stat. Biol. Health* (2000).

[59] Kassambara, A., Kosinski, M. & Biecek, P. survminer: drawing survival curves using’ggplot 2’. R package version 0.4.6 (2019).

[60] Quinlan AR, Hall IM (2010). BEDTools: a flexible suite of utilities for comparing genomic features. Bioinformatics.

[61] Madeira F (2019). The EMBL-EBI search and sequence analysis tools APIs in 2019. Nucleic Acids Res..

[62] Jones P (2014). InterProScan 5: genome-scale protein function classification. Bioinformatics.

[63] Gaudet P (2015). The neXtProt knowledgebase on human proteins: current status. Nucleic Acids Res..

[64] The RNAcentral Constortium. RNAcentral: a hub of information for non-coding RNA sequences. *Nucleic Acids Res*. 10.1093/nar/gky1034 (2018).

[65] Shihab HA (2013). Predicting the functional, molecular, and phenotypic consequences of amino acid substitutions using hidden Markov models. Hum. Mutat..

[66] Perez-Riverol Y (2019). The PRIDE database and related tools and resources in 2019: improving support for quantification data. Nucleic Acids Res..

