# Supplementary material for: Pan-cancer analysis of transcripts encoding novel open-reading frames (nORFs) and their potential biological functions
Source: NPJ Genom Med. 2021 Jan 25;6:4. doi: 10.1038/s41525-020-00167-4 (PMC7835362; doi:10.1038/s41525-020-00167-4)
Supplement: Supplementary file 1 — Supplementary Information [file 41525_2020_167_MOESM1_ESM.pdf]

Supplementary Figures

Pan-cancer analysis of transcripts encoding novel open reading frames (nORFs) and their potential biological functions

Authors: Chaitanya Erady[1†], Adam Boxall[1†], Shraddha Puntambekar[2†], N. Suhas Jagannathan[3†], Ruchi Chauhan[1†], David Chong[1], Narendra Meena[1], Apurv Kulkarni[2], Bhagyasrhi Kasabe[2], Kethaki Prathivadi Bhayankaram[1], Yagnesh Umrania[4], Adam Andreani[1], Jean Nel[1], Matthew T. Wayland[5], Cristina Pina[6], Kathryn S. Lilley[4], & Sudhakaran Prabakaran[1*]

Supplementary Figure 1

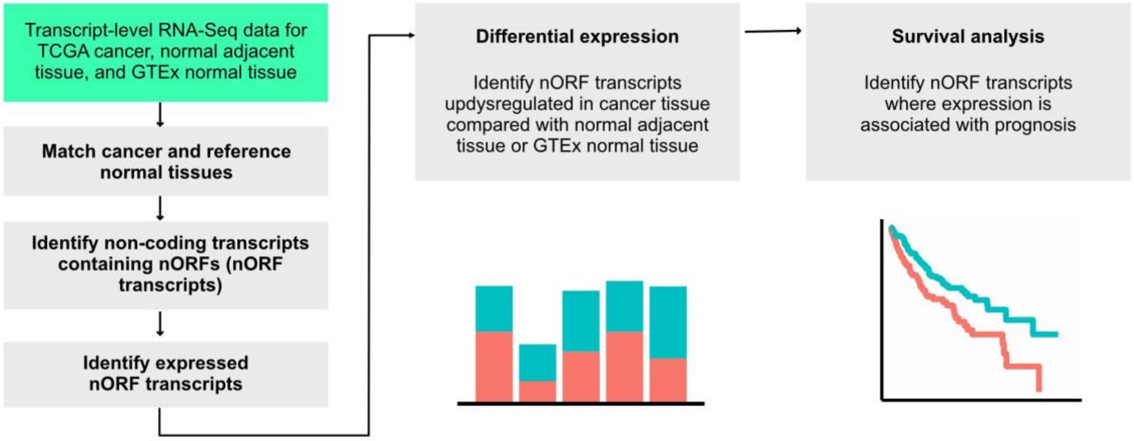

Supplementary Figure 1. Scope of the analysis. We obtain RNA-Seq transcript-level expected counts for samples in TCGA and GTEx, match normal and cancer tissues, identify expressed nORF transcripts and perform differential expression and survival analysis.

Supplementary Figure 2a

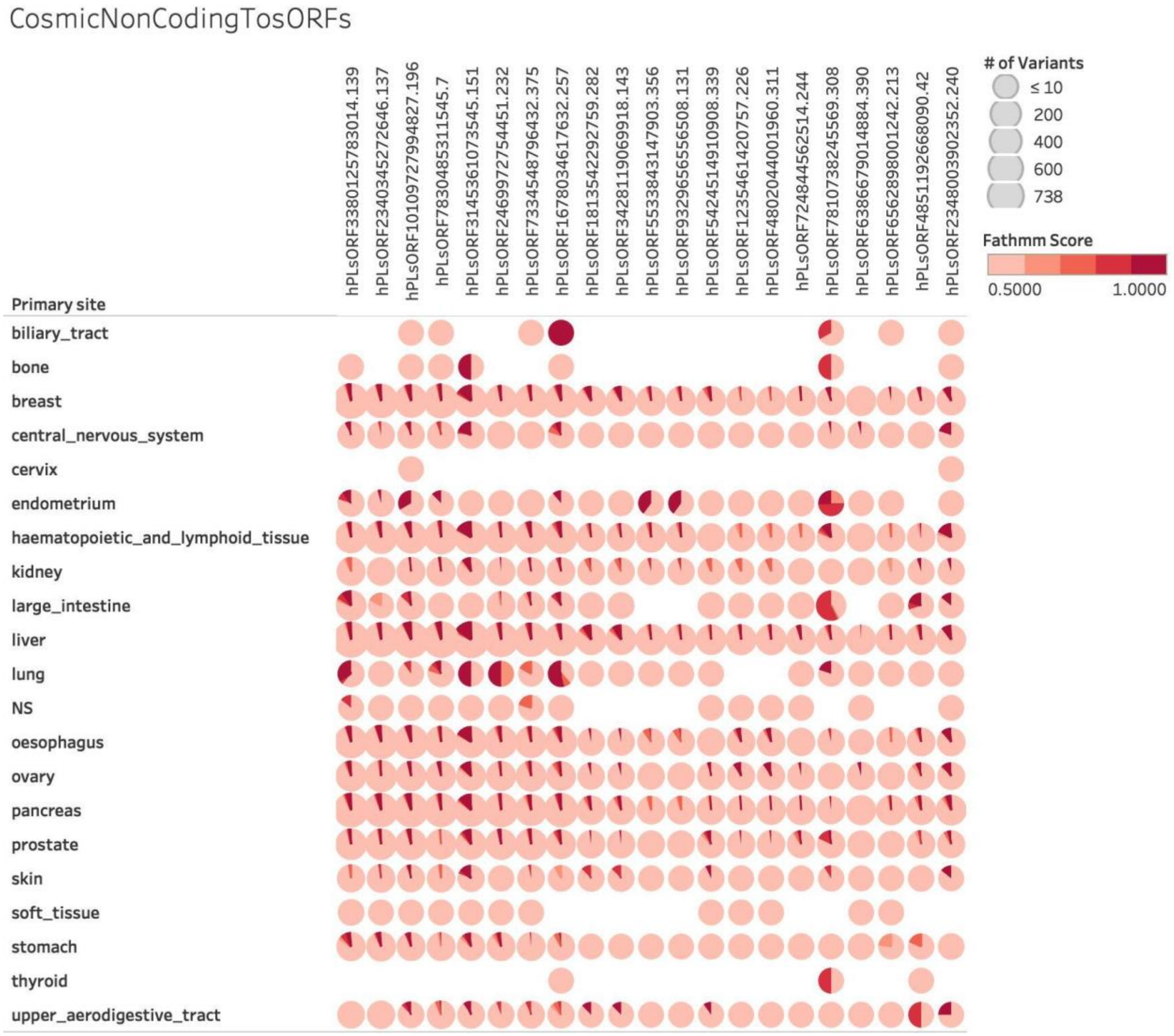

Supplementary Figure 2b

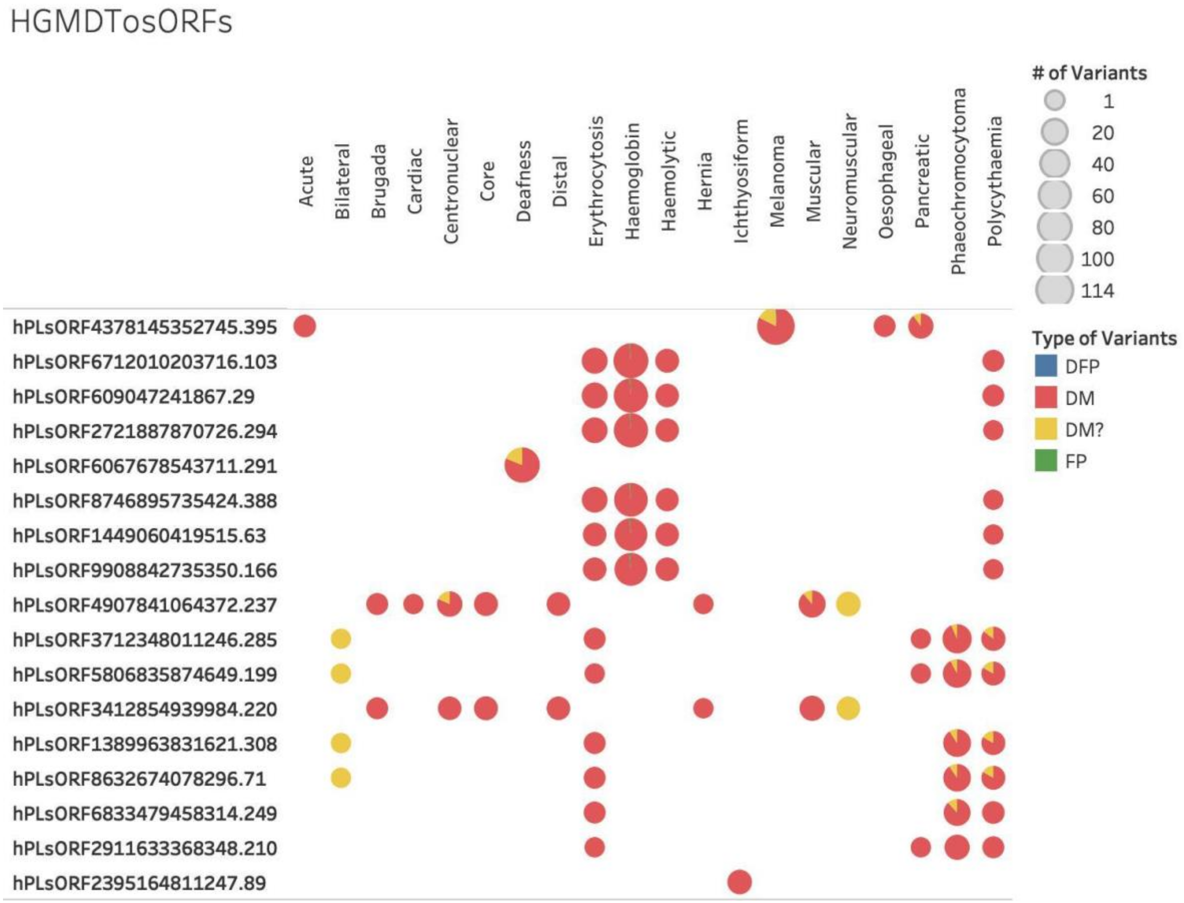

Supplementary Figure 2c

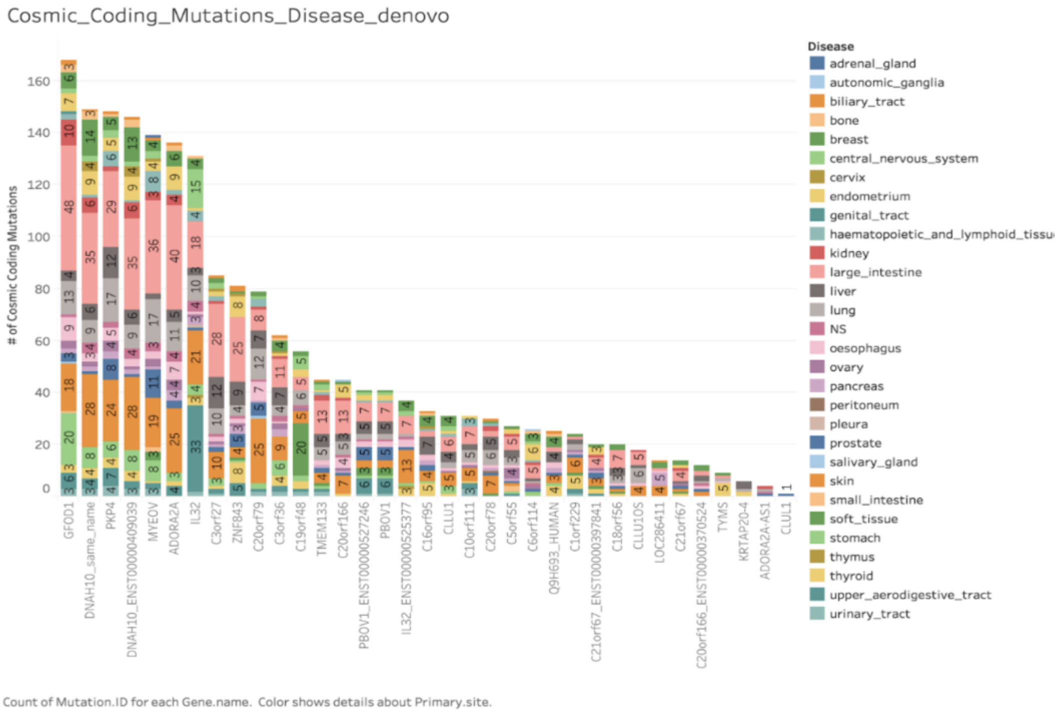

Supplementary Figure 2d

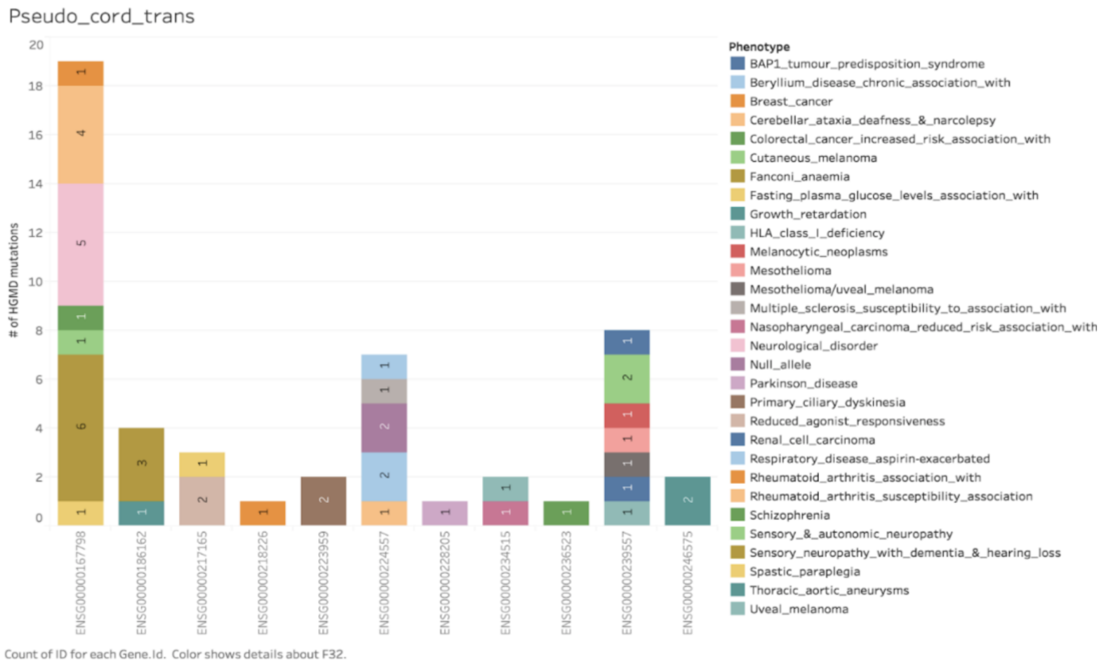

Supplementary Figure 2. Known mutations in proteins encoded by the nORFs. Mutations from COSMIC and HGMD databases were mapped to both entire nORFs genomic and specifically to novel protein amino acid sequence coordinates and represented as described below. **a.** Noncoding mutations from COSMIC were mapped to all sORFs genomic coordinates and only the top 21 sORFs with the highest number of mutations are represented here. Sizes of the pie charts indicate the number of variants that mapped to sORFs for each disease and the color indicates the pathogenicity (FATHMM, higher the score, more the pathogenicity). **b.** Mutations from the HGMD database were mapped to sORFs and only the top 17 mutations are represented here. Sizes of the pie charts indicate the number of variants that mapped to sORFs for each disease and the color indicates the type of HGMD variants. DM, disease-causing mutations; DM? denoting a probable pathological mutation; DP, disease-associated polymorphisms; FP, functional polymorphisms. **c.** Coding mutations from COSMIC are mapped to all Denovogenes and are represented. **d.** Mutations from HGMD are mapped to pseudogenes that are known to be translated.

Supplementary Figure 3

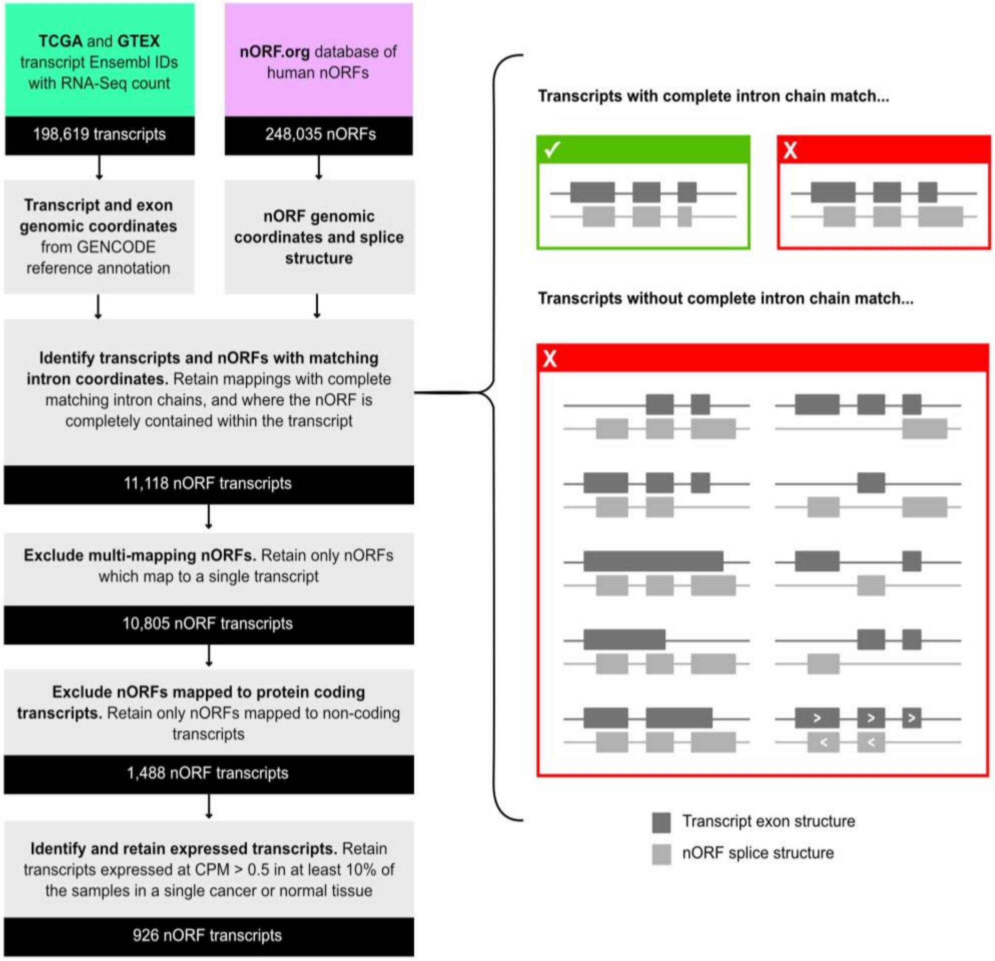

Supplementary Figure 3. Identifying expressed transcripts encoding novel open reading frames. Computational pipeline used to identify transcripts containing novel open reading frames, and the types of mapping between nORF and transcript genomic coordinates accepted and rejected in this pipeline.

Supplementary Figure 4

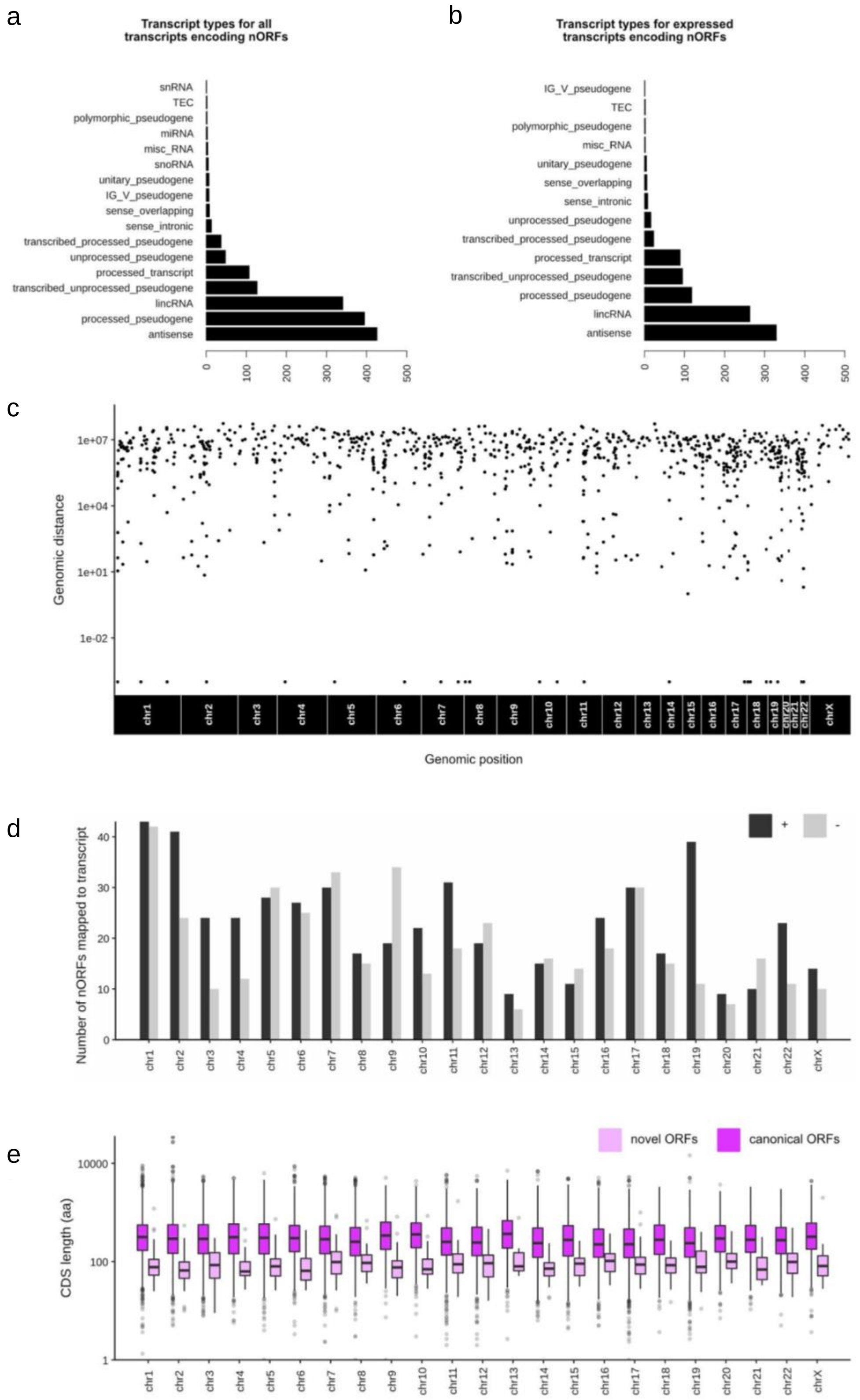

Supplementary Figure 4. Identifying expressed transcripts encoding novel open reading frames. Frequency of canonical transcript Ensembl biotypes for noncoding transcripts containing nORFs, for **a**. all nORF transcripts and **b**. expressed nORF transcripts considered in this study. **c.** Rainfall graph showing the genomic distribution of expressed nORF transcripts, measured in nucleotides from the nORF start site, with a pseudo-count of 0.0001. **d.** Frequency of expressed nORF transcripts by chromosome and strand. **e.** Distribution of ORF length for novel and canonical ORFs, by chromosome.

Supplementary Figure 5

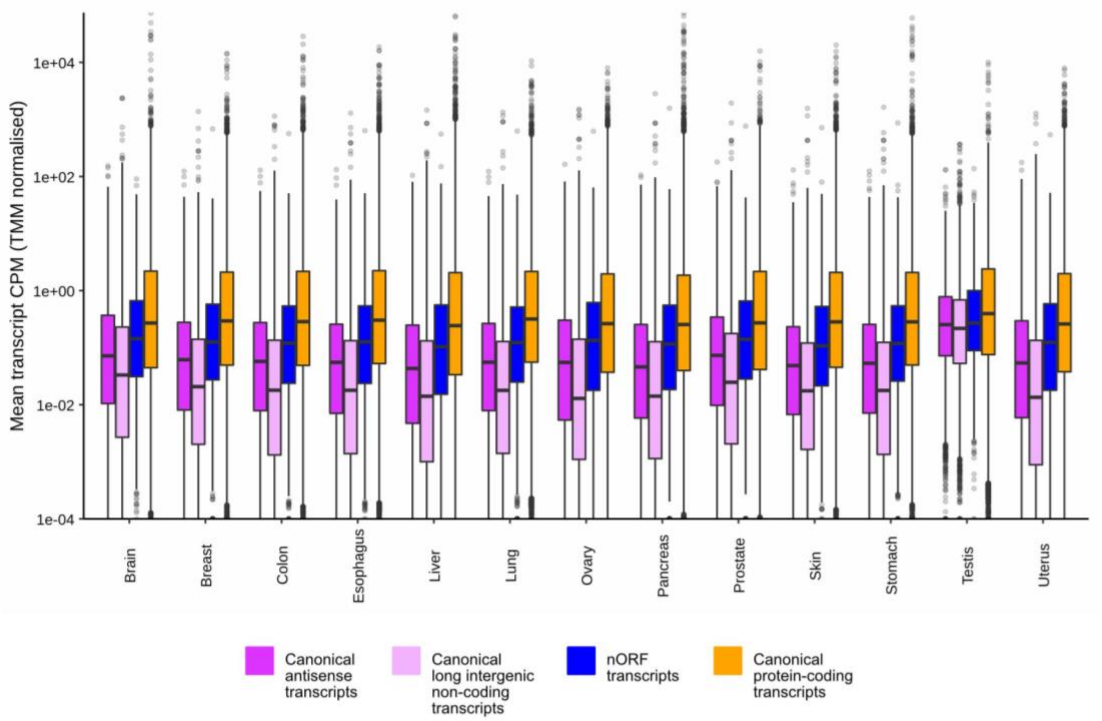

Supplementary Figure 5. Expression of nORF transcripts in normal tissues. Mean CPM value (TMM normalized) for nORF transcripts by tissue, log transformed with a pseudo-count of 0.0001. Mean expression of nORF transcripts compared with protein coding, long intergenic non-coding and antisense transcripts across GTEx normal tissues.

Supplementary Figure 6a

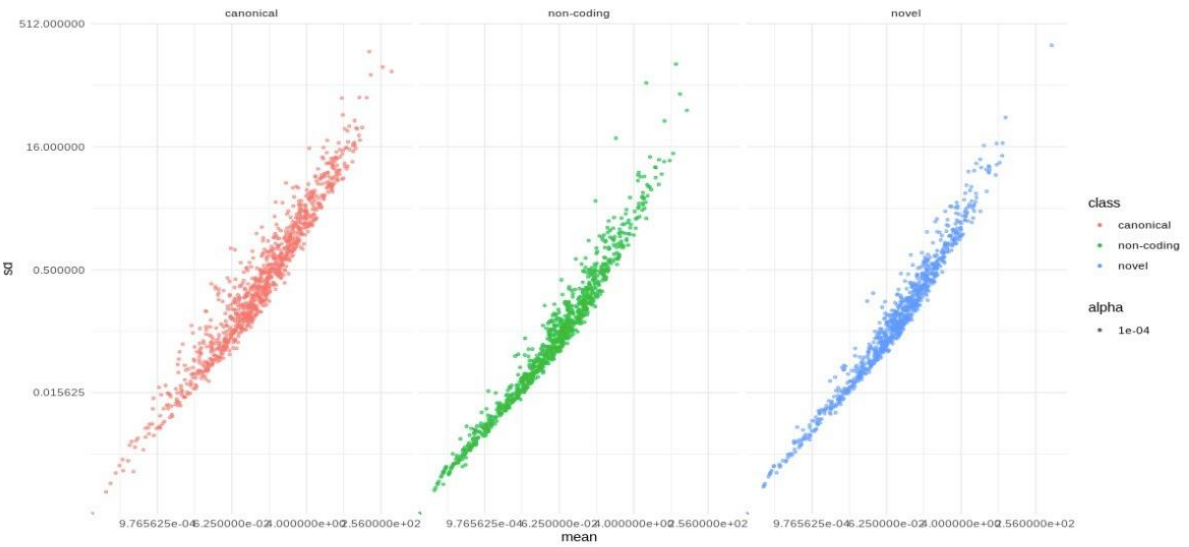

Supplementary Figure 6b

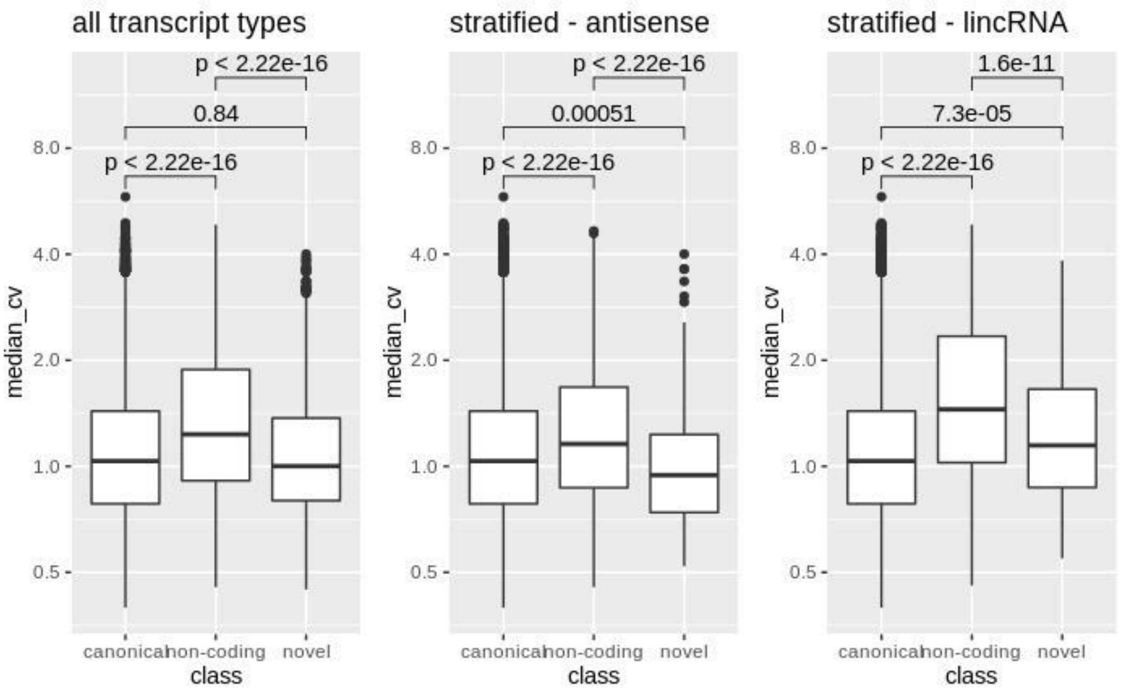

Supplementary Figure 6. Transcript expression across GTEx tissues. Means and standard deviations for TMM normalized expression counts (CPM) are calculated tissue-wise across all tissues included from the GTEx dataset and a median coefficient of variation (CV) is calculated from tissue-wise variations. Transcripts are classified as canonical protein coding, non-coding or novel based on the

workflow presented in Supplementary Figure 3, Supplementary Figure 4a and as detailed in Methods. **a.** Tissue-wise mean and standard deviation for lung tissue - a random sample of 1000 transcripts from each class is shown to limit overplotting. **b.** CV distributions for each transcript class are compared using a non-parametric Wilcoxon statistical test, and p-values are displayed. Transcript subsets for ‘non-coding’ and ‘novel’ transcripts are produced by stratifying by transcript type, and CV comparisons for antisense and lincRNA transcripts are performed in isolation.

Supplementary Figure 7

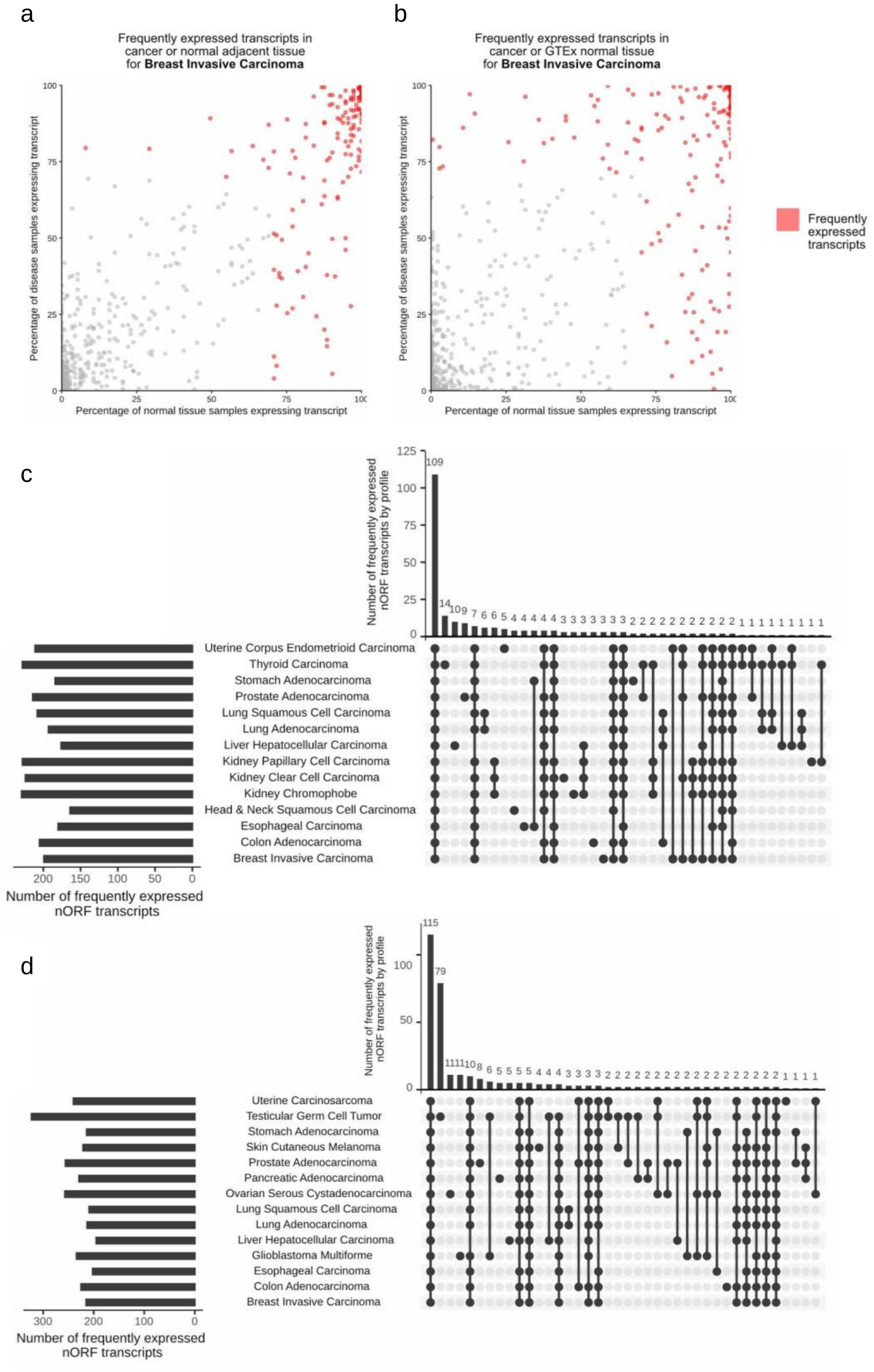

Supplementary Figure 7. Frequently expressed nORF transcripts across cancer and normal reference samples. Percentage of samples exhibiting transcript expression greater than 0.5 CPM for each expressed nORF transcript. Representative plot shown for breast invasive carcinoma tissue compared with **a.** normal adjacent tissue **b.** GTEx normal tissue. nORF transcripts identified as frequently expressed are highlighted. Profiles of frequently expressed nORF transcripts across cancer types, considering **c.** cancer and normal adjacent tissue and **d.** cancer and GTEx normal tissue.

Supplementary Figure 8a

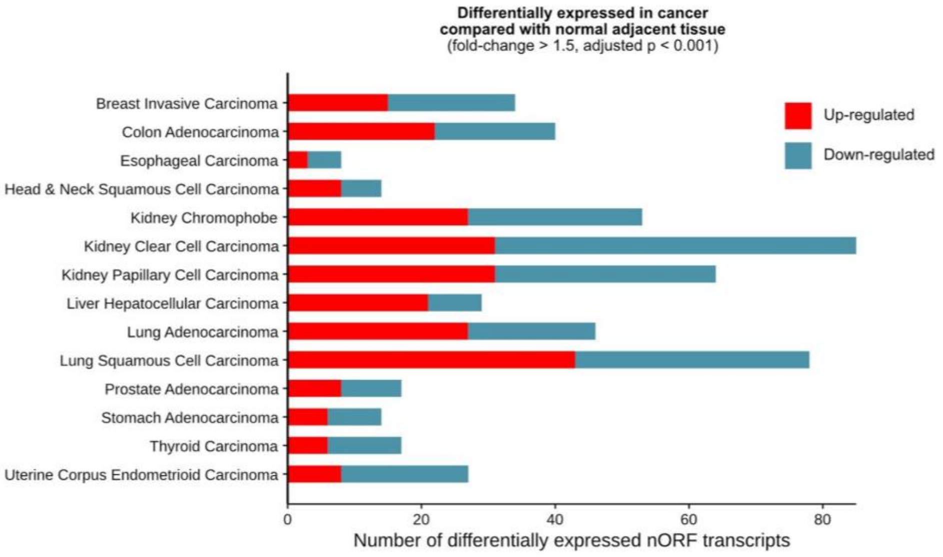

Supplementary Figure 8B

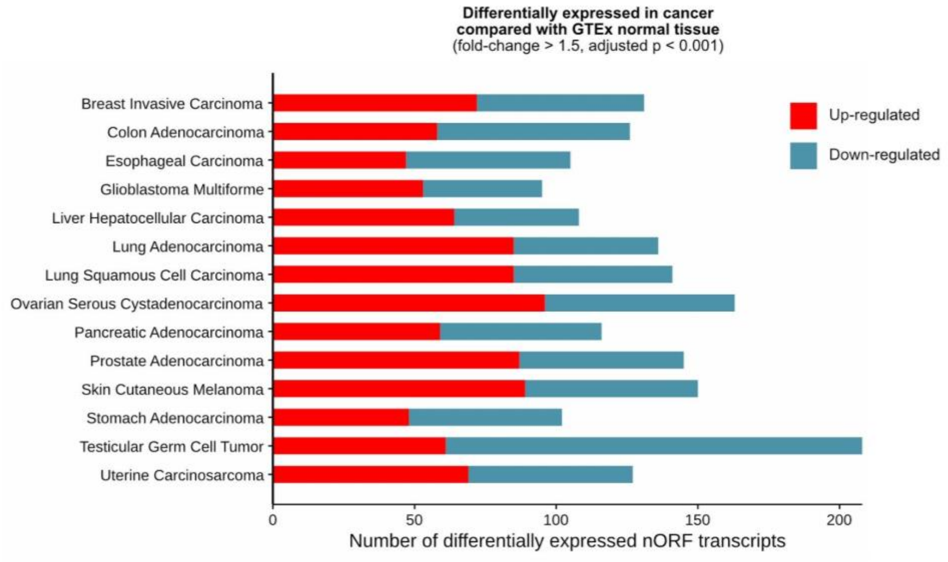

Supplementary Figure 8c

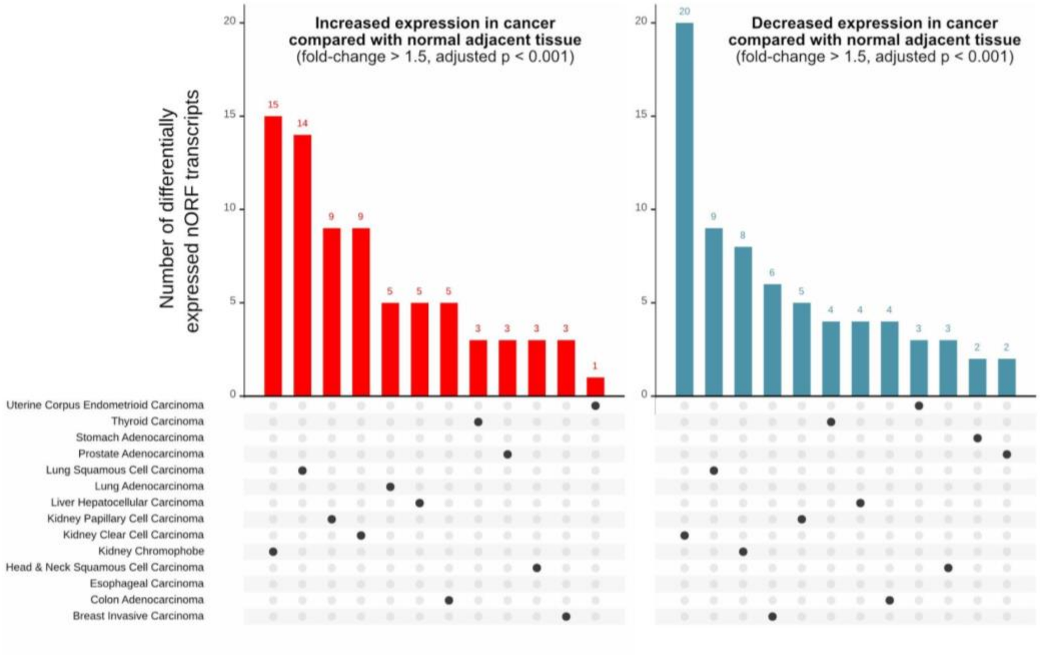

Supplementary Figure 8d

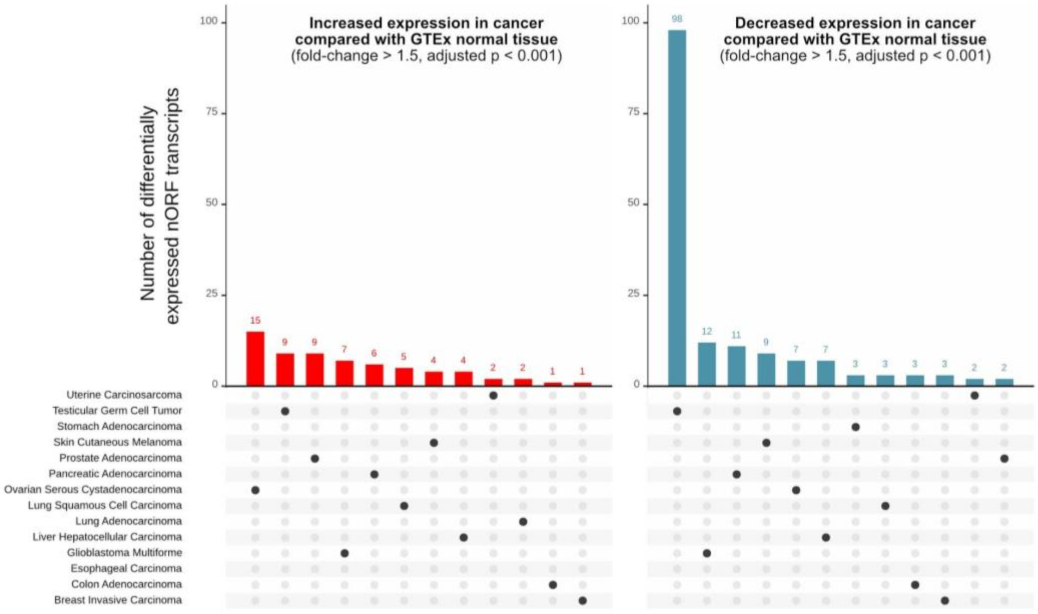

Supplementary Figure 8e

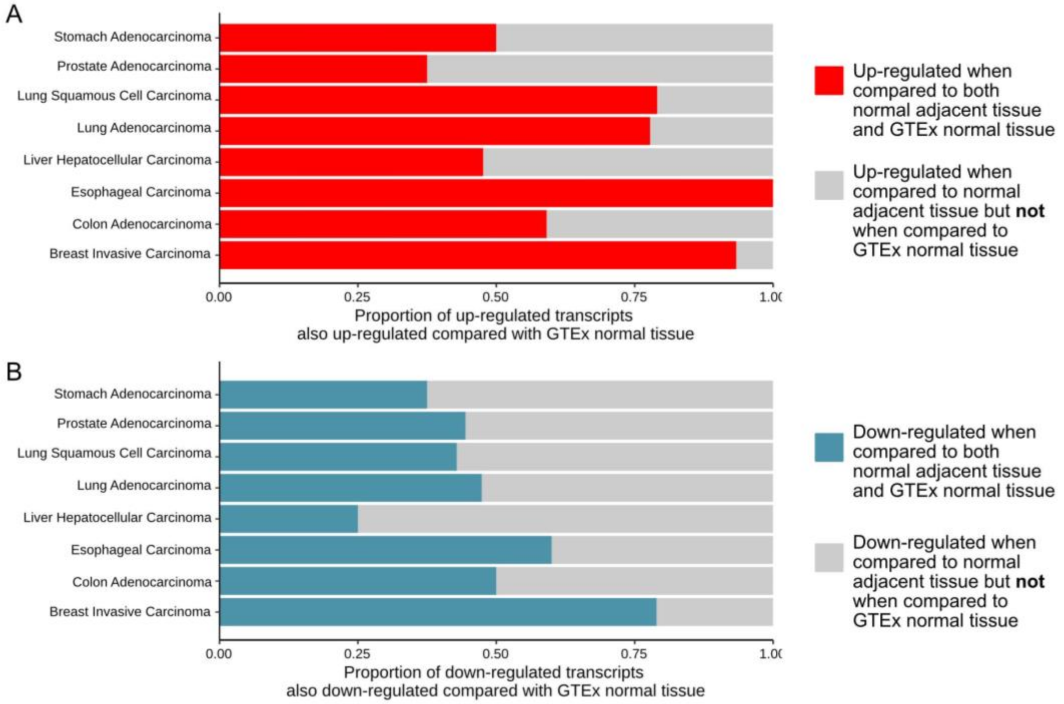

Supplementary Figure 8f

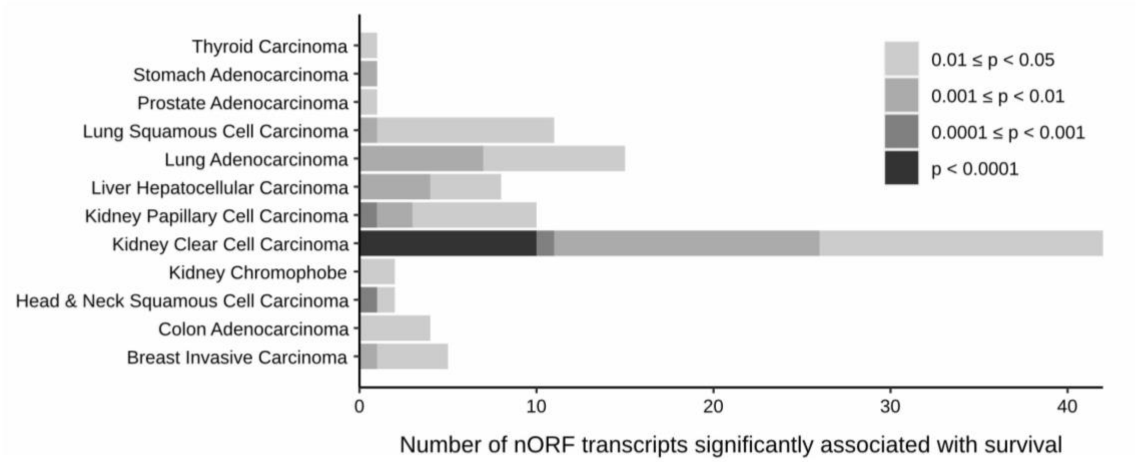

Supplementary Figure 8g

Liver Hepatocellular Carcinoma

	Transcript	nORF ID	Transcript type	Fold change compared with normal adjacent tissue	Survival adjusted p value	Survival hazard ratio
**A**	ENST00000413077.1	hglvH5	processed_transcript	2.75	0.028	0.59
**B**	ENST00000489336.5	io5aH5	processed_transcript	2.25	0.006	0.55

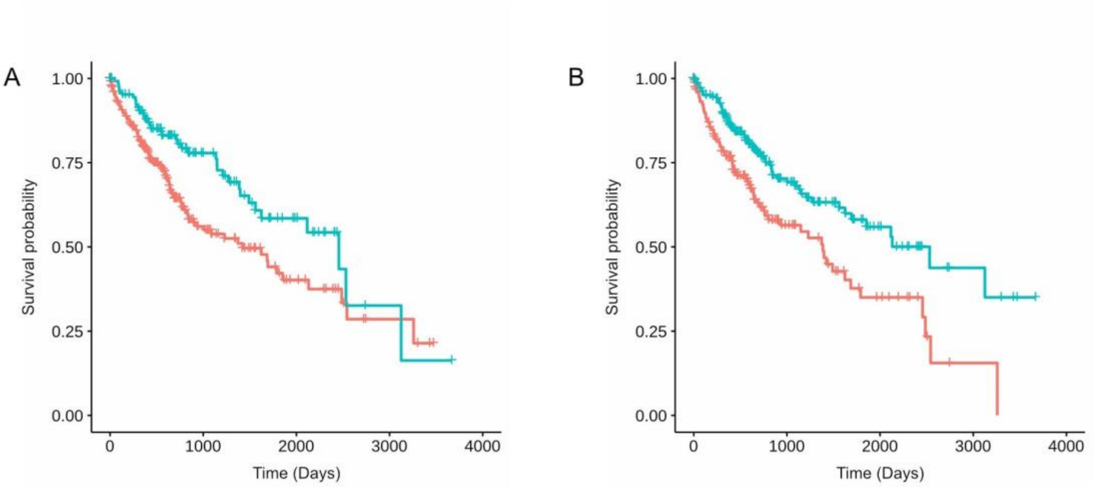

Lung Adenocarcinoma

	Transcript	nORF ID	Transcript type	Fold change compared with normal adjacent tissue	Survival adjusted p value	Survival hazard ratio
**C**	ENST00000536835.2	gbjoH1	antisense	3.07	0.008	0.61
**D**	ENST00000480284.1	tracer_102312	lincRNA	34.30	0.046	0.67

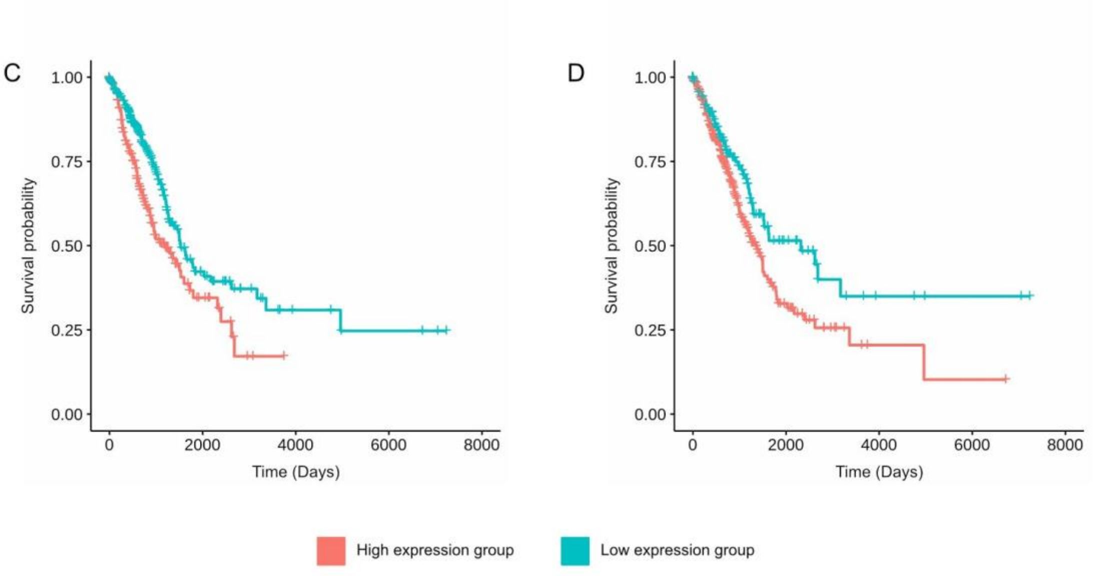

Supplementary Figure 8. Differentially expressed nORF transcripts in cancer, corresponding analysis using a fold change threshold of 1.5, with associated survival analysis. a. total number of differentially expressed nORF transcripts by cancer type compared with NAT **b.** total number of differentially expressed nORF transcripts by cancer type compared with GTEx **c.** nORF transcripts uniquely up- or down-regulated in a single cancer type compared with NAT. **d.** nORF transcripts uniquely up- or down-regulated in a single cancer type compared with GTEx normal tissue. **e.** Reproducibility of differential expression results using normal adjacent tissue and GTEx normal tissue. nORF transcripts identified as differentially expressed when comparing cancer tissue with normal adjacent tissue, showing the proportion of nORF transcripts also differentially expressed when comparing cancer tissue with GTEx tissue (**upper:** up-regulated nORF transcripts, **lower:** down-regulated nORF transcripts) **f.** Association of nORF transcript expression with overall patient survival. Number of differentially expressed nORF transcripts significantly associated with survival at different adjusted p value thresholds, by cancer type. **g.** Kaplan Meier curves showing overall patient survival in high and low expression groups for reproducibly differentially expressed nORF transcripts. Showing Kaplan Meier curves, nORF transcript ID and further transcript details for four nORF transcripts uniquely and reproducibly up-expressed in a single disease, and where high expression is associated with poor prognosis. The cohort was divided into high and low nORF transcript expression groups using the Maximally Selected Rank Statistic, and Kaplan Meier survival curves were generated with a 95% confidence interval. Survival probabilities were compared using the log-rank test and p values adjusted for multiple testing. Overall survival times were fitted to a Cox proportional hazards regression model and hazard ratio calculated from the fitted coefficients.

Supplementary Figure 9a

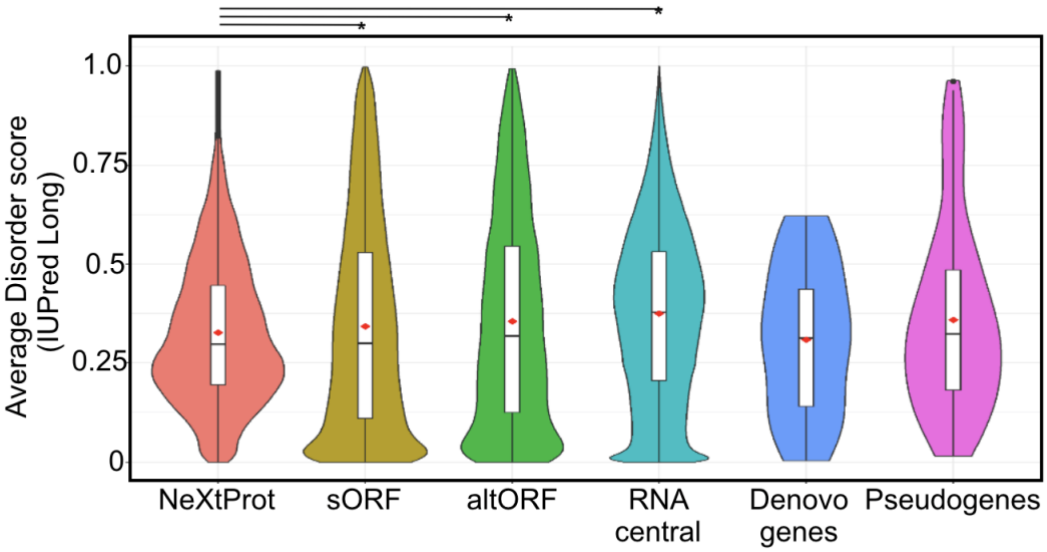

Supplementary Figure 9b

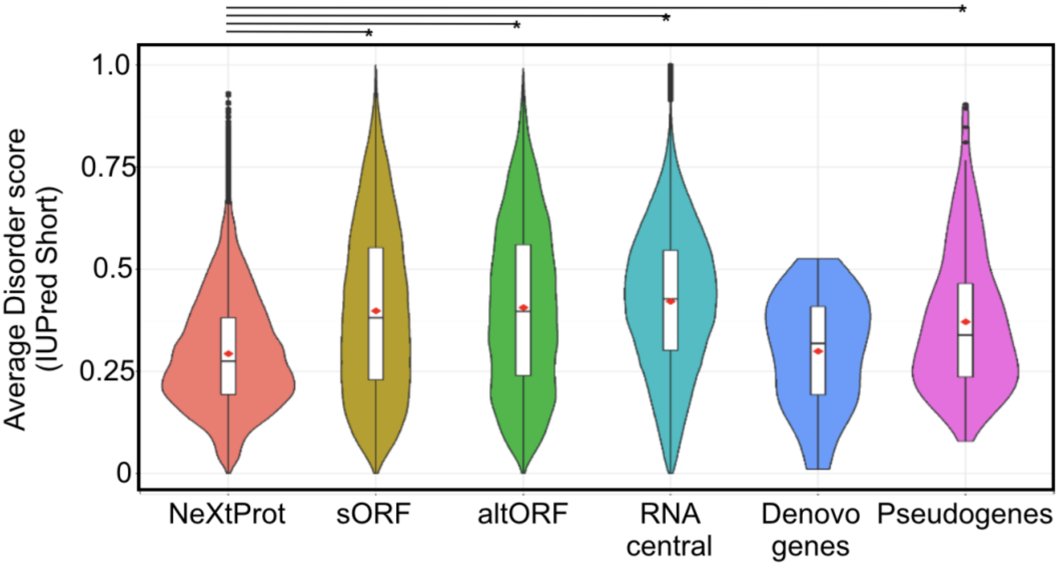

Supplementary Figure 9c

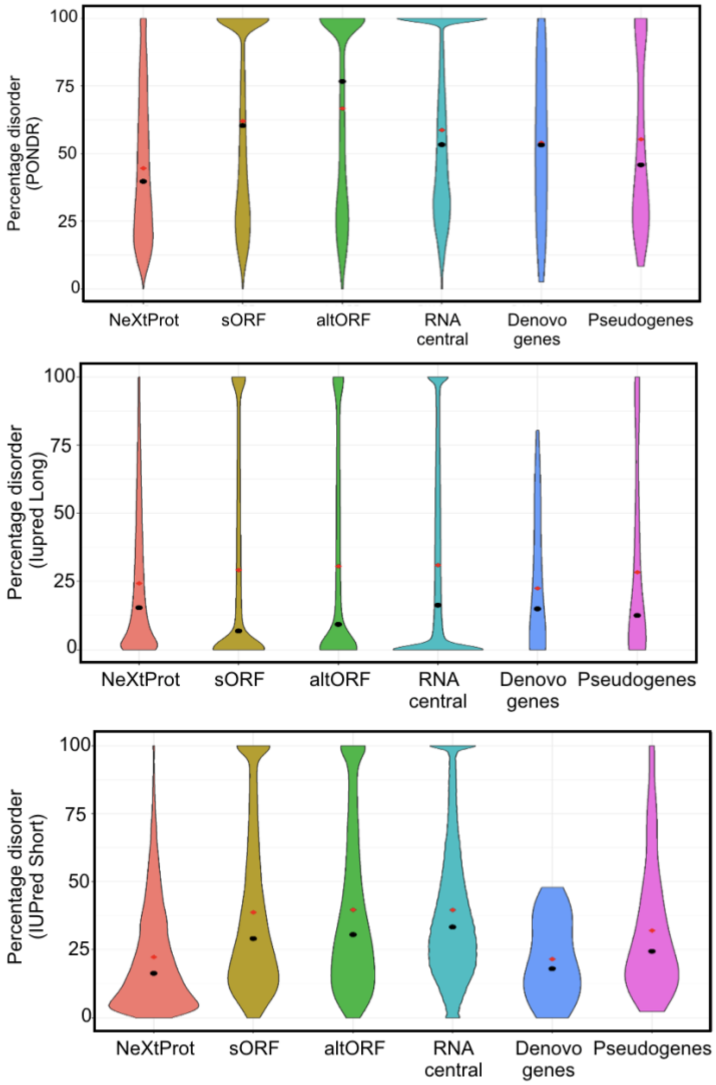

Supplementary Figure 9. Prediction of disorder in proteins encoded by nORFs. Average disorder scores of proteins in NeXtProt compared to average disorder scores of proteins encoded by nORFs, predicted by **a.** IUPred-Long, and **b.** IUPred-Short disorder predictors. **c.** Percentage of protein sequence identified to be disordered (amino-acid disorder score > 0.5) in NeXtProt, and each of the nORF datasets, for three prediction algorithms PONDR (top panel), IUPred-Long (middle panel) and IUPred-Short (bottom panel). Shown in red and black within each distribution are the mean and median respectively.

Supplementary Figure 10a

Dataset	PONDR		IUPred-Long		IUPred-Short	
	Mean	Median	Mean	Median	Mean	Median
NeXtProt	0.486-0.489	0.452-0.457	0.326-0.329	0.295-0.300	0.293-0.296	0.274-0.279
sORF	0.593-0.596	0.554-0.560	0.341-0.344	0.297-0.302	0.397-0.399	0.380-0.384
altORF	0.626-0.632	0.622-0.639	0.352-0.359	0.311-0.326	0.404-0.41	0.392-0.402
RNA Central	0.549-0.550	0.520-0.520	0.375-0.375	0.378-0.378	0.422-0.422	0.428-0.428
Denovo genes	0.457-0.601	0.440-0.638	0.240-0.377	0.175-0.421	0.249-0.352	0.208-0.396
Pseudogenes	0.535-0.601	0.44-0.564	0.325-0.393	0.273-0.361	0.346-0.396	0.310-0.370

Supplementary Figure 10b

Disordered sequences in NeXtProt <	p-value PONDR		p-value IUPred-Long		p-value IUPred-Short	
	Fisher test	Chi-square	Fisher test	Chi-square	Fisher test	Chi-square
sORF	0.00	0.00	0.00	0.00	0.00	0.00
haltORF	0.00	0.00	0.00	0.00	0.00	0.00
RNA Central	0.00	0.00	0.00	0.00	0.00	0.00
Pseudogenes	0.01	0.02	0.06	0.00	0.00	0.00
Denovo genes	0.40	0.66	0.56	0.96	0.90	0.41

Supplementary Figure 10. Statistical significance of predicted disorder scores. a. 95% Bootstrap confidence Interval of the mean and median of disorder scores predicted using PONDR, IUPred-Long and IUPred-Short for each of the nORF datasets. **b.** Statistical significance (uncorrected p-values) for the enrichment of disordered sequences in nORF datasets, in comparison to NeXtProt.

Supplementary Figure 11

Dataset	Overall	Num > 30AA	PONDR	IUPred-Long	IUPred-Short
NeXtProt	42024	41952	17449	7589	3363
sORF	190195	92176	52213	25537	29543
altORF	24676	19281	12037	5725	6523
RNACentral	5185186	5185186	2807585	1534132	1778105
Denovo genes	26	26	12	5	1
Pseudogenes	172	172	87	41	36

Supplementary Figure 11. Number of nORFs investigated. Table showing the number of protein sequences identified in each nORF dataset, number of sequences used for further analysis (Sequence length > 30) and the number of predicted disordered sequences (average disorder score > 0.5) obtained using PONDR, IUPred-Long and IUPred-short algorithms.

Supplementary Figure 12a

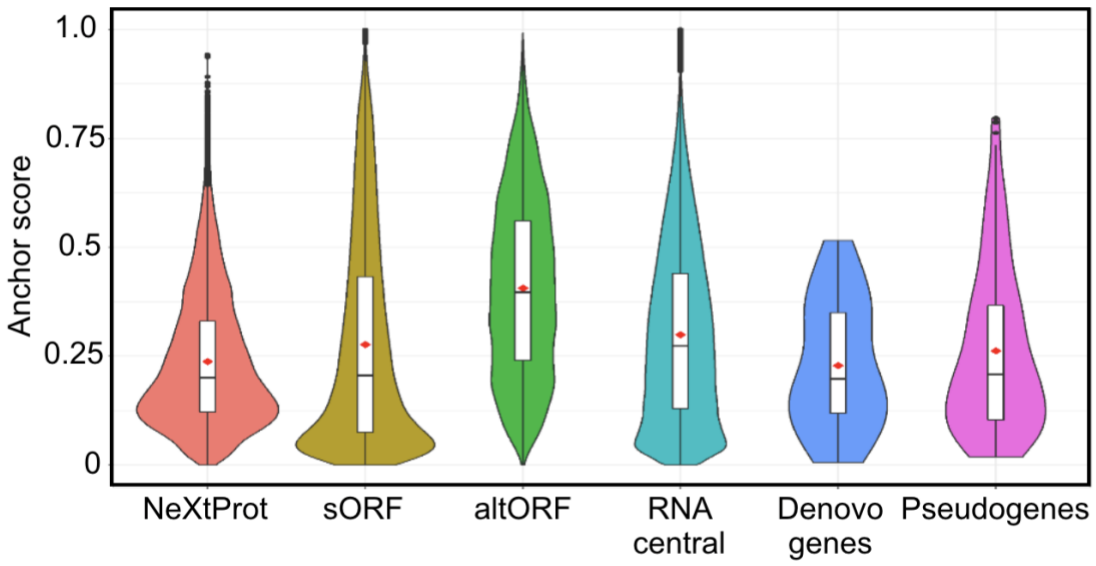

Supplementary Figure 12b

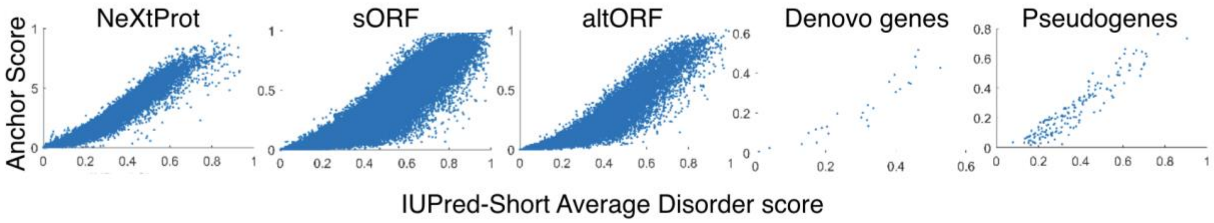

Supplementary Figure 12. Anchor predictions of binding regions in proteins encoded by nORFs. Anchor scores represent the average propensity of an amino acid in a disordered region to be part of a protein-protein binding site. **a.** Shown are the distributions of the predicted Anchor scores for known proteins in NeXtProt, and nORF peptides. As expected, the nORF proteins have higher mean Anchor scores in comparison to the NeXTProt database. **b.** Observed correlation between average Anchor score and IUPred-Short disorder prediction score for individual datasets.

Supplementary Figure 13

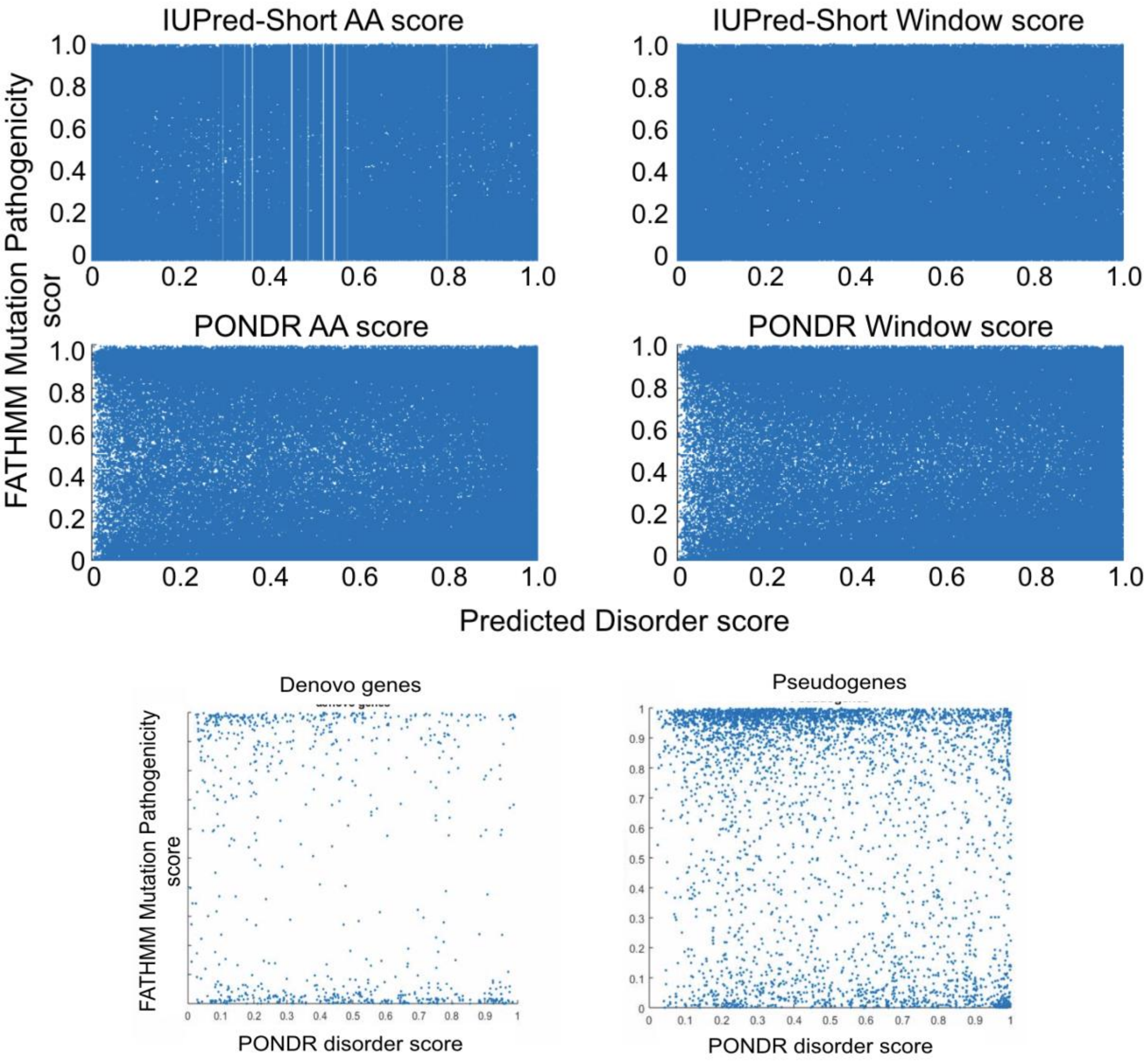

Supplementary Figure 13. FATHMM pathogenicity scores vs predicted disorder scores for proteins encoded by nORFs. We plotted FATHMM mutation pathogenicity scores for the proteins encoded by nORFs, against their corresponding disorder scores predicted using either PONDR and IUPred. Disorder scores were computed at either amino-acid resolution, or for a 7-AA window around the mutated residue. The analysis did not reveal any correlation between FATHMM scores and predicted disorder scores for sORFS, Denovogenes or Pseudogenes.

Supplementary Figure 14

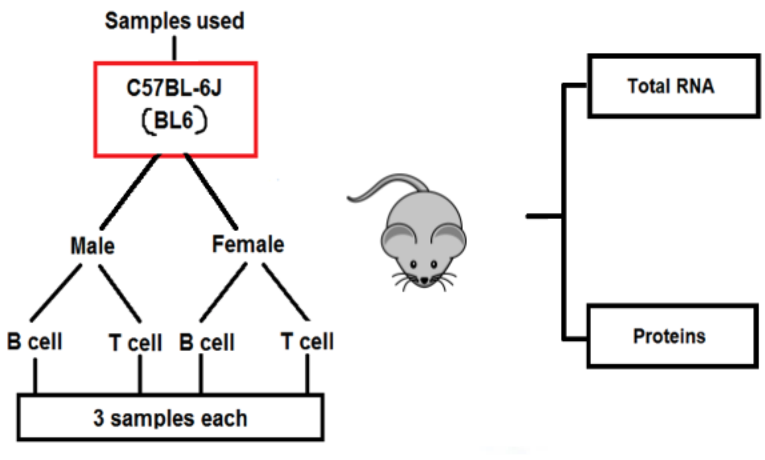

Supplementary Figure 14. Extraction of total RNA and proteins from mouse B and T cells. Naive B and T cells were isolated from the spleen of two sets of six male and six female C57BL/6J mice that were 12 weeks old using FACS. From one set, total RNA was extracted from each of the 12 samples (three B-male, three B-female, three T-male and three T-female) and sequenced. From another set, proteins were extracted and proteins from the same sub-group (B-male, B-female, T-male or T-female) were pooled together for mass spectrometry analysis. Hence, for RNA there are three biological replicates; whereas, for proteins there is only one biological replicate.

Supplementary Figure 15

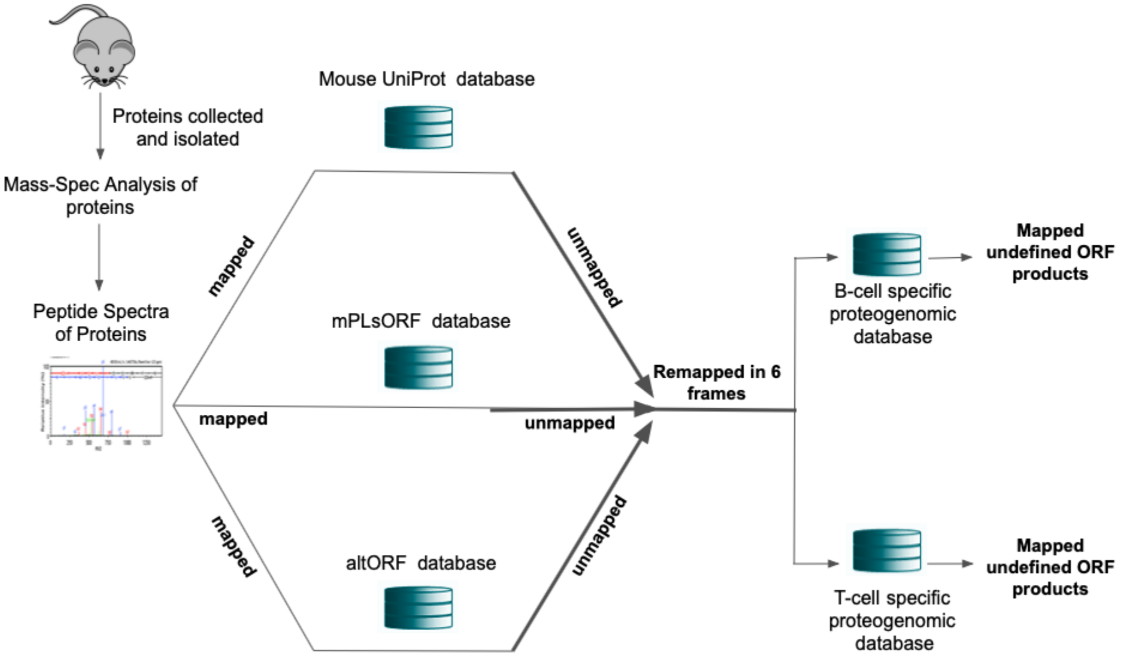

Supplementary Figure 15. Proteogenomic workflow to identify non canonical translated products in mouse B and T cells. Illustrates the schematic workflow of our proteogenomic analysis. Briefly, mass spectra of proteins obtained from mouse B and T cells were independently and sequentially mapped to the following databases in this order (a) mouse UniProt database, (b) an in-house curated sORF database, and (c) the mouse altORF database. Unmapped peptides were remapped to a B and T cell-specific proteogenomic nucleotide database to identify other undefined ORFs.

Supplementary Figure 16

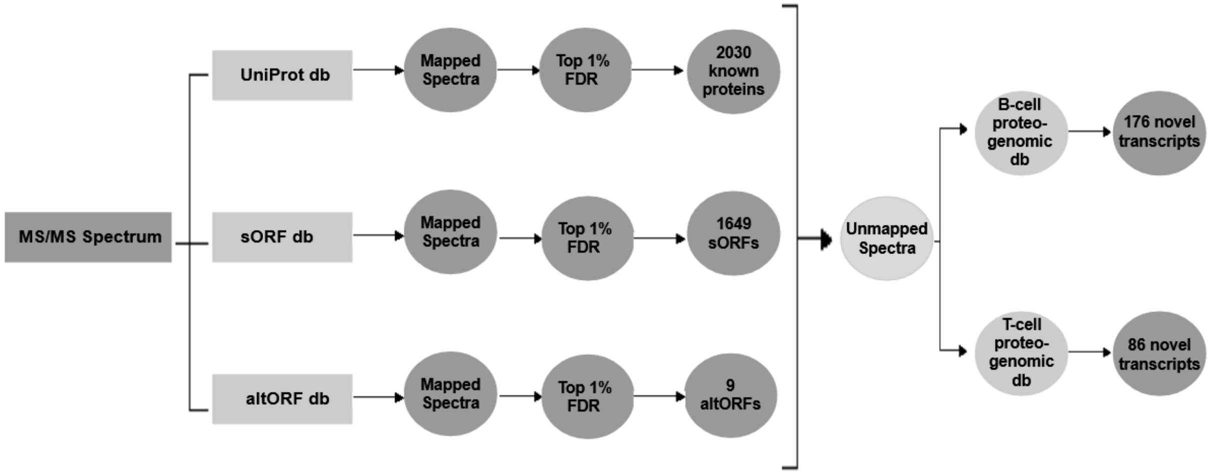

Supplementary Figure 16. Schematic illustration of the proteogenomic workflow used to identify non canonical translated products in mouse B and T cells. 2,030 known proteins, 9 altORFs, 1,649 sORFs, and 259 undefined novel ORF translated products were identified in mouse B and T cells using our proteogenomic workflow.

Supplementary Figure 17

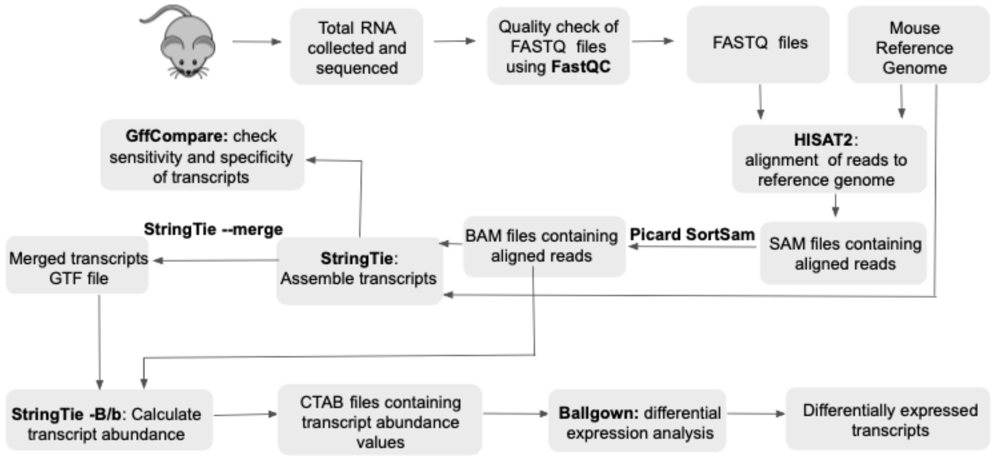

Supplementary Figure 17. Workflow of transcript assembly and differential expression analysis. Total RNA sequenced using Illumina HiSeq 2500 platform were assessed for their quality using FastQC. Read alignment was done using HISAT2, with FASTQ files and reference genome (GENCODE version M12) as inputs. The resulting SAM files containing the aligned reads were converted to BAM using Picard SortSam. Sample BAM files along with the reference genome were used as inputs for transcript assembly using StringTie, for which the assembled transcript quality was assessed using GffCompare. Transcripts assembled across the 12 samples were merged using the StringTie merge function for accurate transcript identification and downstream analysis. StringTie run with the -B/b parameter, using the sample BAM files and the merged transcript list as the reference genome, produced 12 CTAB files for each sample containing details of sample-specific transcript expression levels. These CTAB files were utilised for differential expression analysis using Ballgown. For further details pertaining to each step, refer to the materials and method section.

Supplementary Figure 18

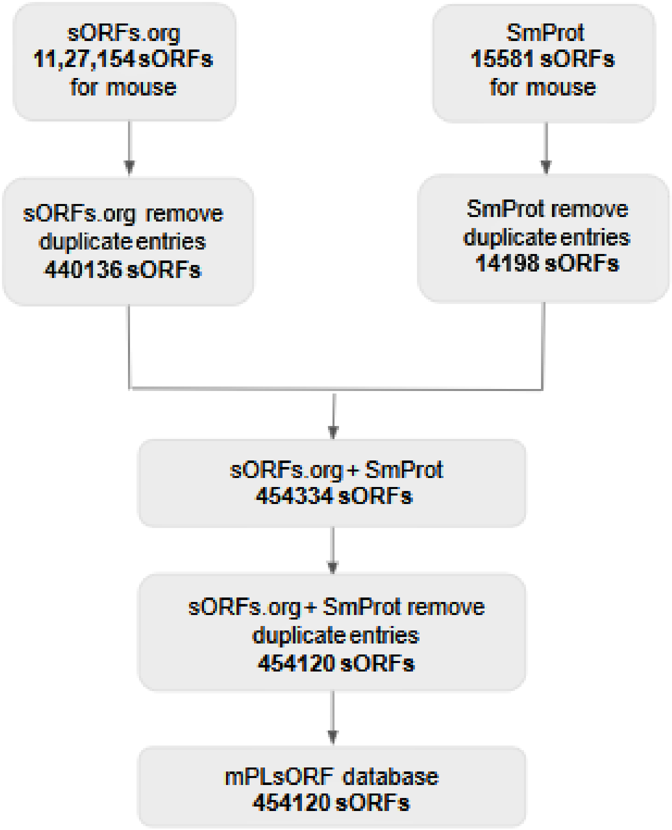

Supplementary Figure 18. Creation of a sORF database. Mouse sORFs for this work were obtained from two sources: sORFs.org containing 1,127,154 sORFs and SmProt containing 15,581 sORFs. Every entry in each of these datasets were individually filtered to remove duplicates resulting in 440,136 sORFs in sORFs.org and 14,198 sORFs in SmProt, finally resulting in 454,120 sORF entries. Each of these sORFs were then assigned a unique identifier and relevant information about the sORFs including their genomic coordinates, strand information, source database, amino acid sequence and genomic annotation were added to create our mPLsORF database.

Supplementary Figure 19

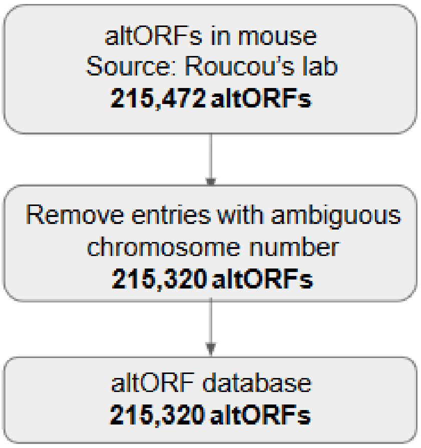

Supplementary Figure 19. Creation of altORF database. Information for the 215,472 mouse altORFs was downloaded from Xavier Roucou's lab, which were processed to remove entries with more than one designated chromosome. Strand information was ascertained separately and added to the database. This analysis resulted in a total of 215,320 altORFs that were used for our analysis.

Supplementary Figure 20

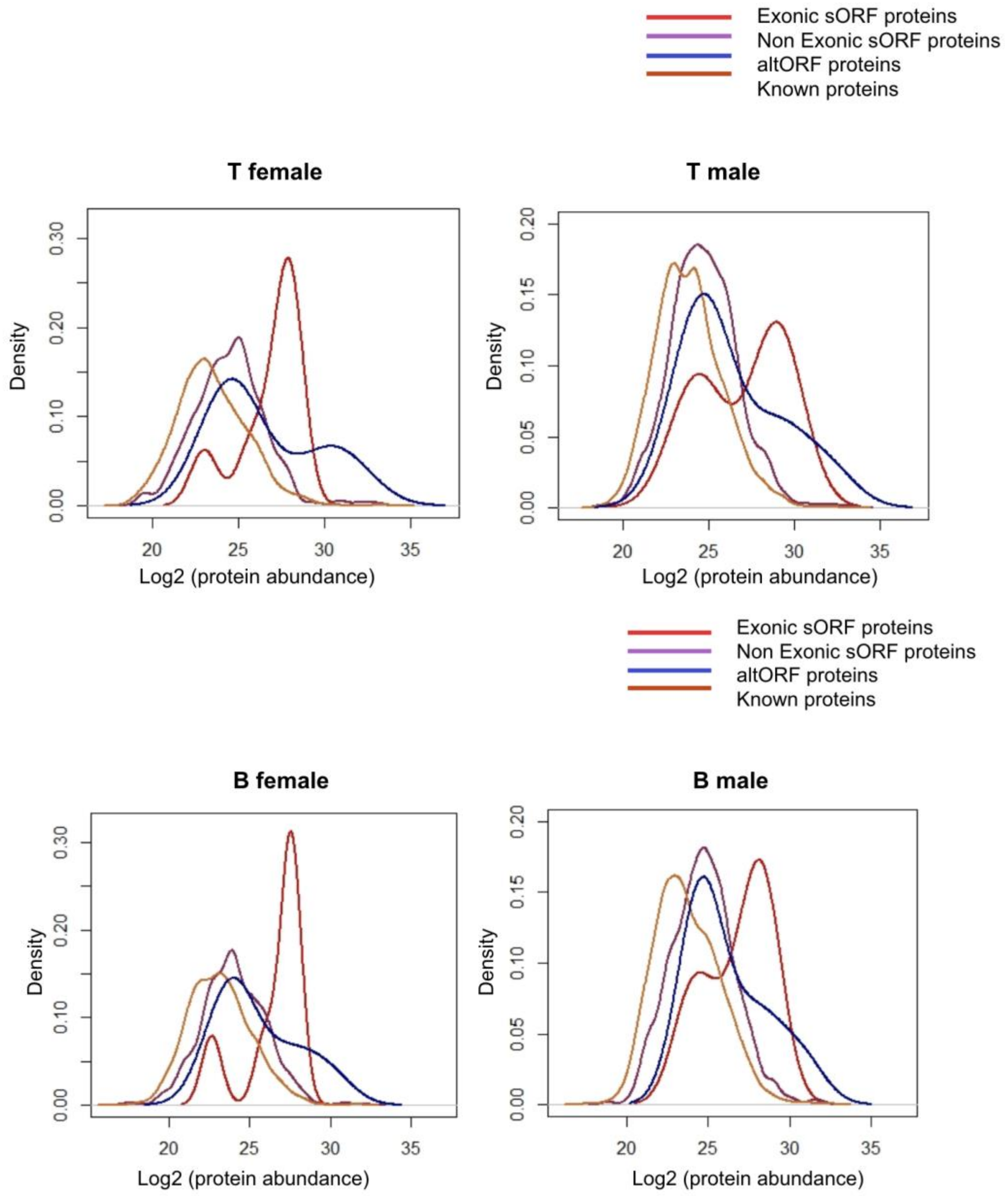

Supplementary Figure 20. Protein abundance distribution plots for different cell groups. Protein abundance plots calculated as $\log_2$ of protein abundance (x-axis) is plotted against density (y-axis) to represent the distribution of protein abundances of exonic sORF (red), non exonic sORF (violet), altORF (blue) and known proteins (dark orange) for T female, T male, B female and B male.

Supplementary Figure 21a

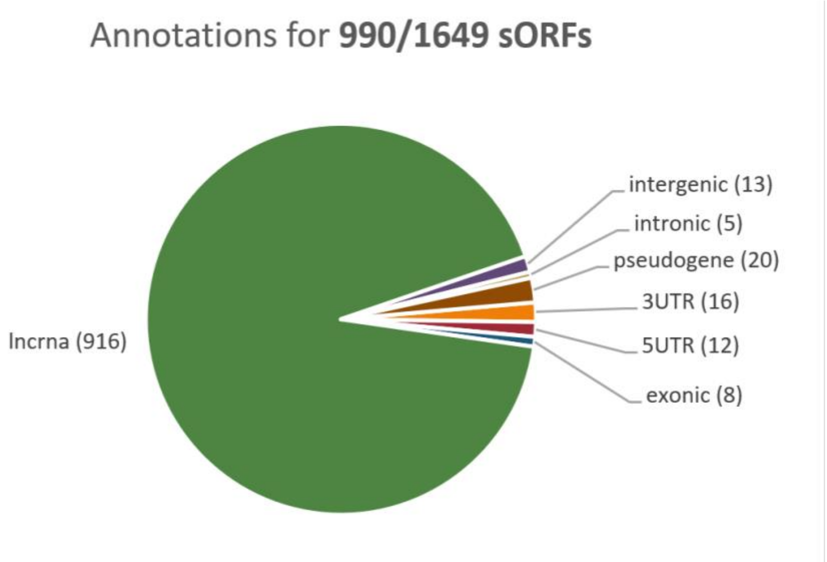

Supplementary Figure 21b

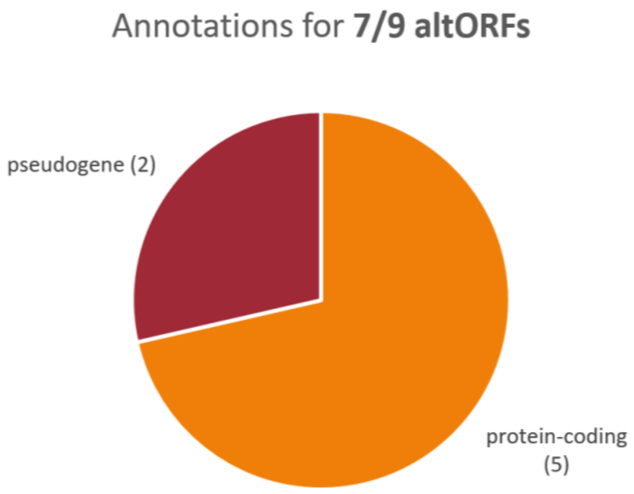

Supplementary Figure 21c

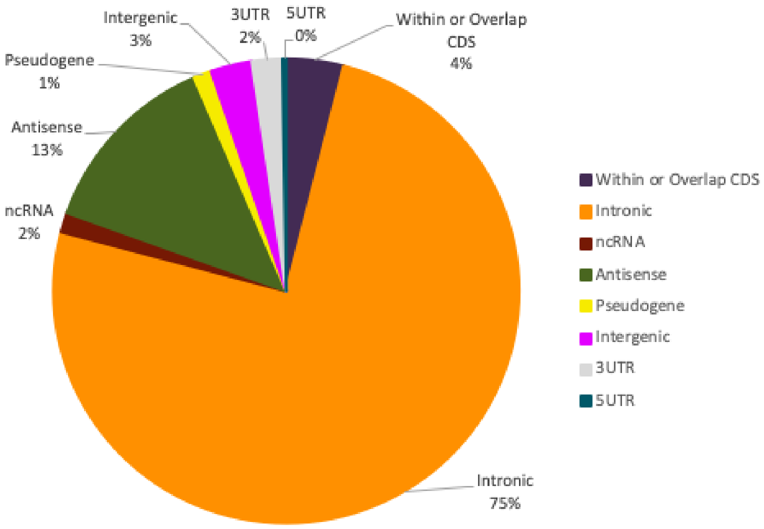

Supplementary Figure 21. Genomic annotations for the different nORF categories found to be translated in B and T cells. a. Pie chart with the different genomic annotations for 990/1649 sORFs. Most of the sORFs are within lncRNAs. **b.** Pie chart with the different genomic annotations for 7/9 altORFs. Most of the altORFs are within protein-coding regions. **c.** Pie chart with the different genomic annotations for 1373/1405 undefined ORFs. Most of the undefined ORFs are within introns.

Supplementary Figure 22

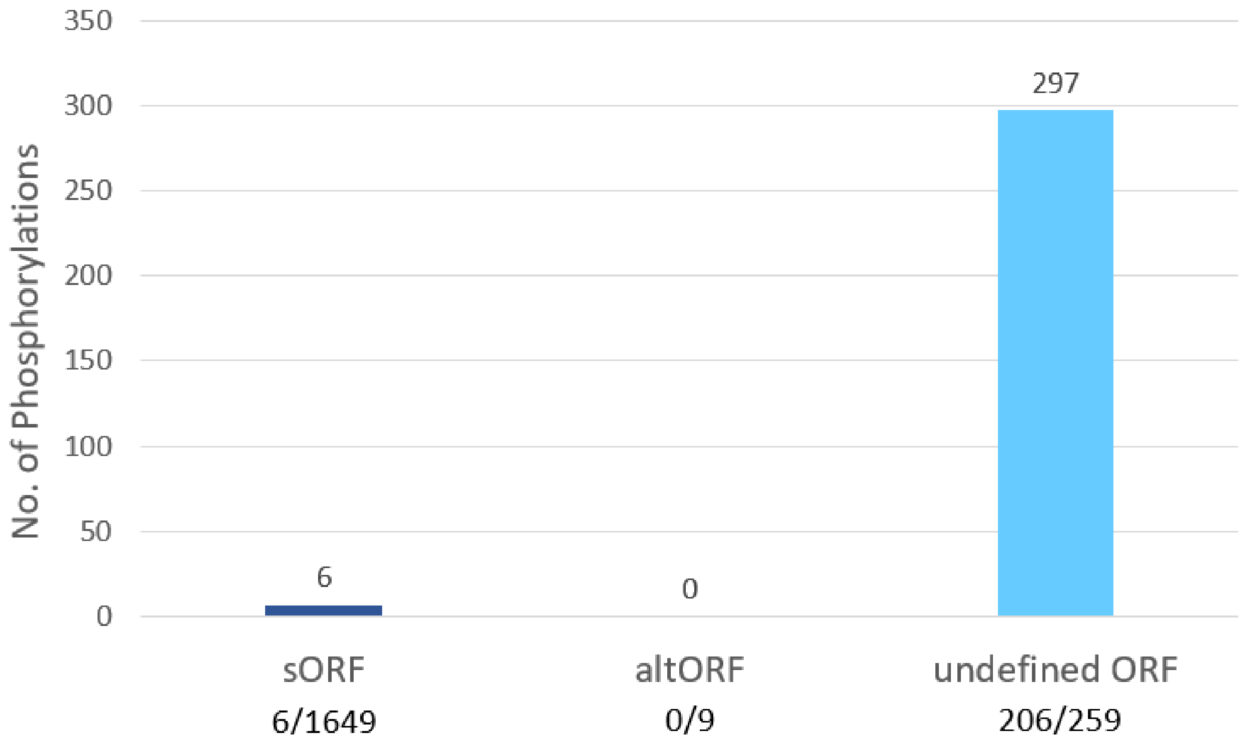

Supplementary Figure 22. Phosphorylation modifications identified within translated nORFs in B and T cells. Translated sORFs, altORFs and undefined ORFs identified in mouse B and T cells were evaluated for the number of phosphorylated sites. 6 sORFs and 206 undefined ORFs were found to contain at least 1 phosphorylated site.

Supplementary Figure 23

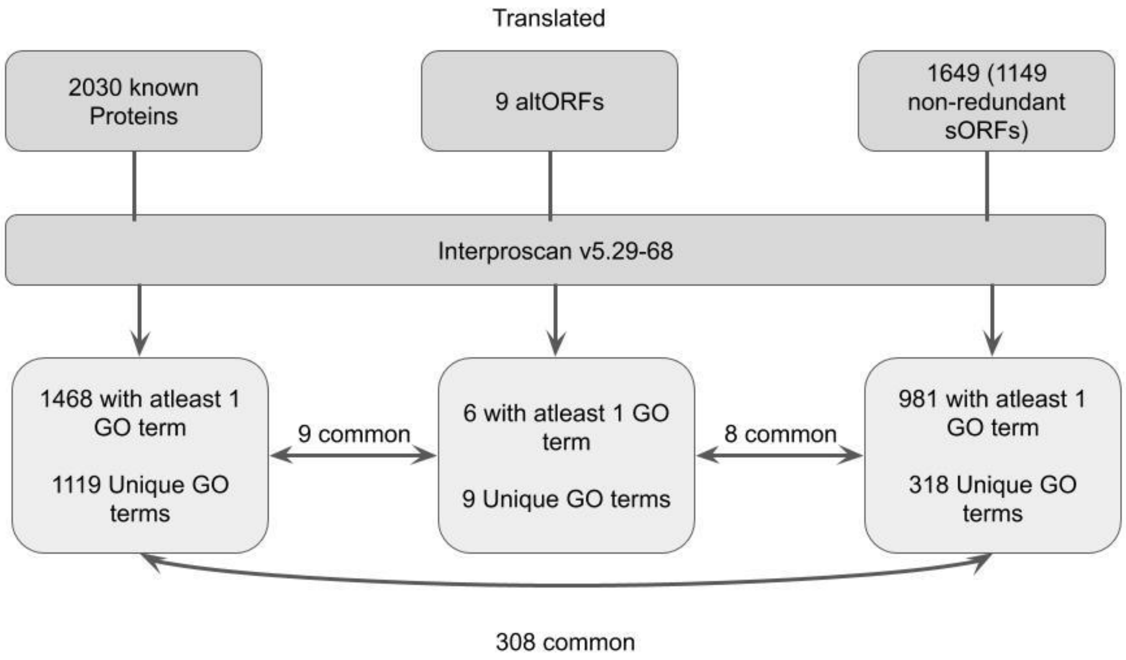

Supplementary Figure 23. Workflow of GO annotation of altORFs and sORFs in preparation for GO analysis. A list of amino acid sequences for all known proteins, altORFs, and sORFs with either transcription or translation evidence in at least 1 of the 12 samples was compiled and analyzed using Interproscan v5.29-68. GO annotations for the known proteins were also generated this way to ensure equal comparison between altORFs and sORFs which are unlikely to have GO annotations from other sources such as experimental methods. The list of 3493 GO terms from these known proteins was then used as the background list for subsequent GO enrichment analysis of sORFs. 490 GO terms present in known proteins were shared with sORFs, and all 73 terms found in altORFs were also present in the known proteins. However, only 46 terms were shared between sORFs and altORFs.

`**Supplementary Figure 24**

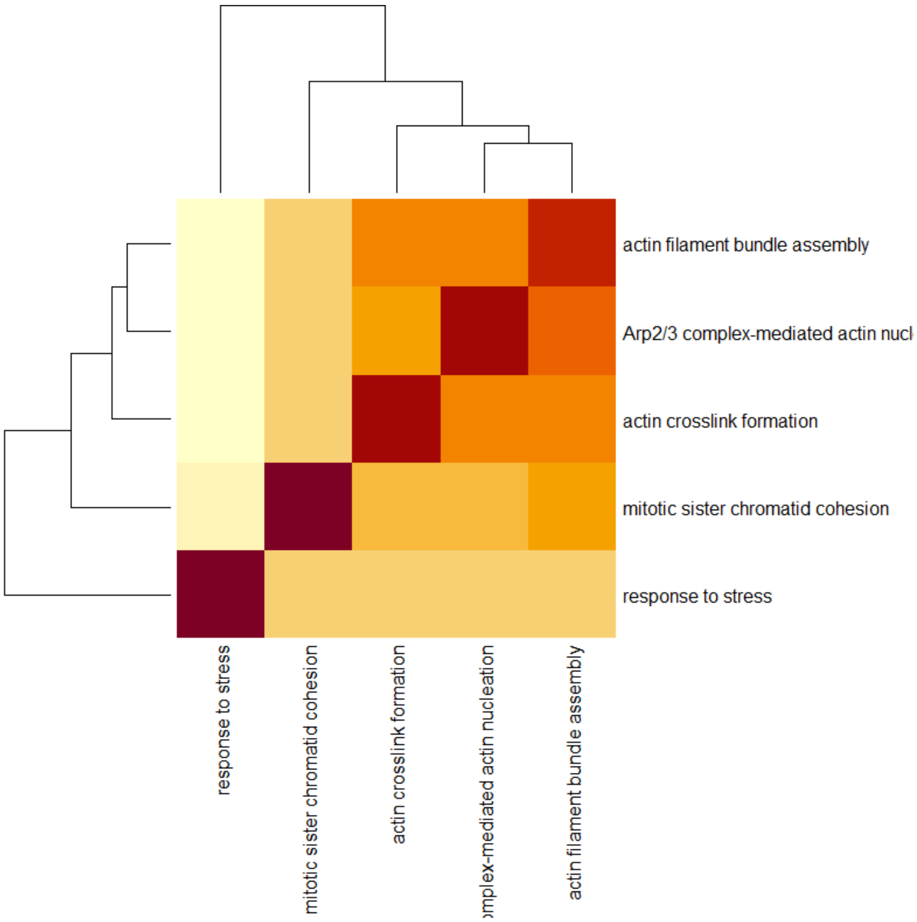

Supplementary Figure 24. Clustering of significantly enriched GO Terms in non-redundant sORFs. Significantly enriched GO terms in non-redundant sORFs were identified using a p-value < 0.01, q-value < 0.01, and proportion more than known protein GO Term proportion. Distances between GO terms were calculated using getTermSim function from bioconductor GOSim package using default settings. The distance metric was then used to cluster the terms to enable easier interpretation by grouping similar GO terms.

Supplementary Figure 25

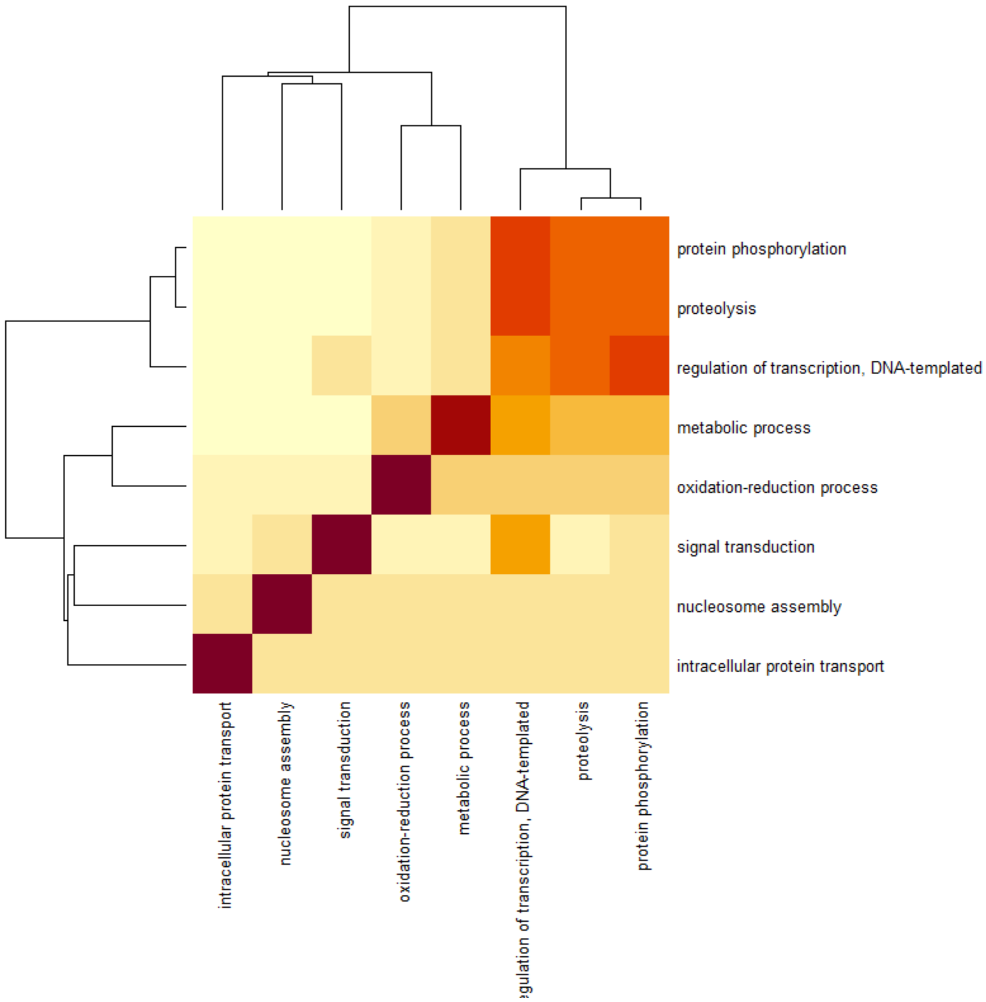

Supplementary Figure 25. Clustering of significantly depleted GO Terms in non-redundant sORFs. Significantly depleted GO terms in non-redundant sORFs were identified using a p-value < 0.01, q-value < 0.01, and proportion less than known protein GO Term proportion. Distances between GO terms were calculated using getTermSim function from bioconductor GOSim package using default settings. The distance metric was then used to cluster the terms to enable easier interpretation by grouping similar GO terms.

Supplementary Figure 26

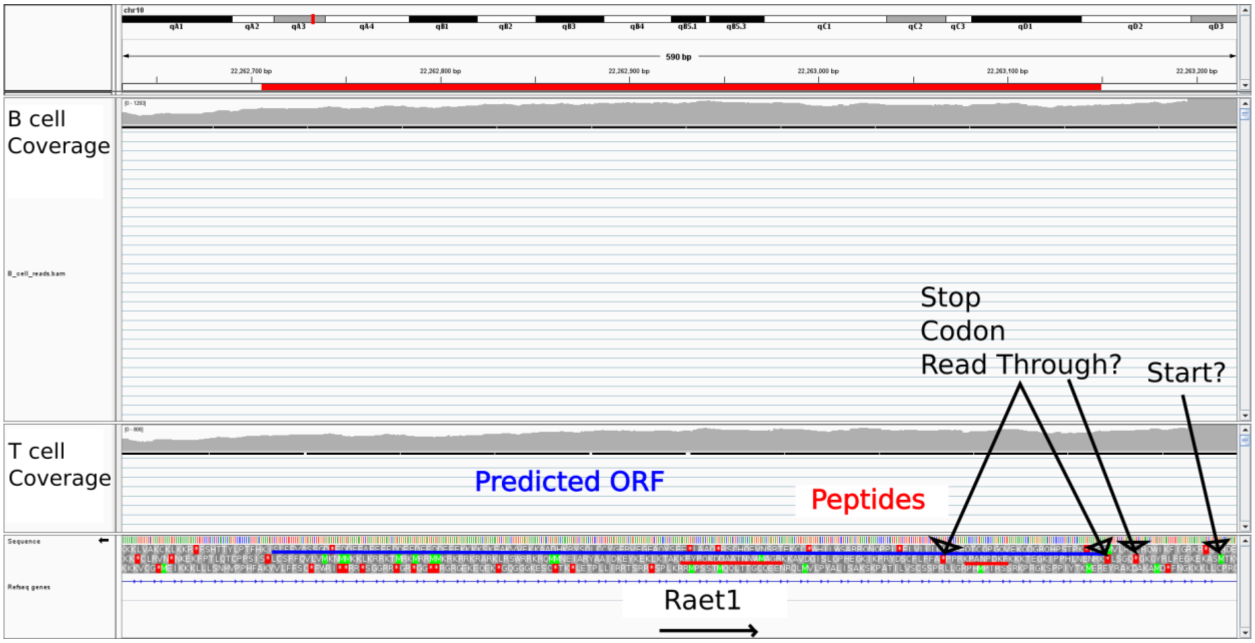

Supplementary Figure 26. Undefined novel ORF antisense to *Raet1* pseudogene. Predicted undefined novel ORF (blue line) was identified by proteogenomics analysis. The novel ORF had two distinct peptides (red lines) mapped to it. The novel transcript has two stop and two start codons.

Supplementary Figure 27

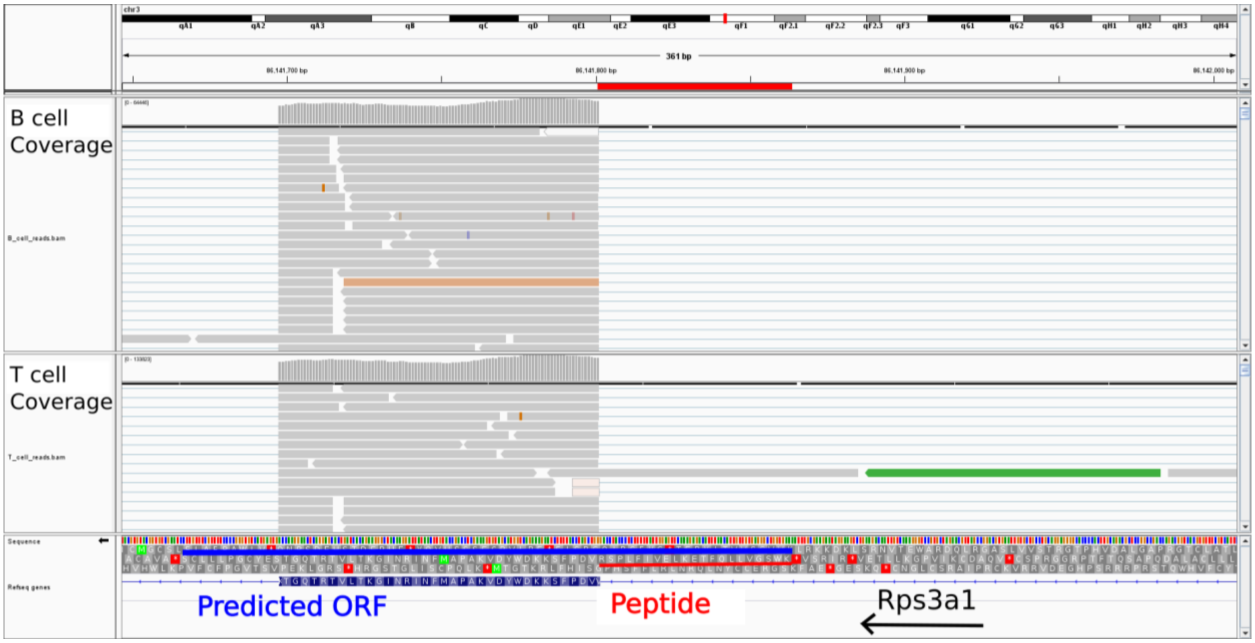

Supplementary Figure 27. Undefined novel ORF as an intron insertion or novel exon of *Rps3a1*. An undefined novel ORF (blue line) spans the exon of *Rps3a1*, a known gene which is a constituent of the 40s component of the ribosome. The identified peptides (red line) map to an unannotated exon and is in the same frame, suggesting it may be incorporated into the exon as an insertion.

Supplementary Figure 28

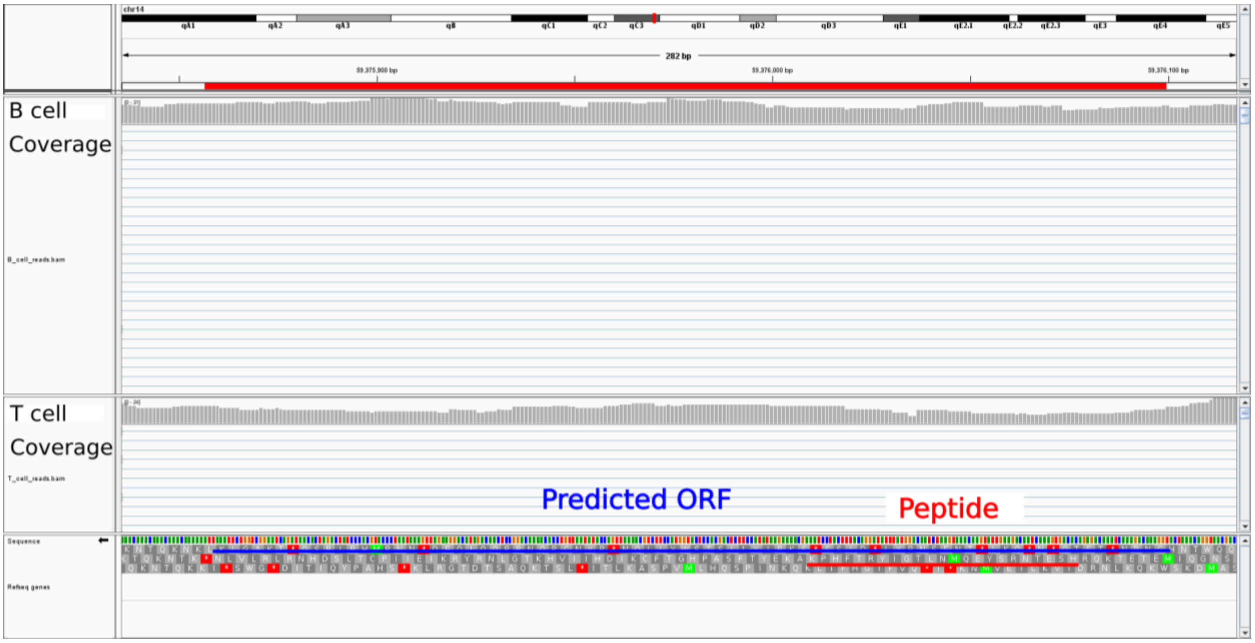

Supplementary Figure 28. Undefined novel ORF in intergenic region on Chr 14. An undefined novel ORF (blue line) in intergenic regions of Chr 14 was identified by proteogenomic analysis and by extension of the aligned peptide fragments (red line) both up and downstream until a stop codon or a start codon was encountered.

Supplementary Figure 29

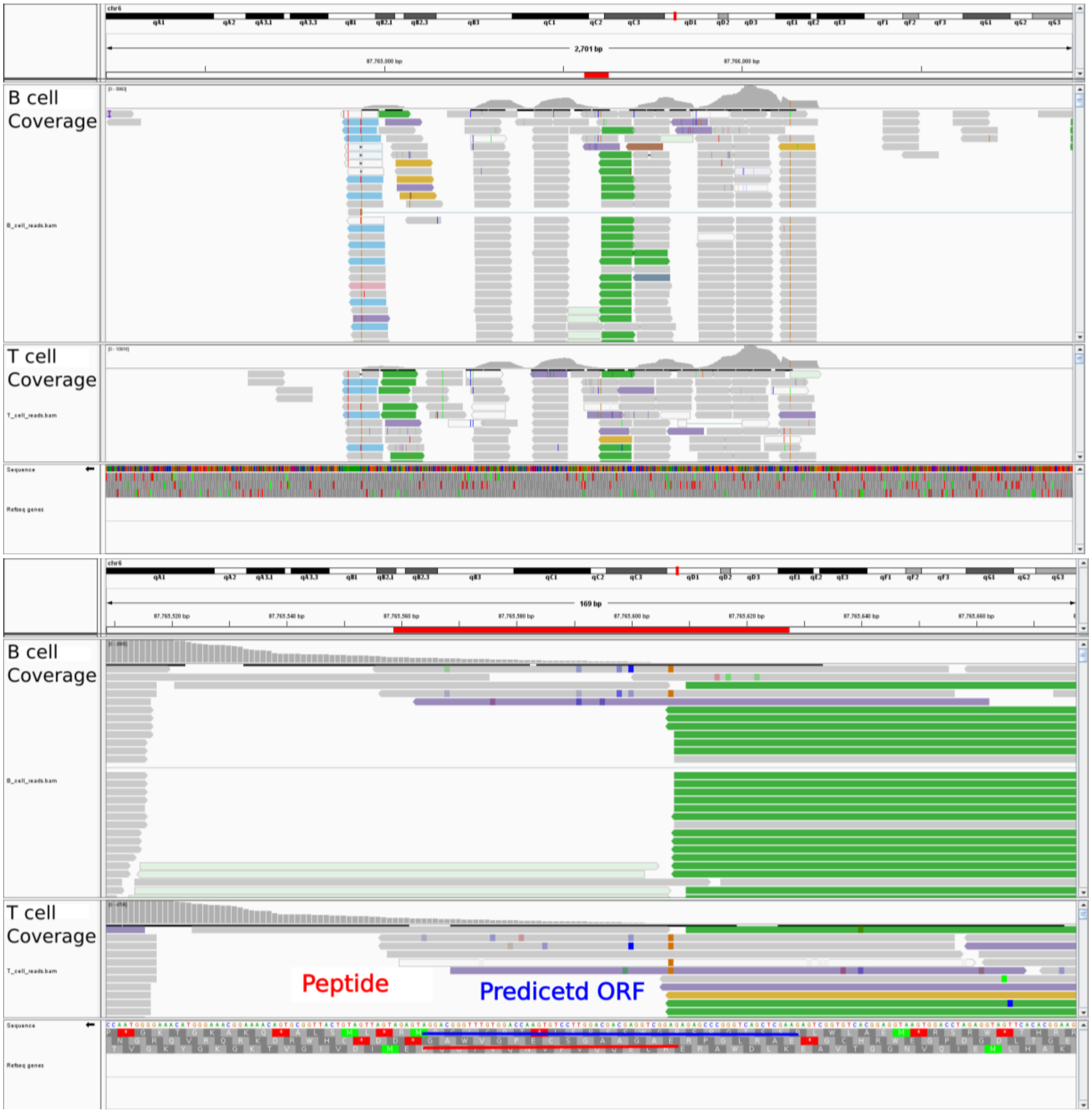

Supplementary Figure 29. An undefined novel ORF is a processed pseudogene ENSMUSG00000068262. The predicted ORF (blue line) was generated by extension of peptide aligned fragments both up and downstream until a stop codon or a start codon was encountered. **Top panel** provides a zoomed-out view of aligned peptides showing where it lies within a relatively low transcribed region of the pseudogene. **Bottom panel** provides a zoomed in view of the pseudogene.

Supplementary Figure 30

GTEx Normal Tissue (n > 50)	TCGA Disease Tissue (n > 50)	TCGA Normal Tissue (n > 10)
	Bladder Urothelial Carcinoma (n=407)	Bladder (n=19)
Brain (n=1148)	Brain Lower Grade Glioma (n=508)	
	Glioblastoma Multiforme (n=152)	
Breast (n=178)	Breast Invasive Carcinoma (n=1091)	Breast (n=113)
Colon (n=307)	Colon Adenocarcinoma (n=285)	Colon (n=41)
Esophagus (n=652)	Esophageal Carcinoma (n=181)	Esophagus (n=13)
	Head & Neck Squamous Cell Carcinoma (n=518)	Head and Neck region (n=44)
	Kidney Chromophobe (n=66)	Kidney (n=129)
	Kidney Clear Cell Carcinoma (n=530)	
	Kidney Papillary Cell Carcinoma (n=288)	
Liver (n=110)	Liver Hepatocellular Carcinoma (n=369)	Liver (n=50)
Lung (n = 288)	Lung Adenocarcinoma (n=513)	Lung (n=109)
	Lung Squamous Cell Carcinoma (n=498)	
Ovary (n=88)	Ovarian Serous Cystadenocarcinoma (n=419)	
Pancreas (n=167)	Pancreatic Adenocarcinoma (n=178)	
Prostate (n=100)	Prostate Adenocarcinoma (n=494)	Prostate (n=51)
Skin (n=555)	Skin Cutaneous Melanoma (n=102)	
Stomach (n=174)	Stomach Adenocarcinoma (n=413)	Stomach (n=36)
Testis (n=165)	Testicular Germ Cell Tumor (n=132)	
	Thyroid Carcinoma (n=504)	Thyroid Gland (n=59)
Uterus (n=78)	Uterine Carcinosarcoma (n=57)	
	Uterine Corpus Endometrioid Carcinoma (n=180)	Endometrium (n=13)

Supplementary Figure 30. Samples and tissues included in this study. Tissues were included in this study where they had n > 50 samples in the case of TCGA cancer tissues and GTEx normal tissues, and n > 10 samples in TCGA normal adjacent tissue (NAT). Bars indicate matching cancer cohorts and reference tissues. Identification of frequently expressed transcripts and differential expression analysis were performed separately for cancer tissues with NAT or GTEx normal tissue.

Supplementary Figure 31

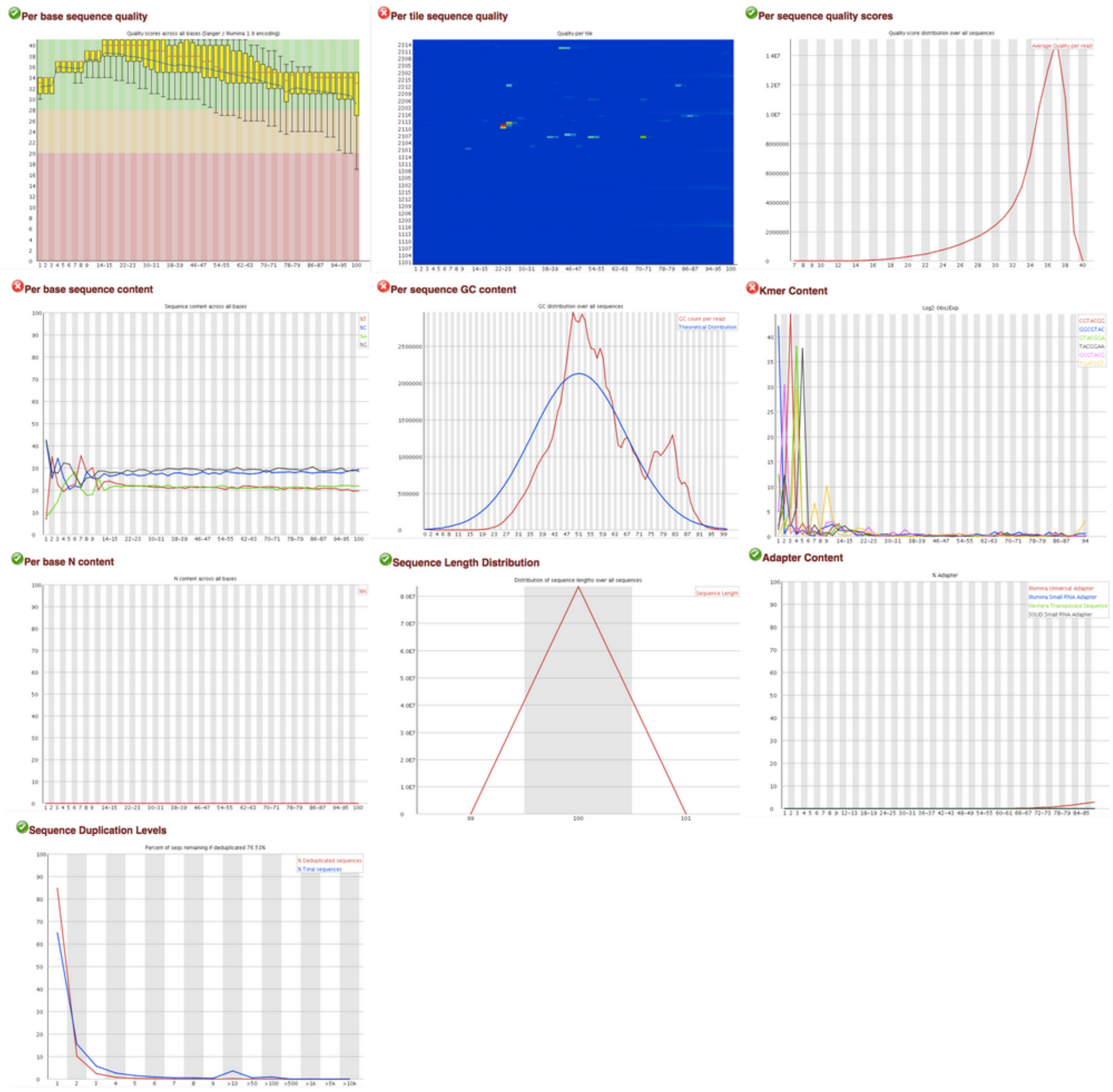

Supplementary Figure 31. Representative raw read quality metrics generated by FastQC. The 10 panels shown are a representative FastQC report for the first set of reads for one of the female T cell samples. The ticks or crosses present in each frame represent how the data compare to the quality thresholds defined by FastQC (www.bioinformatics.babraham.ac.uk/projects/fastqc/Help) and do not necessarily imply that the data are inappropriate for analysis for the purpose of this project. In particular, the expected distribution of the per sequence GC content (blue line) does not refer specifically to the mouse transcriptome.

Supplementary Figure 32

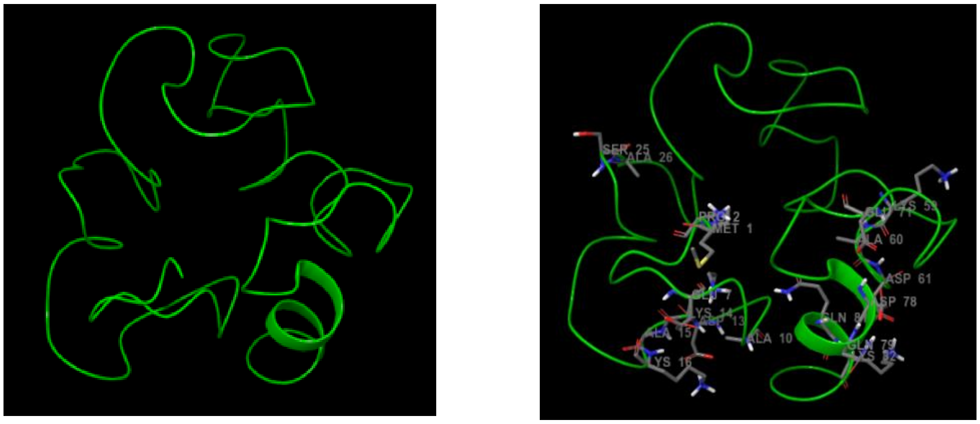

Supplementary Figure 32: Evfold predicted structure of the translated product of ENST00000427352.1 (left) with the marked active site residues(right)

Supplementary Figure 33

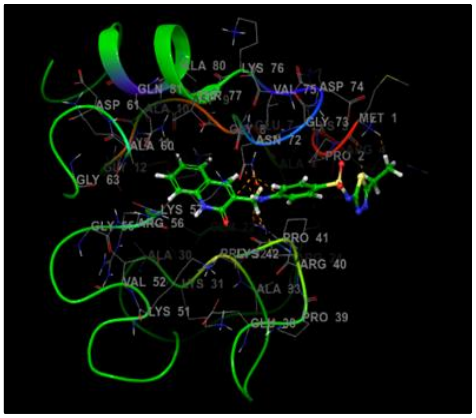

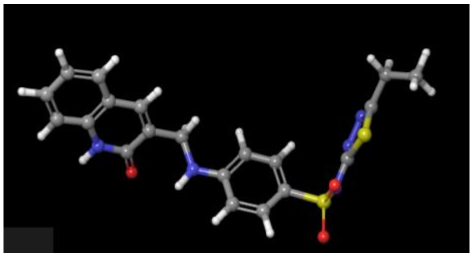

Supplementary Figure 33: Structure of the predicted best hit molecule from the immunooncolgy molecule (compound 8462) (bottom) and its complex with the target protein (top). Interacting residues are: Asn 72, Arg 40 and Met 1

Supplementary Figure 34

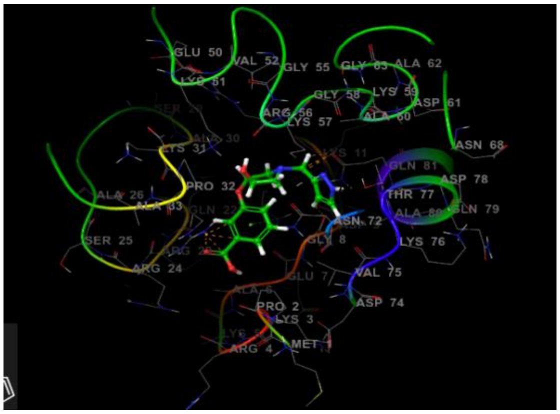

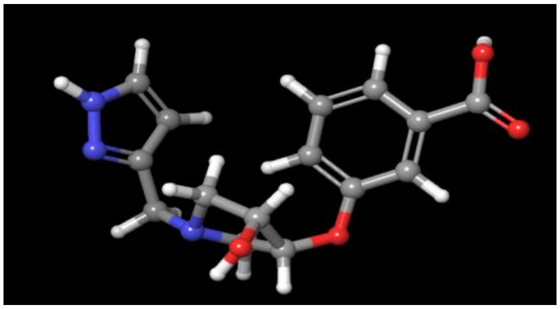

Supplementary Figure 34: Structure of the predicted best hit molecule (compound 1491) (bottom) and its complex with the target protein (top). The interacting residues are Gly8, Arg40, Lys51, Lys57 and Gln81.

Supplementary Figure 35

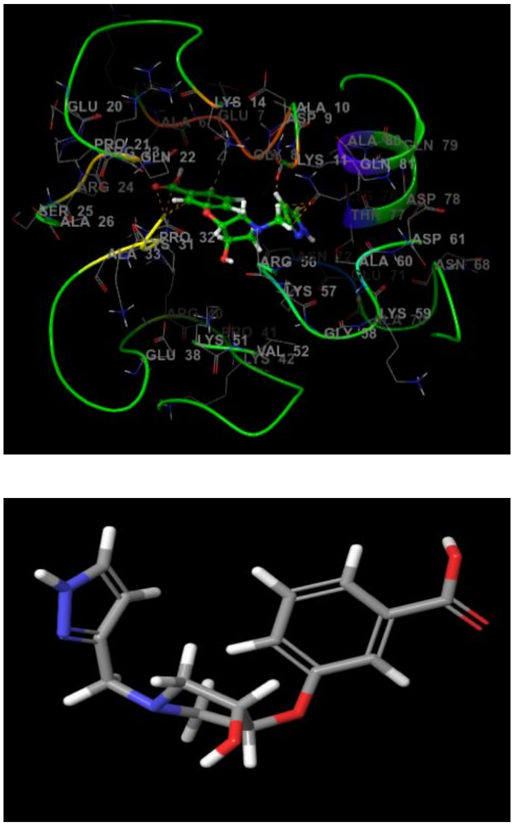

Supplementary Figure 35: Structure of the predicted best hit molecule (compound 1355) (bottom) and its complex with the target protein (top). The interacting residues are Gly8, ARG 40 and Gln81

Supplementary Table 1

Sample ID	GEO Sample ID	Sample identify
10966_8_1	GSM2480724	B cell female
10966_8_2	GSM2480725	B cell female
10966_8_3	GSM2480726	B cell female
11048_5_13	GSM2480727	B cell male
11048_5_14	GSM2480728	B cell male
11048_5_15	GSM2480729	B cell male
11049_4_4	GSM2480730	T cell female
11049_4_5	GSM2480731	T cell female
11049_4_6	GSM2480732	T cell female
11049_5_16	GSM2480733	T cell male
11049_5_17	GSM2480734	T cell male
11049_5_18	GSM2480735	T cell male

Supplementary Table 1. Information and Sample ID for mice used in transcriptomic analysis. Sample IDs used in the transcriptomic analysis, their corresponding GEO sample ID and sample information is provided in the table. GEO sample id can be accessed using GEO accession: GSM2480756.

Supplementary Table 2

Gene	Disease Phenotype	Mutation counts
ACTG1	Baraitser-Winter_syndrome	1
ACTN4	Glomerulosclerosis_focal_and_segmental	1
AK2	Reticular_dysgenesia	2
ARCN1	Craniofacial_syndrome	2
ATP2A2	Schizophrenia	1
ATP2A2	Darier_disease	17
ATP2A2	Acrokeratosis_verruciformis	1
BCLAF1	Colorectal_cancer	1
BUB3	Variegated_aneuploidy	1
CALM1	Catecholaminergic_polymorphic_ventricular_tachycardia	2
CALR	Schizoaffective_disorder	1
DDX3X	Intellectual_disability	5
DDX5	Fibrosis_risk_association_with	1
DYNC1H1	Malformations_of_cortical_development	1
FLNA	Thoracic_aortic_aneurysms_and_dissections	1
FLNA	Thoracic_aortic_aneurysms	1
FLNA	Otopalatodigital_syndrome_2	6
FLNA	Otopalatodigital_syndrome_1	4
FLNA	Mental_retardation_X-linked	1
FLNA	Melnick-Needles_syndrome_epilepsy_&_heterotopia_periventricular_nodular	1
FLNA	Lower_resp._tract_infection_bilateral_lung_emphysema_with_basal_atelectasis_bronchospasm_and_pulmonary_artery_hypertension	1
FLNA	Heterotopia_periventricular_with_skeletal_dysplasia	1
FLNA	Heterotopia_periventricular_nodular	7
FLNA	Heterotopia_periventricular	12
FLNA	Heterotopia_nodular	1
FLNA	Frontometaphyseal_dysplasia	1
FLNA	FG_syndrome	1
FLNC	Frontotemporal_dementia_behavioural_variant	1
FLNC	Cardiomyopathy_hypertrophic	1
GOT1	Aspartate_aminotransferase_deficiency	1
GPI	Glucosephosphate_isomerase_deficiency	2
HIST3H3	Intellectual_disability	1
HNRNPU	Lennox-Gastaut_syndrome	1
HNRNPU	Epileptic_encephalopathy	1
HSPA9	Parkinson_disease	1
HSPA9	EVEN-PLUS_syndrome	1
LDHA	Lactate_dehydrogenase_deficiency	1
LDHB	Lactate_dehydrogenase_deficiency	2
LMNA	Ventricular_arrhythmia	1
LMNA	Spinal_muscular_atrophy_with_cardiac_involvement	1

LMNA	Peripheral_neuropathy	1
LMNA	Partial_lipodystrophy_atypical	1
LMNA	Muscular_dystrophy_limb_girdle_with_severe_heart_failure_and_lipodystrophy	1
LMNA	Muscular_dystrophy_limb_girdle	9
LMNA	Muscular_dystrophy_Emery-Dreifuss	10
LMNA	Muscular_dystrophy	5
LMNA	Cardiomyopathy_right_ventricular_&_Charcot-Marie-Tooth_disease_2B1	1
LMNA	Cardiomyopathy_dilated_with_conduction_defect_type_1A	1
LMNA	Cardiomyopathy_dilated	23
LMNA	Cardiac_disease	1
LMNA	Cardiac_conduction_system_disease	1
LMNA	Cardiac_conduction_defects	1
LMNA	Arrhythmogenic_right_ventricular_cardiomyopathy	1
MBNL1	Myotonic_dystrophy	1
MECP2	Rett_syndrome_preserved_speech_variant	1
MECP2	Rett_syndrome_atypical	1
MECP2	Rett_syndrome	12
MECP2	Non-fatal_non-progressive_encephalopathy	1
MECP2	Neonatal_encephalopathy_severe	1
MECP2	Mental_retardation_X-linked	3
MECP2	Mental_retardation	1
MECP2	Autism_spectrum_disorder	1
MECP2	Autism	1
PAFAH1B1	Subcortical_band_heterotopia	1
PAFAH1B1	Miller-Dieker_lissencephaly_syndrome	1
PAFAH1B1	Lissencephaly_isolated	10
PFN1	Amyotrophic_lateral_sclerosis_association_with	1
PFN1	Amyotrophic_lateral_sclerosis	6
PPIB	Osteogenesis_imperfecta_recessive	1
PPIB	Osteogenesis_imperfecta_II	1
PPIB	Osteogenesis_imperfecta	2
PPP2R1B	Breast_cancer	1
RPS19	Diamond-Blackfan_anaemia	31
SIN3A	Intellectual_disability_mild	1
SLC25A12	AGC1_deficiency	1
SMC1A	Developmental_delay_epilepsy_delayed_speech_&_encephalopathy	1
SMC1A	Cornelia_de_Lange_syndrome	7
SPTAN1	Intellectual_disability	1
STAT1	Mycobacterial_infection	1
STAT1	Impaired_mycobacterial_immunity	1
TALDO1	Transaldolase_deficiency	2
TUBA4A	Amyotrophic_lateral_sclerosis	3
VIM	Congenital_cataract	1

Supplementary Figure 2. List of genes, associated disease phenotype and number of HGMD mutations corresponding to the phenotype. Table to accompany **Figure 5c**. The list of gene names mentioned in the legend of Figure 5C is listed in column 1 and the disease phenotype associated with it in column 2. Column 3 highlights the number of HGMD mutations associated with the particular gene and disease phenotype.

Supplementary Table 3

**mPLsORF0000299804_significant_ECs_0** 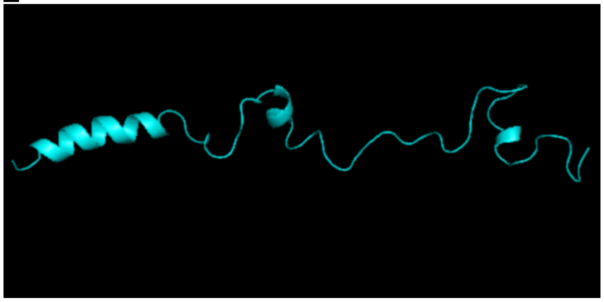	**mPLsORF0000442197_significant_ECs_0** 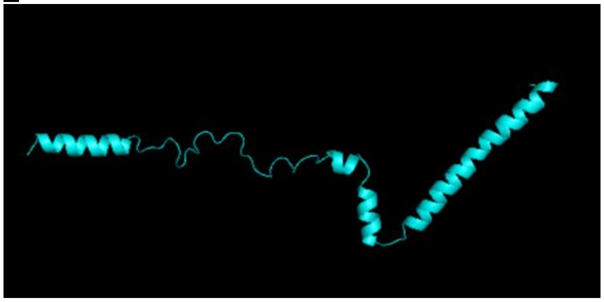
**mPLsORF0000453204_68_8_hMIN** 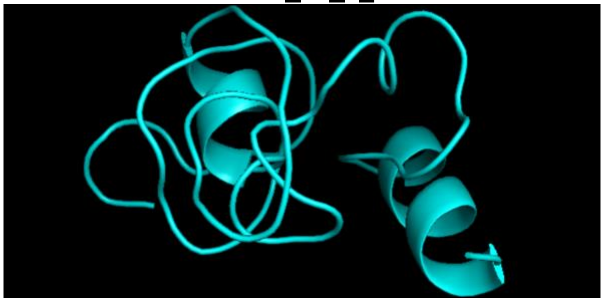	**mPLsORF0000445046_35_7_hMIN** 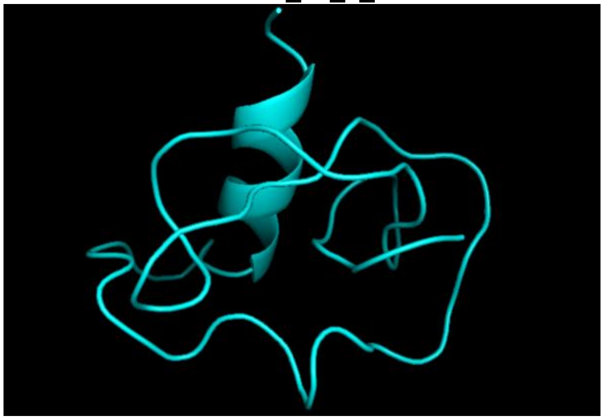
**mPLsORF0000452527_significant_ECs_0** 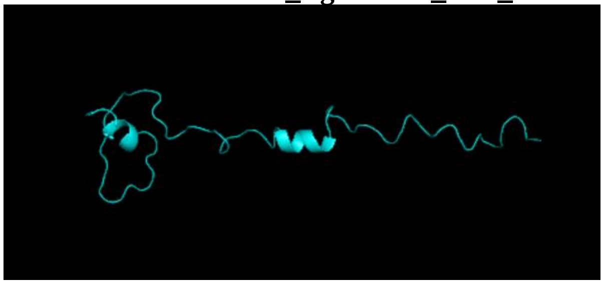	**mPLsORF0000137329_55_2_hMIN** 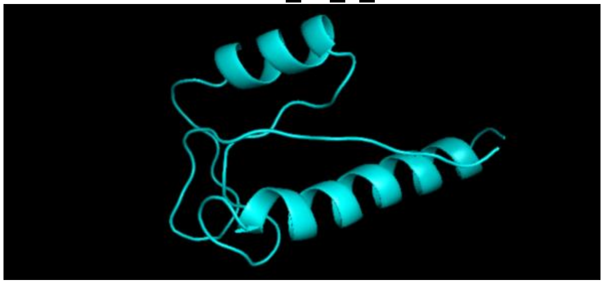

mPLsORF0000453225_56_10_hMIN

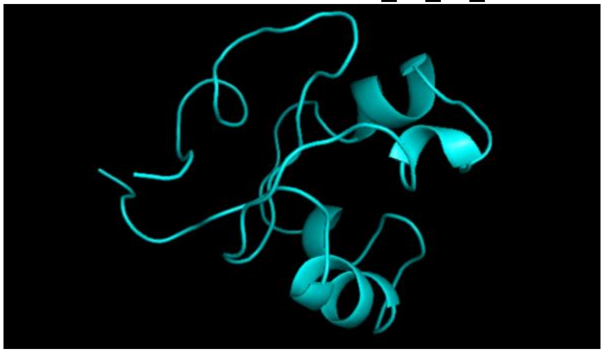

mPLsORF0000449632_76_3_hMIN

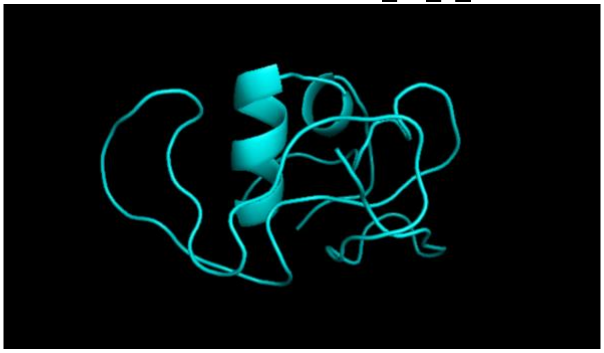

mPLsORF0000443365_43_8_hMIN

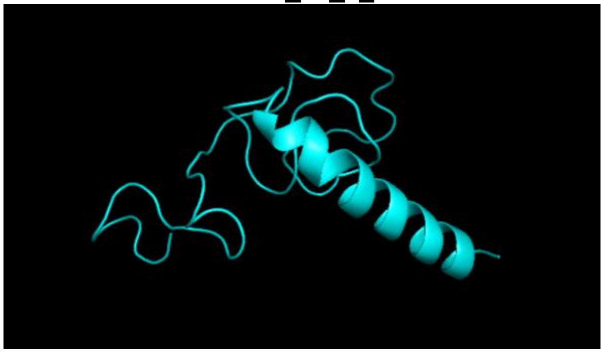

mPLsORF0000450681_significant_ECs_0

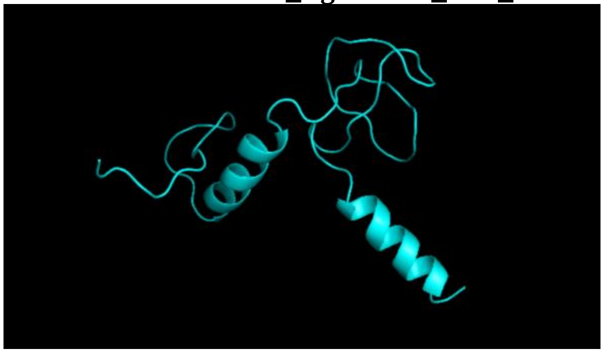

mPLsORF0000451320_43_8_hMIN

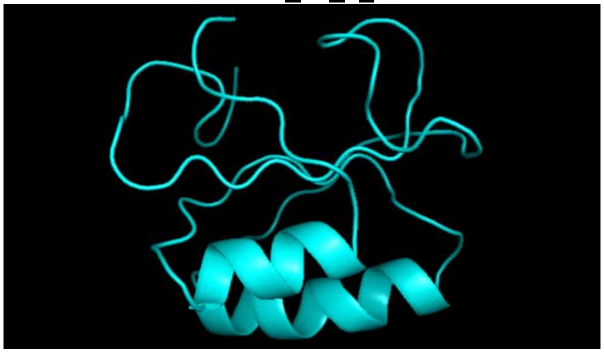

mPLsORF0000114575_63_6_hMIN

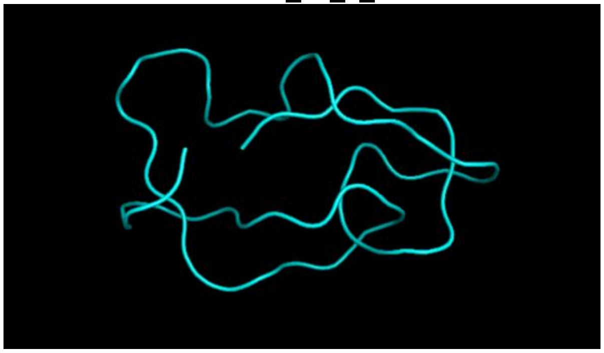

**mPLsORF0000443648_61_4_hMIN** 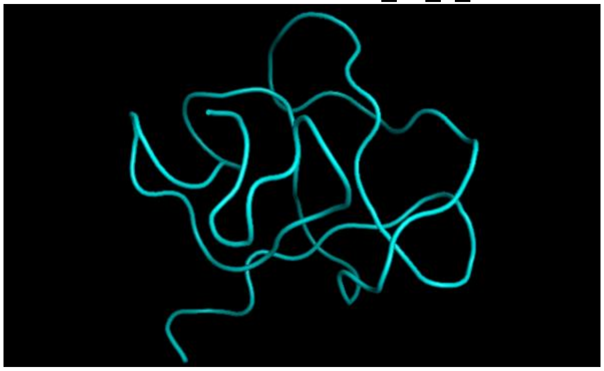	**mPLsORF0000446045_54_9_hMIN** 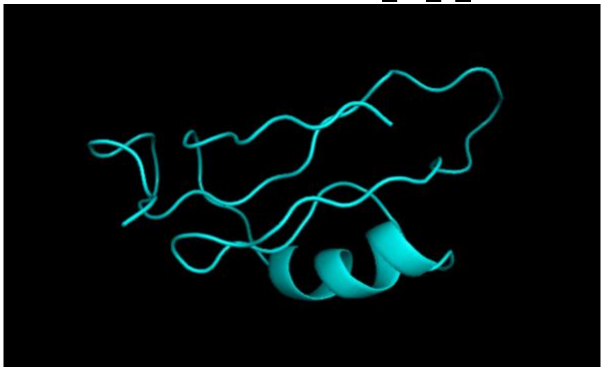
**mPLsORF0000446380_83_10_hMIN** 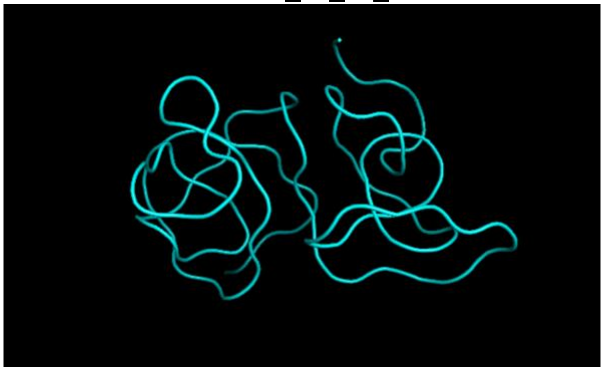	**mPLsORF0000446071_63_4_hMIN** 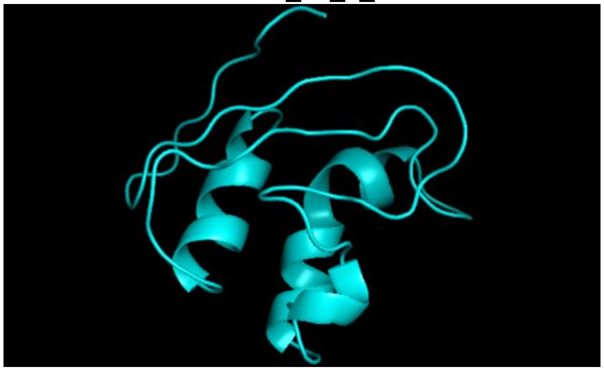
**mPLsORF0000440295_45_4_hMIN** 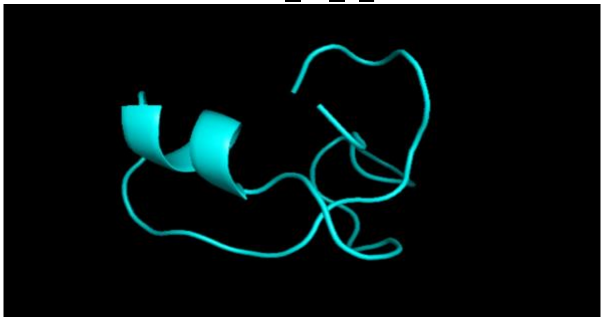	**mPLsORF0000059717_67_1_hMIN** 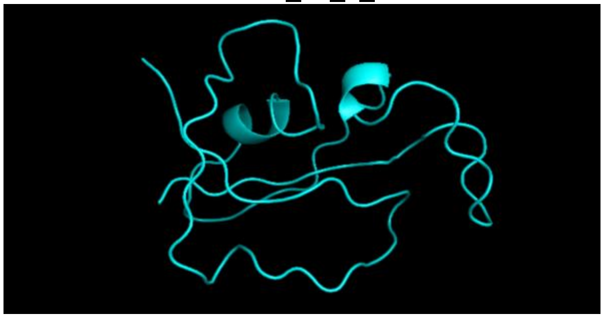

**mPLsORF0000443953_significant_ECs_0** 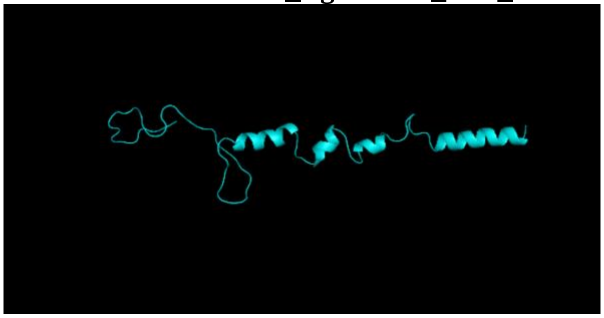	**mPLsORF0000447578_24_9_hMIN** 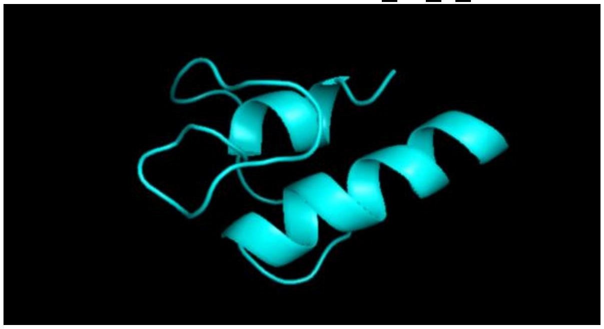
**mPLsORF0000239729_significant_ECs_0** 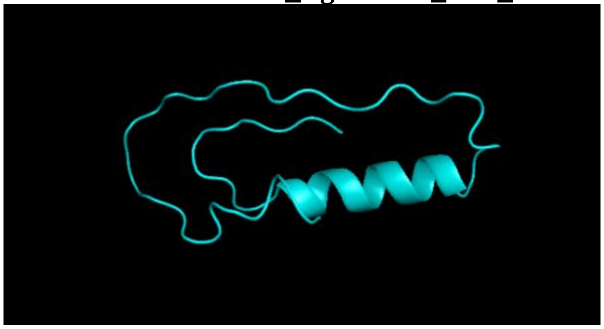	**mPLsORF0000444809_35_5_hMIN** 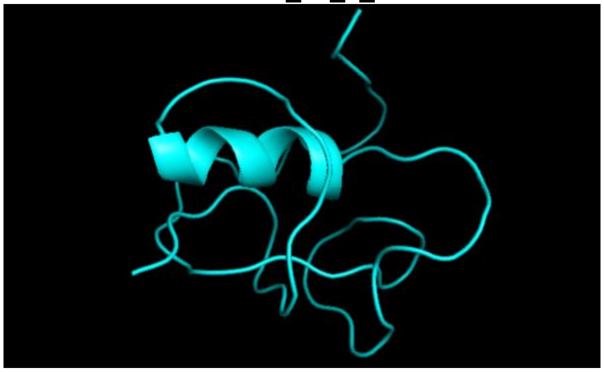
**mPLsORF0000445338_27_2_hMIN** 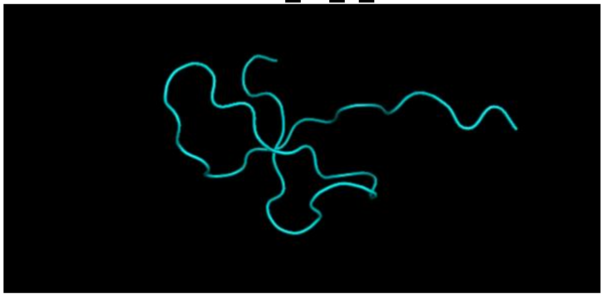	**mPLsORF0000444619_significant_ECs_0** 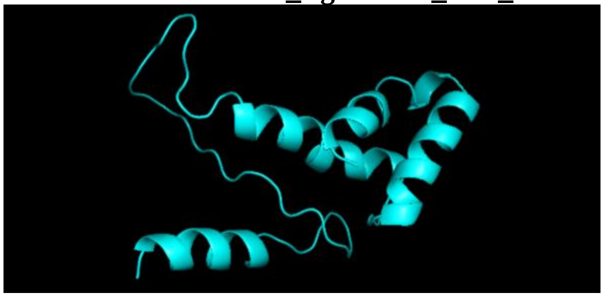

Supplementary Table 3. Predicted structures of sORFs with translational evidence. Structures of 24 sORFs for which we have both transcriptional and translational evidence and predicted with EV fold pipeline are displayed in the table.

Supplementary Table 4

**IP_220049** 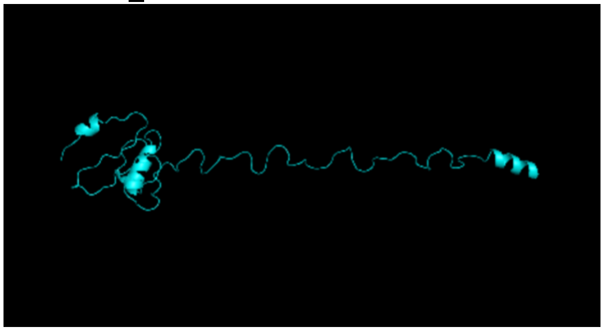	**IP_189960** 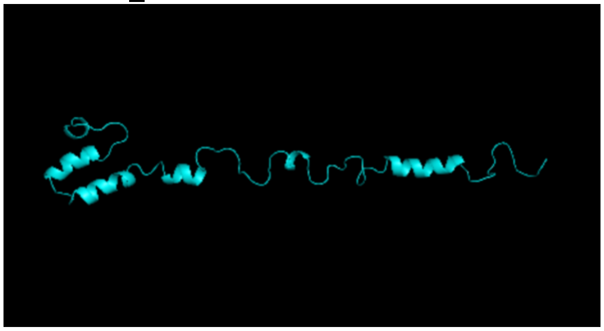
**IP_195050** 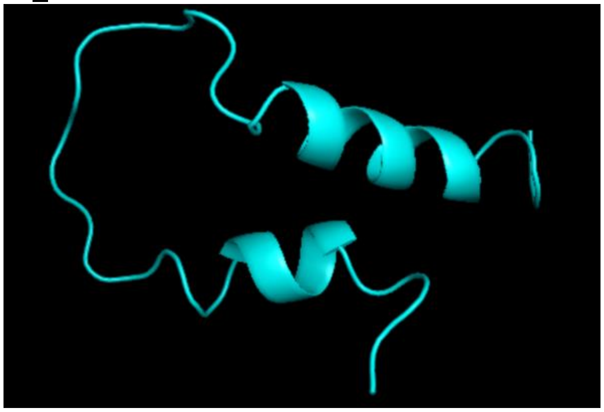	**IP_195051** 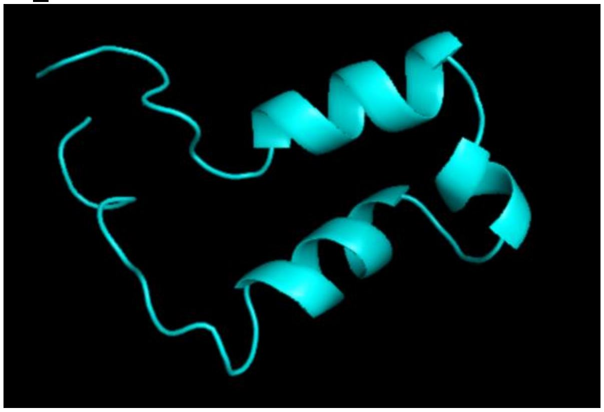
**IP_159154** 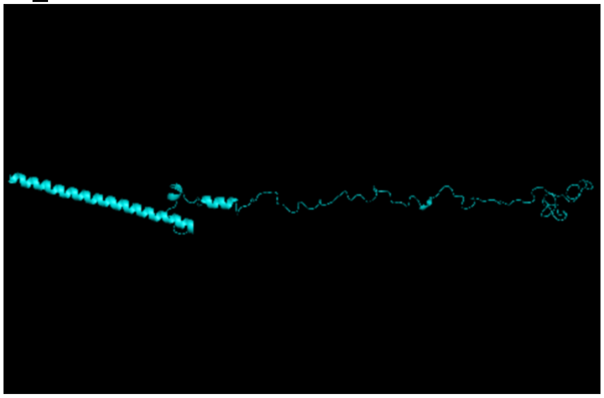	**IP_275965** 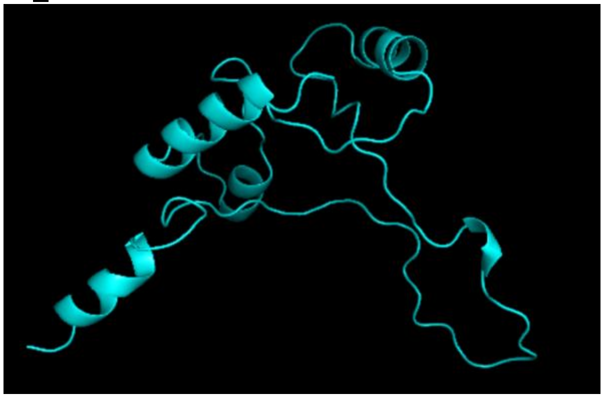

**IP_233967** 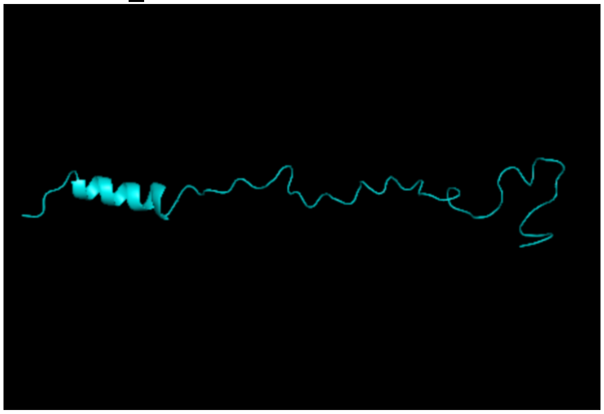	**IP_233958** 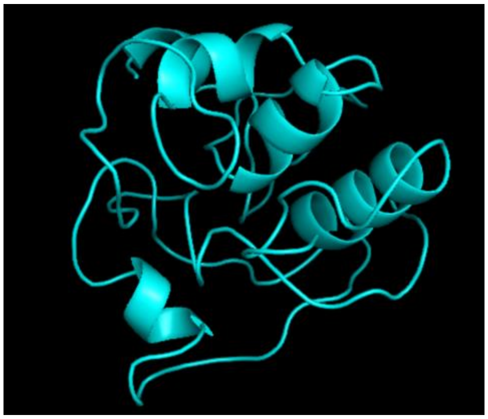
**IP_199907** 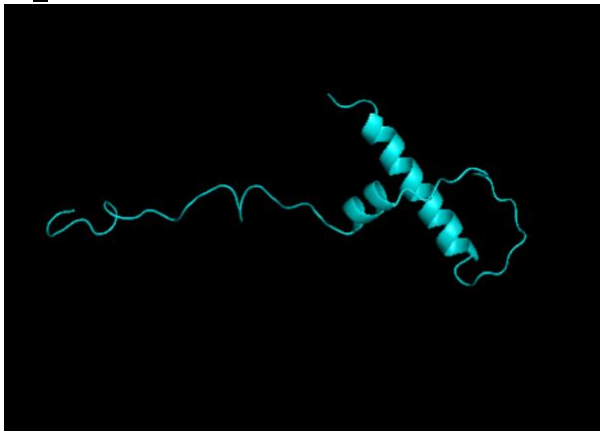	

Supplementary Table 4. Predicted structures of nine altORFs with translational evidence.

Structures of nine altORFs for which we have both transcriptional and translational evidence and predicted with EV fold pipeline are displayed in the table.

Supplementary Table 5

Mutation ID(s)	Change	Effect on sORF	SORF secondary structure
COSN19210254	115553735 T>A	K5>K	C
#COSN8491742	115553987 C>A	A89>T	C

mutation not mapped on the structure

Supplementary Table 5. The table shows the list of cosmic mutation IDs for Figure 6B along with the nucleic acid change, predicted amino acid change according to the standard amino acid code, and the predicted secondary structure of the protein at that position. C = Coil.

Supplementary Table 6

TCGA cancer abbreviation	TCGA cancer	Primary site of the tumor	Number of sample of solid tissue normal (Tnor)	Number of tumor samples (Ttum)	Primary site of GTEX samples	GTEX - site with sublocations	Number of healthy normal samples from GTEx
BLCA	Bladder Urothelial Carcinoma	Bladder	19	407	Bladder	Bladder	9
GBM	Glioblastoma Multiforme	Brain	5	166	Brain	Brain - Amygdala,Anterior Cingulate Cortex (Ba24),Caudate (Basal Ganglia),Cerebellar Hemisphere ,Cerebellum ,Cortex,Frontal Cortex (Ba9),Hippocampus,Hypothalamus,Nucleus Accumbens (Basal Ganglia),Putamen (Basal Ganglia),Spinal Cord (Cervical C-1),Substantia Nigra	1152
BRCA	Breast Invasive Carcinoma	Breast	113	1099	Breast	Breast - Mammary Tissue	179
CESC	Cervical & Endocervical Cancer	Cervix	3	306	Cervix Uteri	Cervix – Ectocervix, Endocervix	10
COAD	Colon Adenocarcinoma	Colon	41	290	Colon	Colon – Sigmoid, Transverse	308
READ	Rectum Adenocarcinoma	Rectum	10	93	Colon	Colon – Sigmoid, Transverse	308
ESCA	Esophageal Carcinoma	Esophagus	13	182	Esophagus	Esophagus - Gastroesophageal Junction, mucosa, muscularis	655
KICH	Kidney Chromophobe	Kidney	25	66	Kidney	Kidney - Cortex	28
KIRC	Kidney Clear Cell Carcinoma	Kidney	72	531	Kidney	Kidney - Cortex	28
KIRP	Kidney Papillary	Kidney	32	289	Kidney	Kidney -	28

	Cell Carcinoma					Cortex	
LIHC	Liver Hepatocellular Carcinoma	Liver	50	371	Liver	Liver	110
LUAD	Lung Adenocarcinoma	Lung	59	515	Lung	Lung	288
LUSC	Lung Squamous Cell Carcinoma	Lung	50	498	Lung	Lung	288
PAAD	Pancreatic Adenocarcinoma	Pancreas	4	179	Pancreas	Pancreas	167
PRAD	Prostate Adenocarcinoma	Prostate	52	496	Prostate	Prostate	100
STAD	Stomach Adenocarcinoma	Stomach	36	414	Stomach	Stomach	175
THCA	Thyroid Carcinoma	Thyroid Gland	59	512	Thyroid	Thyroid	279
UCEC	Uterine Corpus Endometrioid Carcinoma	Endometrium	23	181	Uterus	Uterus	78
DLBC	Diffuse large B-celll lymphoma	Lymphatic tissue	0	47	Blood	Whole Blood	337

Supplementary Table 6: Details of the cancer and matched normal tissue samples from TCGA and GTEX studies respectively, downloaded from UCSC Xena.

`

Supplementary Table 7

Software	Residues	Residues selected for docking
Castp	Pro2, Ala6, Glu7, Gly8, Lys11<br><br>Gly12, Lys14, Gln22, Arg23, Arg24<br><br>Ala30, Pro32, ARG40, Pro41,<br><br>Lys51, Arg56, Lys57, Ala60, Asn72, Gly73, Val75, Lys76, Thr77, Ala80, Gln81	Pro2, Ala6, Glu7, Gly8, Lys11, Gly12, Asp13, Thr15,<br><br>Gln22, Arg23, Arg24<br><br>Ala30, Pro32, ARG40, Pro41, Lys51, Arg56, lys57, Ala60, Asn72, Gly73, Val75, Lys76, Thr77, Ala80, Gln81
SiteMap	Pro2, Glu7, Gly8, Asp13, Thr15, Ala60, Ala80,Gln81	

Supplementary Table 7: Predicted active site residues used in docking are listed above

Supplementary Table 8

Compound Id	Docking Score
8462	-7.011
11233	-6.436
10977	-6.029
11189	-5.996
10976	-5.678
11212	-5.473
4965	-5.187
8554	-4.966
10035	-4.774
9994	-4.698
11188	-4.689
10516	-4.433
9987	-4.399
10922	-4.390
10413	-4.387
10547	-4.263
11232	-4.214

Supplementary Table 8: Table shows the top immuno-oncology library compounds and their docking scores with the nORF ENST00000427352.1.

Supplementary Table 9

Compound Id	Docking Score
1491	-7.114
139	-6.883
1479	-6.739
700	-6.662
140	-6.496
3256	-6.268
6649	-5.997
4095	-5.987
1581	-5.974
3104	-5.959
4093	-5.952

Supplementary Table 9: Table shows the top targeted-oncology library compounds and their docking scores with the nORF ENST00000427352.1.

Supplementary Table 10

Compound Id	Docking Score
1355	-7.238
129	-6.883
687	-6.662
1347	-6.631

Supplementary Table 10: Table shows the top signaling inhibitors and their docking scores with the nORF ENST00000427352.1.

Supplementary Table 11

Top Compounds	Binding energy (Kcal/mol)
8462	-38.68
11233	-45.76
10977	-45.53
11189	-33.22

Supplementary Table 11: MM-GBSA binding energies, which estimates relative binding affinities for the few best hits from immuno-oncology library compounds.

Supplementary Table 12

Top Compounds	Binding Energy (Kcal/mol)
1491	-44.31
139	-35.59
1479	-43.03
700	-38.03
140	-47.83

Supplementary Table 12: MM-GBSA binding energies, which estimates relative binding affinities for the few best hits from targeted-oncology library compounds.

Supplementary Table 13

Top Compounds	Binding energy (Kcal/mol)
1355	-7.238
129	-6.883
687	-6.662
1347	-6.631

Supplementary Table 13: MM-GBSA binding energies, which estimates relative binding affinities for the few best hits from Signaling Pathway inhibitors.
