# Supplementary material for: Pan-cancer analysis of transcripts encoding novel open-reading frames (nORFs) and their potential biological functions
Source: NPJ Genom Med. 2021 Jan 25;6:4. doi: 10.1038/s41525-020-00167-4 (PMC7835362; doi:10.1038/s41525-020-00167-4)
Supplement: Supplementary file 2 — Reporting Summary [file 41525_2020_167_MOESM2_ESM.pdf]

nature research

Corresponding author(s): *Double-blind peer review submissions: write DBPR and your manuscript number here instead of author names.*

Reporting Summary

Nature Research wishes to improve the reproducibility of the work that we publish. This form provides structure for consistency and transparency in reporting. For further information on Nature Research policies, see Authors & Referees and the Editorial Policy Checklist.

Statistical parameters

When statistical analyses are reported, confirm that the following items are present in the relevant location (e.g. figure legend, table legend, main text, or Methods section).

n/a	Confirmed	
☐	☒	The exact sample size (*n*) for each experimental group/condition, given as a discrete number and unit of measurement
☐	☒	An indication of whether measurements were taken from distinct samples or whether the same sample was measured repeatedly
☐	☒	The statistical test(s) used AND whether they are one- or two-sided<br>*Only common tests should be described solely by name; describe more complex techniques in the Methods section.*
☒	☐	A description of all covariates tested
☒	☐	A description of any assumptions or corrections, such as tests of normality and adjustment for multiple comparisons
☒	☐	A full description of the statistics including central tendency (e.g. means) or other basic estimates (e.g. regression coefficient) AND variation (e.g. standard deviation) or associated estimates of uncertainty (e.g. confidence intervals)
☒	☐	For null hypothesis testing, the test statistic (e.g. *F*, *t*, *r*) with confidence intervals, effect sizes, degrees of freedom and *P* value noted<br>*Give P values as exact values whenever suitable.*
☒	☐	For Bayesian analysis, information on the choice of priors and Markov chain Monte Carlo settings
☒	☐	For hierarchical and complex designs, identification of the appropriate level for tests and full reporting of outcomes
☒	☐	Estimates of effect sizes (e.g. Cohen's *d*, Pearson's *r*), indicating how they were calculated
☐	☒	Clearly defined error bars<br>*State explicitly what error bars represent (e.g. SD, SE, CI)*

Our web collection on statistics for biologists may be useful.

Software and code

Policy information about availability of computer code

Data collection	Almost all processed data is in the main text or in the supplementary materials. Transcriptomic data can be obtained from GEO accession GSE94671 and GSE94676. The mass spectrometry proteomics data are deposited to the ProteomeXchange Consortium via the PRIDE 66 partner repository with the dataset identifier PXD022099. The results shown are in part based upon data generated by the TCGA Research Network: https://www.cancer.gov/tcga.
Data analysis	All codes for this work can be obtained from https://github.com/PrabakaranGroup/norfs-cancer-biological-functions.

For manuscripts utilizing custom algorithms or software that are central to the research but not yet described in published literature, software must be made available to editors/reviewers upon request. We strongly encourage code deposition in a community repository (e.g. GitHub). See the Nature Research guidelines for submitting code & software for further information.

April 2018

Data

Policy information about availability of data

All manuscripts must include a data availability statement. This statement should provide the following information, where applicable:

- Accession codes, unique identifiers, or web links for publicly available datasets
- A list of figures that have associated raw data
- A description of any restrictions on data availability

Almost all processed data is in the main text or in the supplementary materials. Transcriptomic data can be obtained from GEO accession GSE94671 and GSE94676. The mass spectrometry proteomics data are deposited to the ProteomeXchange Consortium via the PRIDE 66 partner repository with the dataset identifier PXD022099. The results shown are in part based upon data generated by the TCGA Research Network: https://www.cancer.gov/tcga.

Field-specific reporting

Please select the best fit for your research. If you are not sure, read the appropriate sections before making your selection.

☒ Life sciences ☐ Behavioural & social sciences ☐ Ecological, evolutionary & environmental sciences

For a reference copy of the document with all sections, see nature.com/authors/policies/ReportingSummary-flat.pdf

Life sciences study design

All studies must disclose on these points even when the disclosure is negative.

Sample size	B and T cell transcriptome analysis were done in triplicates and for the proteomic analysis the triplicates were pooled. These statistical analysis does not impact the study or results.
Data exclusions	We did not exclude any data
Replication	This is mostly a computational work. Mass-spectrometry analysis was done on proteins extracted from B and T cells from 12 mice to show as proof of principle that pervasive translational signatures can be identified in real cells. More in-depth replication will reveal more
Randomization	n/a
Blinding	n/a

Reporting for specific materials, systems and methods

Materials & experimental systems

n/a	Involved in the study	
☒	☐	Unique biological materials
☒	☐	Antibodies
☒	☐	Eukaryotic cell lines
☒	☐	Palaeontology
☐	☒	Animals and other organisms
☒	☐	Human research participants

Methods

n/a	Involved in the study	
☒	☐	ChIP-seq
☒	☐	Flow cytometry
☒	☐	MRI-based neuroimaging

Animals and other organisms

Policy information about studies involving animals; ARRIVE guidelines recommended for reporting animal research

Laboratory animals	Spleen from six male and six female C57BL/6J mice, age 12 weeks were collected in the cold 1x PBS (-Ca, -Mg) and gently crushed on a 40µM Nylon cell strainer (Fisherbrand, 22363547) using Iscove's Modified Dulbecco's Medium (IMDM, Sigma I3390), 10% heat-inactivated FBS (Sigma F9665), 1% antibiotic + antimicotic (Sigma A5955) media, 1% L-glutamine (Sigma G7513)) to isolate splenocytes.
Wild animals	n/a
Field-collected samples	n/a
